# BRAF-Inhibitor-Induced Metabolic Alterations in A375 Melanoma Cells

**DOI:** 10.3390/metabo11110777

**Published:** 2021-11-14

**Authors:** Prashant Karki, Shayne Sensenbach, Vahideh Angardi, Mehmet A. Orman

**Affiliations:** Department of Chemical and Biomolecular Engineering, University of Houston, Houston, TX 77204, USA; pkarki@Central.UH.EDU (P.K.); sdsensen@CougarNet.UH.EDU (S.S.); vangardi@Central.UH.EDU (V.A.)

**Keywords:** cancer persisters, BRAF, melanoma, metabolomics, phenotype microarrays, drug-tolerance

## Abstract

Acquired drug tolerance has been a major challenge in cancer therapy. Recent evidence has revealed the existence of slow-cycling persister cells that survive drug treatments and give rise to multi-drug-tolerant mutants in cancer. Cells in this dynamic persister state can escape drug treatment by undergoing various epigenetic changes, which may result in a transient metabolic rewiring. In this study, with the use of untargeted metabolomics and phenotype microarrays, we characterize the metabolic profiles of melanoma persister cells mediated by treatment with vemurafenib, a BRAF inhibitor. Our findings demonstrate that metabolites associated with phospholipid synthesis, pyrimidine, and one-carbon metabolism and branched-chain amino acid metabolism are significantly altered in vemurafenib persister cells when compared to the bulk cancer population. Our data also show that vemurafenib persisters have higher lactic acid consumption rates than control cells, further validating the existence of a unique metabolic reprogramming in these drug-tolerant cells. Determining the metabolic mechanisms underlying persister cell survival and maintenance will facilitate the development of novel treatment strategies that target persisters and enhance cancer therapy.

## 1. Introduction

Metastatic melanoma makes up roughly 1% of skin cancer cases in the U.S., and yet, it is responsible for more deaths per year than all other skin cancer types combined [1]. According to data collected between 2014 and 2018 in the United States, the yearly incidence rate for melanoma was 22.8 per 100,000 [2], with an estimated recurrence rate of 8.8% [3]. Davies et al. reported rapidly accelerated fibrosarcoma B-type kinase (BRAF) mutations in 59% of melanomas from a library of cancer cell lines, with the V600E substitution being the most common [4]. The mitogen-activated protein kinase/extracellular signal-regulated kinase (MAPK/ERK) signaling pathway is a kinase cascade pathway involved in cell proliferation, in which the BRAF protein activates mitogen-activated protein kinase kinase (MEK or MAPKK) [5]. Therefore, a mutation in the BRAF protein can lead to uncontrolled cell proliferation and the spread of tumor cells. As the BRAF V600E mutation has been found in over 50% of malignant melanomas [6], BRAF inhibitors, e.g., vemurafenib, are the most common form of therapeutics administered for melanoma treatment.

It is well known that cancer cells have altered metabolic states; typical metabolic alterations include the Warburg effect (increased aerobic glycolysis activity) and increased use of glutamine to support both the tricarboxylic acid (TCA) cycle and lipid synthesis [7,8]. Melanoma cells have been shown to express hypoxia-inducible factor 1 alpha (HIF1α) at an abnormally high level under normoxia, which shifts the central carbon metabolism in favor of aerobic glycolysis [9]. The pyruvate dehydrogenase (PDH) complex also plays an important role in both metabolic rewiring and tumorigenesis in melanoma. In melanomas with the BRAF V600E mutation, Kaplon et al. found that activation or inactivation of the PDH complex leads to oncogene-induced senescence or tumorigenesis, respectively [10], highlighting the importance of the Warburg effect in melanoma progression. However, central carbon metabolism is not necessarily uniform in all melanoma cancers. Ho et al. studied the interactions of monocarboxylate transporters (MCTs) with the aerobic glycolysis and oxidative phosphorylation pathways [11] and found that some melanoma cells (within a single tumor) are less dependent on aerobic glycolysis than others. They demonstrated that MCTs can facilitate the transfer of lactate from a cell subpopulation with high levels of aerobic glycolysis to cells that are more dependent on oxidative phosphorylation, creating a heterogeneous and collaborative tumor microenvironment [11].

Drug tolerance has also been observed as a heterogeneous trait in cancer cells [12]. A subpopulation, known as persister cells, has been shown to adopt a transient drug-tolerant state in cancer cell populations [12]. These persisters cells, which are described as slow-cycling cells [13], can act as a reservoir for the emergence of multi-drug-resistant mutants and contribute to cancer relapse [14]. Recent evidence shows that persisters have unique metabolic profiles compared to the bulk cancer population [15,16]. A study conducted by Hangeur et al. showed that drug-tolerant persister cells are selectively vulnerable to the lipid hyperoxidase (GPX4) inhibitor and require the enzyme to survive ferroptosis [15]. In addition, studies have demonstrated that BRAF-inhibitor-tolerant cells shift their metabolic activity away from aerobic glycolysis and toward oxidative phosphorylation, which is mediated via fatty acid oxidation [16]. In our previous study, we explored the impacts of conventional chemotherapeutics (cytotoxic medications) on persister metabolism and found that chemotherapeutics induce a transient shift from aerobic glycolysis to oxidative phosphorylation [17]. When it comes to targeted therapeutics, melanoma persister cells are generally derived by co-treatment with BRAF/MEK inhibitors [16], making it unclear how persister metabolism is affected by each drug, individually. In this study, with the use of untargeted metabolomics, high-throughput assays (phenotype microarrays), and enzymatic measurements of glucose and lactate levels, we explored BRAF-inhibitor-induced persister metabolism. Our integrated approach allowed us to identify energy sources/substrates that are selectively preferred by vemurafenib persisters.

## 2. Results

### 2.1. Vemurafenib Persister Cells Are Slow-Cycling Cells That Are Reversibly Drug Tolerant

**Figure 1:**
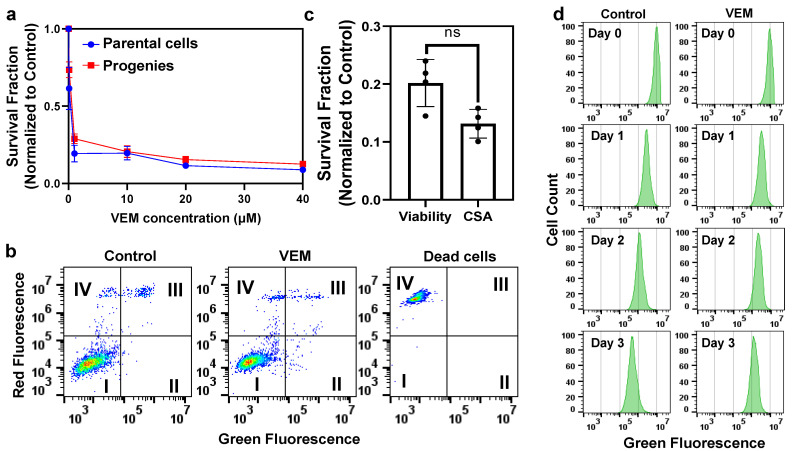
**Persisters are slow-cycling, drug-tolerant cells.** (**a**) A375 cells were treated with VEM at indicated concentrations for 3 days. After treatment, surviving cells were collected for the assessment of their viability with trypan blue staining. The survival fraction was calculated by normalizing the number of live cells in the treatment culture with the total number of cells in the untreated control group. Number of biological replicates *n* = 4. (**b**) A375 cells after VEM treatment were collected and cultured in a fresh, drug-free growth medium for 24 h. Then, the cells were stained with an annexin-V/fluorescein isothiocyanate (FITC) conjugate and propidium iodide (PI) to detect apoptotic cells. Of note, PI is a membrane-impermeant dye that can only penetrate the damaged, permeable membranes of dead cells. The cell populations were gated to represent (I) live (FITC^−^/PI^−^), (II) early apoptotic (FITC^+^/PI^−^), (III) late apoptotic (FITC^+^/PI^+^), and (IV) dead (FITC^−^/PI^+^) cells. (**c**) After treatment, A375 cells were collected to test their viability with STYO60/SYTOX green staining and clonogenic survival assays (CSA) (see Section 4). ns: not significant (*t*-test, *p* < 0.05). *n* = 4. (**d**) A375 cells pre-stained with CFSE were either treated with VEM or left untreated. At indicated time points, the fluorescence of the cells was measured with flow cytometry. *n* = 4.

Drug-tolerant persister cells were generated by treating A375 cells with vemurafenib (VEM) (Figure 1a). VEM, which is commonly used in targeted therapy for melanoma with the BRAF V600E-positive mutation, is a competitive inhibitor of the mutated BRAF protein [18]. We first treated A375 cells with different concentrations of VEM for 3 days to generate a kill curve (survival ratio vs. VEM concentration) (Figure 1a). The data show that the half maximal inhibitory concentration (IC_50_) of VEM for A375 cells was ~100 nM and a treatment concentration higher than 10 × IC_50_ did not significantly affect the persister ratio (i.e., cell survival ratio) [17]. As persisters survive high concentrations of drugs, persister cells were isolated by treating A375 cells with VEM at 100 × IC_50_ concentration for 3 days, consistent with previous studies [17,19,20]. After treatment, the cells were cultured in a fresh, drug-free growth medium for 24 h to remove dead/apoptotic cells. A cell viability imaging assay and fluorescence microscopy were used to validate that the isolated persisters were viable cells (Figure S1a). Furthermore, an annexin-V apoptosis assay was used to quantify the apoptotic cells in our samples [21]. During the early stages of apoptosis, phosphatidylserine (PS) is exposed to the outer leaflets of the cell membrane, which has high specificity toward annexin-V in the presence of calcium [22]. Our analysis showed that the isolated persister cells are not apoptotic (Figure 1b). Since persister cells are reversibly tolerant to treatments, we re-cultured these cells in a fresh medium and treated the daughter cells (i.e., progenies) with VEM (Figure 1a). As expected, the progenies of the persister cells were sensitive to VEM treatment. We also conducted clonogenic survival assays (CSAs) to assess the persister cells’ colonization capability and used live/dead cell fluorescent probes and flow cytometry to quantify persister cells after the removal of the drug. The data show that persister and colony counts were not significantly different (Figure 1c), verifying that persister cells largely colonized upon removal of VEM.

As persister cells are described as slow-cycling cells, we performed cell proliferation analysis with carboxyfluorescein succinimidyl ester (CFSE), a fluorescent dye that can readily diffuse through cell membranes and produce a stable fluorescent signal [23]. The cells were stained with the dye and cultured for 3 days either in the presence or in the absence of VEM. The cell proliferation was assessed for treated and untreated groups by monitoring the dilution of the fluorescent signal with flow cytometry. As expected, our flow cytometry analysis showed that VEM-treated cultures had retained a higher signal of fluorescence compared to the untreated control group (Figure 1d). Furthermore, we imaged CFSE-loaded cells before and after treatment with fluorescence microscopy (Figure S1b) and found that the VEM-treated cells had undergone morphological changes and retained higher green fluorescence compared to the untreated control group. This observation further supports the notion that persister cells are slow-proliferating cells, potentially induced by VEM treatment. Finally, to assess the impact of the treatment period, cells were treated with VEM (100 × IC_50_) for 9 days and the surviving cells were assessed using the aforementioned assays, showing that the effects of 9-day treatment on cell viability, morphology, and growth are very similar to those obtained from 3-day treatment (Figure S1a–c).

### 2.2. VEM Treatment Affects the Expression Levels of Cancer Stem Cell Biomarkers

**Figure 2:**
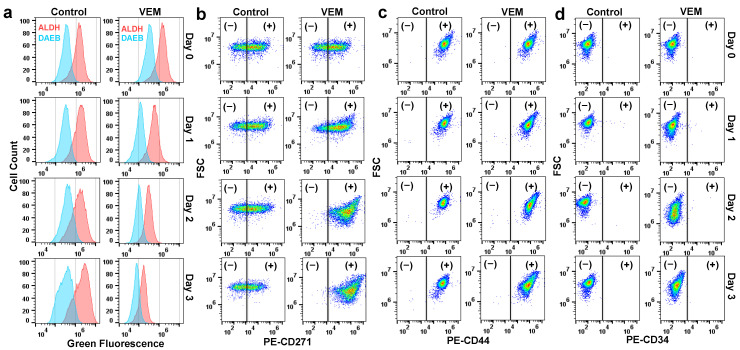
**Expression of CSC markers in persister cells.** (**a**) A375 cells were treated with VEM or left untreated for 3 days. At indicated time points, the ALDH activity of the cells (red) was assessed with the ALDEFLUOR assay. Cells treated with ALDH inhibitor 4-(dimethylamino) benzaldehyde (DAEB), highlighted in blue, were used as a negative control. (**b**–**d**) Expressions of CSC biomarkers (CD271, CD44, and CD34) were measured with their respective phycoerythrin (PE)-conjugated antibodies. Cells treated with their isotype controls were used to determine CSC-negative (−) and CSC-positive (+) cells. Live/dead staining was used to gate the live cells.

As cancer stem cells (CSCs) are often associated with dormancy, slow growth, and drug tolerance, we measured the common melanoma CSCs biomarkers (CD271, CD44, CD34, and aldehyde dehydrogenase activity) (Figure 2) to determine if the isolated persisters are CSCs [24]. There was no significant difference in CD34 and CD44 expression between persister and untreated control cells (Figure 2c,d). However, the expression level of CD271 gradually increased in VEM-treated cells when compared to untreated cells (Figure 2b). This observation has previously been reported by Rambow et al., where they showed that, upon BRAF inhibition, melanoma cells demonstrate a high expression of nerve growth factor receptor (NGFR or CD271) [25]. Additionally, it has been shown that a transient overexpression of CD271 can result in a phenotypic switch to a low proliferative and highly invasive state in cells while increasing the tolerance of the cells to BRAF inhibitors [26]. With the ALDEFLUOR assay, we measured the activity of aldehyde dehydrogenase (ALDH), a functional biomarker of melanoma stem cells, in both persisters and untreated control groups. Contrary to our expectation, VEM-treated cells showed lower ALDH activity compared to untreated control cells (Figure 2a). As CSCs have higher levels of stem cell markers than the bulk population, our observations indicate that VEM persisters are not necessarily pre-existing cancer stem cells and drug treatment can up- or down-regulate the expression levels of CSC biomarkers in the cells.

### 2.3. VEM-Induced Persister Cells Exhibit an Altered Metabolic Profile

**Figure 3:**
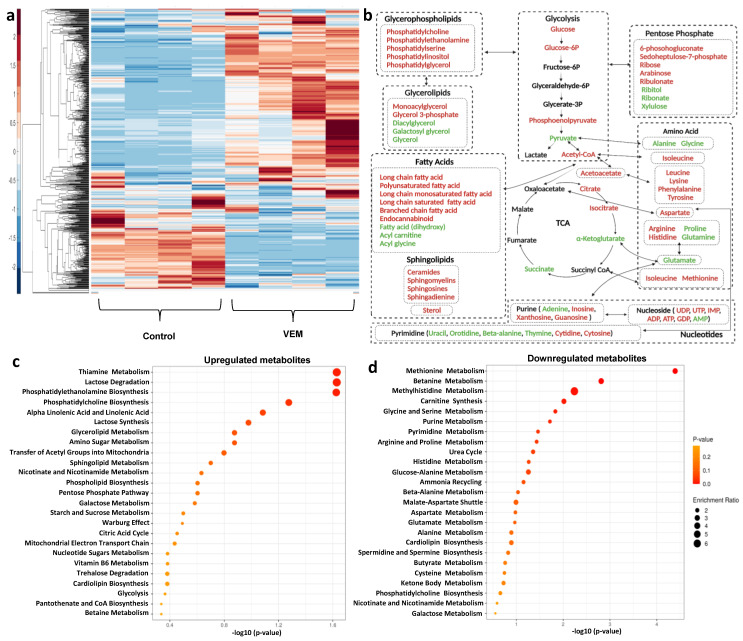
**Metabolic profiles of VEM-induced persister cells.** (**a**) VEM-treated and untreated A375 cells were collected for mass spectrometry analysis to measure their metabolite contents. The data generated with metabolomics were clustered (unsupervised) using the Clustergram function in MATLAB. Each column represents a biological replicate; each row represents a metabolite. *n* = 4. (**b**) A simplified metabolic network of persister cells (created with Biorender). Red and blue represent upregulated and downregulated metabolites in persisters when compared to the control group, respectively (ANOVA, *p* < 0.05). (**c**,**d**) Pathway enrichment analysis for all upregulated and downregulated metabolites with MetaboAnalyst, respectively.

Metabolic rewiring is a common hallmark of cancer persister cells. Persisters undergo such alterations to meet their energy requirements and enable their survival in the presence of therapeutics. These metabolic alterations can be unique or conserved in various persister phenotypes [12,15,16]. After confirming that VEM persisters are live, reversibly drug-tolerant cells that are different from CSCs (Figure 1 and Figure 2), we conducted untargeted metabolomics to study and identify persister-specific metabolic alterations. For the metabolomics study, persister cells were generated by treating the cells with VEM (100 × IC_50_) for 3 days. The metabolomics library consisted of 689 metabolites from the following super pathways: amino acids, peptides, carbohydrates, energy, lipids, nucleotides, cofactors/vitamin, and xenobiotics. The metabolomics data were normalized based on the total protein concentration, and statistical analysis was conducted with an ANOVA test (*p* < 0.05). We also performed pathway enrichment analysis with MetaboAnalyst for subsets of metabolites that were either upregulated or downregulated in persister cells (Figure 3c,d) [27]. Our data show that the concentrations of 469 metabolites were significantly altered in persister cells when compared to the untreated control group (Figure 3 and Tables S1 and S2). Furthermore, ~67.6% (317) of the identified metabolites were significantly upregulated while ~32.4% (152) were downregulated in persister cells compared to the untreated control (Table S2). 

Of the 317 upregulated metabolites, 161 were involved in the lipid super pathway. The enrichment analysis further validated that a majority of the metabolites that were upregulated in persister cells were involved in lipid biosynthesis (Figure 3b,c). Phosphatidylcholine (PC), phosphatidylethanolamide (PE), monoacylglycerol, lysophospholipid, and sphingomyelins were primarily upregulated, while metabolites involved in fatty acid metabolism were downregulated in persister cells (Figure 3a–d and Table S2). In addition to lipids, metabolites associated with lysine, purine/pyrimidine, thiamine, and lactose metabolism were upregulated, while one-carbon (betaine, glycine, and serine) metabolism, branched-chain amino acid (BCAA) metabolism, and glutamate metabolism were downregulated in persisters compared to control cells (Figure 3a–d, and Table S2). Our data also show an alteration of carbohydrate metabolism; particularly, glucose and glucose 6-phosphate were significantly upregulated in persister cells. Interestingly, although we did not observe significant alterations in metabolic intermediates of glycolysis and tricarboxylic acid (TCA) cycle, two TCA cycle substrates, alpha-ketoglutarate and succinate, were downregulated in persister cells (Figure 3b). The observed effect can be attributed to the overall alteration of amino acid metabolism as alpha-ketoglutarate and succinate are involved in various pathways, including BCAA, gamma-aminobutyric acid (GABA) shunt, and glutamate metabolism (Figure 3b). Overall, while our metabolomics data identified specific metabolites that are significantly altered in persisters, it is not clear which substrates/carbon sources persister cells efficiently use to maintain the biosynthesis of these detected molecules. Therefore, additional experiments (see below) are needed to further characterize persister cell metabolism.

### 2.4. TCA Cycle Activity of Persister Cells Is Not Significantly Different from That of Control Cells

We used phenotype microarrays (Biolog Inc., Hayward, CA, USA) to determine the metabolites that are efficiently used by persister cells. Mitoplate arrays, which mainly include TCA cycle substrates, were used to assess the rates of oxidative phosphorylation of VEM-treated and untreated control cells. This assay employs a tetrazolium dye that produces water-soluble formazan crystals when reduced by mitochondrial reductases. The rate of metabolism of specific metabolites is measured by monitoring the color change associated with formazan production (optical density at 590 nm (OD_590_)) with a plate reader. In addition, the cells are permeabilized with saponin so that the tetrazolium dye can be intracellularly reduced by mitochondria.

In our previous study, we showed that the levels of TCA metabolites are downregulated in drug-tolerant cells (obtained from chemotherapeutic treatments) because of the increased consumption rates of these intermediates by the cells [17]. From the mitoplate assays, we observed a slight increase in the consumption of succinate in VEM persister cells (Figure S2). This observation corroborates our metabolomics data, which showed an overall downregulation of succinate molecules in VEM persisters compared to the untreated control. However, no significant difference was observed in the consumption rates of other TCA cycle metabolites (Figure S2). Although drug-tolerant cells are generally shown to be less dependent on aerobic glycolysis (the Warburg effect) and more reliant on oxidative phosphorylation [16,28,29], our metabolomics and mitoplate data do not show this trend in VEM persisters.

### 2.5. Lactic Acid Consumption Is Significantly Upregulated in VEM Persister Cells

A phenotype microarray (PM-M1) (Biolog Inc.) containing 91 carbon and nitrogen substrates was used to identify metabolites that are efficiently catabolized by persisters. Similar to the mitoplate assay, cellular metabolic activities are measured by a tetrazolium dye in the PM-M1 assay. However, unlike the mitoplate assay, cells are not permeabilized in the PM-M1 assay, in which the cells are cultured for 2 days in a minimal medium supplemented with fetal bovine serum (FBS), glutamine, and the substrate of interest (see Section 4). 

**Figure 4:**
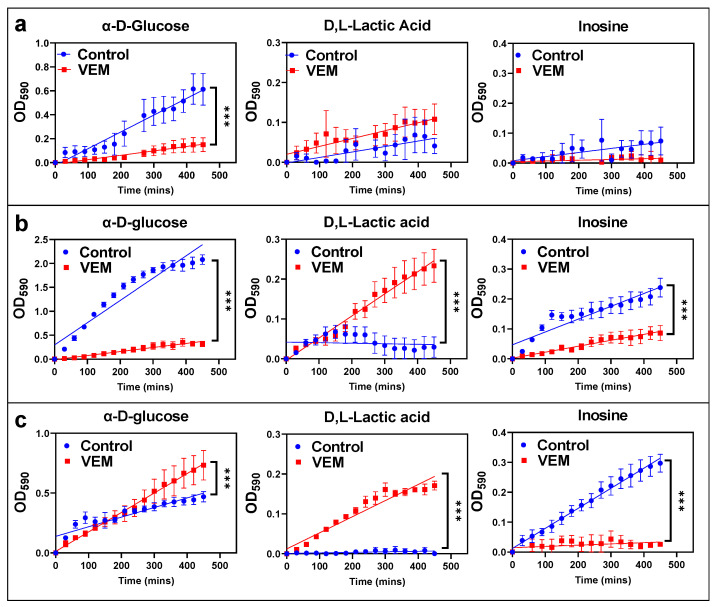
**Assessing the consumption rates of various substrates in persister cells.** Phenotype microarrays were used to measure the consumption rates of various substrates in VEM persister and control groups at (**a**) 0 h, (**b**) 24 h, and (**c**) 48 h. Consumption rates were measured with a tetrazolium-based dye at 590 nm (OD_590_). The absorbance was normalized by using t = 0 h and glutamine control data (see Section 4). Data points and error bars represent the mean and the standard error, respectively. Statistical analysis was performed using a linear regression analysis (F-Statistics, *** *p* < 0.001). *n* = 4.

After transferring VEM-treated and untreated control cells to PM-M1 arrays, metabolic activities of the cells were measured at t = 0 h, 24 h, and 48 h with the tetrazolium dye. We observed that carbon sources such as D-turanose, D-mannose, glycogen, and D-maltose were consumed less by persisters when compared to control cells (Figures S3–S5). We also observed significant differences in glucose, lactic acid, inosine, and adenosine metabolism between persisters and untreated cells (Figure 4 and Figures S3–S5). Interestingly, although untreated cells seemed to have higher consumption rates of glucose at t = 0 h and 24 h, the glucose consumption rates were found to be low in persister cells at initial time points (Figure 4). Increased intracellular concentrations of glucose and glucose 6-phosphate in persister cells determined by metabolomics analysis (Figure 3b) may be due to the decreased consumption rates of these metabolites by the persister cells (Figure 4). However, lactate metabolism is significantly upregulated in persister cells (Figure 4), while the metabolism of inosine and adenosine is more active in control cells (Figure 4 and Figures S3–S5). Lactic acid is a byproduct of glycolysis and glutaminolysis, and increased levels of lactate production in cancer cells have been extensively studied and linked with increased aerobic glycolysis. However, the observed difference in lactic acid use (Figure 4) may be due to the conversion of lactic acid to pyruvate and acetyl-coA, which serve as metabolic intermediates for lipid and amino acid metabolism in persister cells (Figure 3b). Lastly, both adenosine and inosine are nucleosides that have been linked to cell proliferation and cell death in cancer cells [30] and both metabolites are preferred by untreated control cells (Figure 4). This observation is in alignment with our metabolomics data, as we see an accumulation of inosine- and adenosine-containing metabolites in persister cells, potentially due to their reduced consumption rates in the cells. 

### 2.6. Persister Cells Have Increased Viability in a Minimal Medium

**Figure 5:**
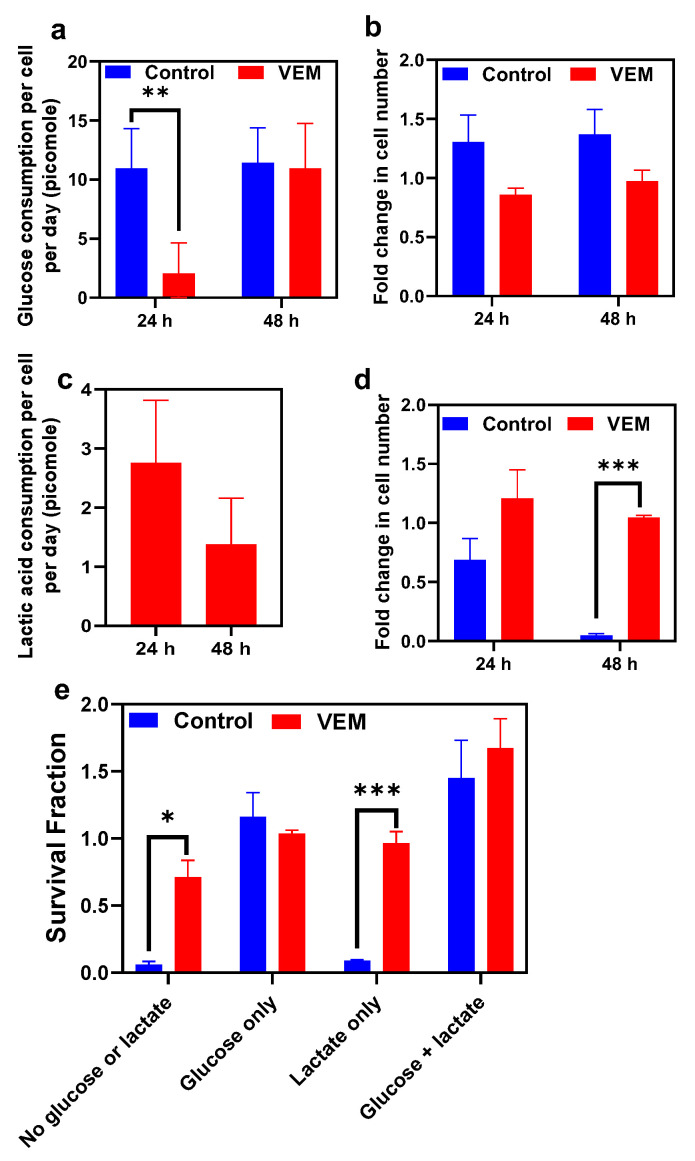
**Measurement of glucose and lactic acid consumption.** After treatment with VEM, cells were cultured in a minimal medium consisting either glucose or lactic acid. (**a**–**d**) The substrate consumption rates and live cell numbers were measured for cultures consisting of glucose (**a**,**b**) or lactic acid (**c**,**d**) at indicated time points. *n* = 4. (**e**) Untreated or VEM-treated cells were cultured in various conditions to assess their viability. *n* = 4. Data points and error bars represent the mean and the standard error, respectively. Statistical comparison was conducted with a pairwise *t*-test (* *p* < 0.5, ** *p* < 0.01, and *** *p* < 0.001).

To further validate the phenotype microarray data, we measured glucose and lactic acid concentrations in the cultures in which VEM-treated and untreated control cells were cultured in the presence of glucose or lactic acid under PM-M1 assay conditions. Our results show that while glucose consumption at t = 24 h was significantly higher in untreated control cells compared to VEM persisters, the glucose consumption of persister cells increased to a level similar to the untreated control by 48 h (Figure 5a), consistent with our PM-M1 results (Figure 4). Both glucose consumption and phenotype microarray data verify the existence of a longer lag phase in persister cells. We also measured the number of viable cells (although confluent cell cultures were used for PM-M1 assays as described in Section 4) and showed that the number of cells was slightly higher in the control group in the presence of glucose; however, the difference between persister and control groups was not statistically significant (Figure 5b). Our measurement of lactic acid in untreated control samples showed minimal or no changes in lactic acid concentration; therefore, we were not able to calculate the lactic acid consumption by control cells. However, VEM persister cells were able to consume lactic acid (Figure 5c), as predicted by PM-M1 data (Figure 4). Interestingly, cell count data showed that, at 48 h, the survival ratio of untreated control cells was significantly lower compared to that of VEM persister cells in a minimal medium containing lactic acid (Figure 5d).

To comprehensively study the effects glucose and lactic acid on cell viability, we cultured the untreated or VEM-treated cells for 48 h in the minimal medium consisting of FBS and glutamine and supplemented with either lactic acid, glucose, or both substrates. Then, at 48 h, the cells were stained with live/dead probes and analyzed with a flow cytometer to enumerate live cells. The survival fraction was then calculated by normalizing the cell counts with the initial number of cells (Figure 5e). As expected, the viability of both control and persister cells was found to be higher when the medium had glucose. However, the viability of the untreated control group dropped significantly in the absence of glucose and the presence of lactic acid in the minimal medium that lacked glucose did not improve the viability of control cells (Figure 5e). On the contrary, the viability of VEM persisters was not affected by lactic acid and persister cells can still survive in a minimal medium supplemented with FBS and glutamine without glucose and lactic acid (Figure 5e). These observations further highlight the significance of metabolic reprogramming for persister cell survival. 

## 3. Discussion

The emergence of drug-tolerant persister cells has become one of the major challenges in cancer treatment. The ability of persister cells to avoid conventional therapeutics can lead to a high relapse rate and poses a significant challenge in the complete eradication of tumor cells. With this study, we aimed to characterize the metabolic profiles of persisters that are tolerant to vemurafenib, a BRAF inhibitor. Identifying the metabolic pathways of tolerant cells can offer potential therapeutic targets, which has been an overarching goal of many recent studies. Methods employing various transcriptomics techniques (RNA-seq, single-cell RNA-seq, DNA microarrays) have been used to explore the metabolic alterations in persister cells [12,15,16]. Our study employed untargeted metabolomics, which can provide a global/comprehensive analysis of the persister metabolome. Although recent studies have explored the advantages of metabolic flux analysis with isotope-labeled metabolites, this does not provide a broad overview of the persister metabolome.

Although untargeted metabolomics is a powerful tool [31], discerning whether the identified metabolites are consumed by the cells or are the products of certain pathways is quite challenging. To address this issue, we employed phenotype microarray assays that measure the utilization rates of various substrates via a tetrazolium dye. The key underlying mechanism of this assay is that the utilization rate of a substrate correlates with the rate of dye reduction; thus, these assays in combination with metabolomics can allow us to infer whether a given biochemical source is being catabolized. In addition, we employed a modified version of the microarray assay, namely the mitoplate assay, which measures the consumption rates of substrates associated with the TCA cycle. The modified version of the tetrazolium dye is expected to be reduced by electrons primarily released from cytochromes. This technique can indeed be used to infer the rate of oxidative phosphorylation in the sample being tested. The most popular method for assessing oxidative phosphorylation is the seahorse assay, which measures the oxygen consumption rate (OCR) as well as the glycolytic flux [32]. However, the seahorse assay does not provide insights into the utilization rates of substrates contributing to the increased OCR.

Our metabolic analysis showed that almost half of the upregulated metabolites in persister cells are associated with lipid metabolism. Our subsequent assays indicated that carbon sources, such as lactate, are potentially diverted to anabolic pathways associated with lipid and amino acid metabolism in persisters. Lactate is used for many cellular processes involved in metastasis, angiogenesis, and, more importantly, immunosuppression of cancer [33]. Furthermore, lactic acid can be reversibly converted to pyruvate and acetyl-coA, which may act as an energy source for fatty acid oxidation [34] or serve as metabolic intermediates for certain amino acid and lipid metabolism (Figure 3b). 

The alteration of lipid metabolism in drug-tolerant cells has been shown by prior studies [35]. PC, PE, lysophospholipid, and sphingomyelins are major components of cell membranes and play significant roles in cell proliferation, apoptosis, and cell migration [36]. Phospholipids and sphingolipids are also closely associated with drug resistance and tumor progression in multiple cancers, including melanoma [37]. Delgado-Goni et al. demonstrated that limiting the exogenous lipid content results in an overall increase in VEM sensitivity for resistant BRAF-mutated melanoma cells [38]. De novo fatty acids are continually used by cancer cells to synthesize lipids required for membrane production and to provide energy through β-oxidation [39]. 

Nucleotide metabolism (pyrimidine and purine) represents another class of metabolites that was altered in persister cells. With microarray experiments, we observed that persister cells had lower consumption rates for adenosine and inosine compared to untreated cells. This difference was noticeably greater in the inosine consumption rate. Inosine can be produced by the catabolism of adenosine. Soares et al. have shown that adenosine causes cell proliferation by activating the adenosine receptor (AR), while inosine enhances proliferation by activating the receptor A_3_AR [40]. One-carbon metabolism, which was shown to be downregulated in persister cells, can be used for the biosynthesis of nucleotides [41]. Along with the pentose phosphate pathway (PPP), one-carbon metabolism is involved in NADPH production and the maintenance of redox and methylation states is required for cell proliferation [42]. Lastly, in amino acid metabolism, metabolites involved in BCAA were significantly altered in persister cells. BCAA metabolism in cancer has been extensively studied as it is required for many cellular processes, including protein synthesis and energy production [43,44]. Branched-chain aminotransferase 1/2 (BCAT1/ BCAT1) enzymes, which are involved in BCAA degradation, are proposed to be a prognostic marker for cancer [45]. A study by Wang et al. also demonstrated that epigenetic upregulation of BCAT1 can promote tolerance to tyrosine kinase inhibitor in lung cancer cells [20].

Along with lipid metabolism, we expected that persister cells would have a significant difference in energy metabolism, primarily in the TCA cycle and oxidative phosphorylation. Studies have shown that persister cells generally exhibit an increased rate of oxidative phosphorylation compared to bulk tumor populations [16,28]. However, in this study, we did not see a significant difference in the consumption rates of TCA substrates except for succinate. This can be a result of differences in experimental details between studies. For instance, we generated persister cells with a single drug treatment while the majority of the studies have used combinations of BRAF/MEK inhibitors and/or chemotherapeutics. A study conducted by Parmenter et al. showed similar outcomes where a single treatment with a BRAF inhibitor resulted in a decrease in the OCR of melanoma cells [46], although they speculated that long-term treatments may eventually shift persister metabolism toward oxidative phosphorylation.

In this study, we showed that VEM persisters can use lactate more efficiently than untreated control cells. Although investigating the molecular mechanism underlying this observed phenomenon is beyond the scope of this study, we hypothesize that targeted therapeutics (e.g., the BRAF inhibitor, VEM) induce cell dormancy by directly inhibiting cell-proliferation signaling pathways. Oncogenic mutations in RAS genes (KRAS, NRAS, and HRAS) or RAF genes (RAF-1, BRAF, and A-RAF) occur in many cancer types, including the melanoma cells [47]. BRAF is a serine/threonine-protein kinase and acts upstream in several signaling pathways (e.g., MEK/ERK and PI3K/AKT) that promote aerobic glycolysis and enhance the expression of enzymes involved in anabolic pathways (e.g., protein and lipid synthesis) by activating various transcription factors, such as HIF1/2, MYC, FoxO, and STAT3 [47,48]. These transcription factors are known to induce glucose transporters and glycolytic and anabolic enzymes [49,50,51,52,53]. Studies show that the metabolism of drug-tolerant melanoma cells may be associated with increased mitochondrial oxidative phosphorylation and decreased glycolysis and lactate synthesis [54,55,56,57], which may indeed increase lactate consumption in the cells. Lactate consumption can interrupt cellular energy homeostasis by deactivating AMP-activated protein kinase (AMPK), resulting in the overexpression of regulatory element-binding protein 1 (SREBP1), stearoyl-CoA, and desaturase-1 [58]. Upregulation of these enzymes protects cells against ferroptosis by increasing the production of exogenous monounsaturated fatty acids [58]. 

The existence of persisters is a major obstacle in cancer therapy. Eradication of these drug-tolerant cells is a monumental challenge because the mechanisms underlying their formation and survival are highly complex and diverse. The therapeutic promise of targeting a metabolic mechanism in persister cells garners noteworthy attention in the field, as metabolism represents a rich source of targets for anti-persister strategies [15,16]. Although we focused on melanoma cells, the suggested methodologies integrating untargeted metabolomics and phenotype microarrays can be readily extrapolated to other cell lines, enabling the assessment of the physiological capabilities of a wide variety of persisters.

## 4. Materials and Methods

### 4.1. Cell Culture Conditions 

The melanoma A375 cell line was purchased from American Type Culture Collection (ATCC) (Manassas, VA, USA). If not otherwise specified, all materials were obtained from Thermo Fisher Scientific (Waltham, MA, USA). Melanoma cells were cultured in Dulbecco’s modified Eagle’s medium (DMEM) supplemented with 10% fetal bovine serum (FBS), 100 units of penicillin, and 100 µg streptomycin/mL. Cells were cultured in a humidified incubator at 37 °C and 5% CO_2_. Phenotype microarray plates and assay solutions were purchased from Biolog, Inc. (Hayward, CA, USA). PE-conjugated antibodies (CD271 (catalog# 557196), CD44 (catalog# 555479), or CD34 (555822)) and their IgG isotype controls were obtained from BD science (San Jose, CA, USA). The ALDEFLUOR assay kit was obtained from STEMCELL Technologies (Cambridge, MA, USA). The BRAF inhibitor, vemurafenib (VEM), was dissolved in dimethylsulfoxide (DMSO) to prepare stock solutions. 

### 4.2. Generating Persister Kill Curves

To generate biphasic kill curves, 3 × 10^5^ cells were seeded in each well of a 6-well plate. The cells were maintained in 3 mL of DMEM for 24 h in a humidified incubator at 37 °C and 5% CO_2_. After 24 h, the medium was removed and replaced with a fresh medium with varying concentrations of VEM. After the cells were treated with VEM for 3 days, the surviving cells were washed with 3 mL of Dulbecco’s phosphate-buffered saline (DPBS) two times. The cells were detached with 300 µL of trypsin-EDTA (0.25% trypsin and 0.9 mM EDTA) and then collected in 1.5 mL microcentrifuge tubes after adding 1 mL of DMEM, which deactivates the trypsin. The cell suspension was centrifuged at 900 revolutions per minute (rpm) for 5 min, and the supernatant was removed. The cell pellet was then re-suspended in fresh DMEM, and live cells were quantified with trypan blue staining [59] using an automated cell counter (catalog# A27977, Thermo Fisher Scientific, Waltham, MA, USA). The survival ratio was calculated by normalizing the cell count with the total number of cells in the untreated control group. Finally, the survival ratios and drug concentrations were plotted to generate the kill curves.

### 4.3. Isolating Persister Cells

Approximately 2.5 × 10^6^ cells were transferred to 15 mL of DMEM in a T-75 flask. The cells were cultured for 24 h in a humidified incubator maintained at 37 °C and 5% CO_2_. After 24 h, the medium was replaced with a fresh medium containing 10 μM VEM. After the cells were treated with VEM for 3 days, they were washed with 10 mL of DPBS two times and detached from the flasks with 2 mL of trypsin-EDTA. Then, 5 mL of DMEM was added to deactivate the trypsin and the cells were centrifuged at 900 rpm for 5 min. The supernatant was removed, and the cells were resuspended in fresh DMEM and transferred to a T-75 flask. The cells were further incubated at 37 °C in the presence of 5% CO_2_ for 24 h to remove dying/dead cells as they cannot adhere to the flask surface. Following the incubation, the supernatant with dead cells was discarded and the live cells were detached and collected for the experiments described below. The control group (untreated cells) underwent the same procedure, receiving the solvent-only (DMSO) treatment. 

### 4.4. Assessing the Cell Viability with Microscopy 

The viability of cells after VEM treatment was assessed with the ReadyProbes Cell Viability Imaging kit (Blue/Green) (catalog# R37609, Thermo Fisher Scientific) as described by the vendor’s protocol. The viability imaging kit consists of cell-permeant NucBlue and cell-impermeant NucGreen dyes for visualizing live (blue) and dead (blue + green) cells. DAPI (excitation: 360 nm; emission: 460 nm) and GFP (excitation: 470 nm; emission: 525 nm) channels from EVOS M7000 fluorescence microscopy (catalog# AMF7000, Thermo Fisher Scientific) were used to measure the fluorescence signals. Untreated and dead cells were used as negative and positive controls, respectively. Cells were treated with 70% ethanol for 30 min to generate dead cells.

### 4.5. Apoptosis Assay

The annexin-V FITC/PI kit (catalog# P50-929-7, Thermo Fisher Scientific) was used to detect apoptotic cells in VEM-treated and untreated cultures, as described in our previous study [17]. Briefly, cells surviving the treatment were collected and centrifuged at 900 rpm for 5 min and the supernatant was removed. The cells were washed with 5 mL of DPBS. After washing, the cells were centrifuged and resuspended in a 1× binding buffer to achieve a density of 5 × 10^5^ cells per mL. Then, 195 µL of the cell suspension was transferred to a 1.5 mL microcentrifuge tube. Next, 5 µL of annexin-V FITC solution was added to the microcentrifuge tube and the cell suspension was incubated for 10 min at room temperature. The cell suspension was then centrifuged for 5 min at 900 rpm, and the supernatant was removed. The cell pellet was resuspended in 190 µL of a 1× binding buffer, and 10 µL of propidium iodide (PI) was added for detecting dead cells in the sample. Finally, the cell suspension was transferred to a 5 mL test tube followed by the addition of a 1× binding buffer to achieve a final volume of 500 µL. The samples were then analyzed with a flow cytometer to measure green (excitation: 488 nm; emission: 520 nm) and red (excitation: 561 nm; emission: 586 nm) fluorescence. The flow cytometry gates for four different cell subpopulations were determined as (I) FITC^-^/PI^-^ (II) FITC^+^/PI^-^ (III) FITC^+^/PI^+^, and (IV) FITC^-^/PI^+^, representing live, early apoptotic, late apoptotic, and dead cells, respectively. Untreated live cells and ethanol-treated cells were used as negative and positive controls to determine the gates.

### 4.6. Clonogenic Survival Assay 

Cells surviving the VEM treatment were serially diluted by transferring 1 mL of the cell suspension to a 15 mL centrifuge tube containing 9 mL of a fresh growth medium (i.e., a 10-fold serial dilution). Then, 3 mL of diluted cell suspensions was transferred to the wells of a 6-well plate. The cells were cultured for 12 days for colony formation while the growth medium was replaced every 3–4 days. After 12 days, the growth medium was removed and the cells were washed with 3 mL of DPBS. The cells were then fixed with 1 mL of a fixing agent (methanol: acetic acid at 3:1) for 5 min. After the cells were fixed, they were stained with crystal violet (0.5%) for 15 min to visualize and to count the colonies. 

### 4.7. Cell Count with a Flow Cytometer 

After VEM treatment, the live cells were collected and washed with DPBS twice and resuspended in DPBS. Then, 1 mL of the cell suspension was transferred to a 1.5 mL microcentrifuge tube. The cells were stained with 0.25 µM SYTOX green (catalog # S7020, Thermo Fisher Scientific, Waltham, MA, USA) and SYTO60 red (catalog # S11342, Thermo Fisher Scientific), followed by incubation at 37 °C for 15 min. Then, the cells were transferred to a 5 mL test tube and analyzed with a flow cytometer to measure green (excitation: 488 nm; emission: 520 nm) and red (excitation: 561 nm; emission: 586 nm) fluorescence. SYTOX green is cell impermeant and can only diffuse through a dead cell membrane while SYTO60 is cell permeant and can freely diffuse through both live and dead cell membranes. Untreated and ethanol-treated dead cells were used as negative and positive controls.

### 4.8. Cancer Stem Cell Markers 

Cells during VEM treatment in flasks were detached and collected at desired time points and centrifuged at 900 rpm for 5 min. The supernatant was removed, and the cell pellet was washed with 5 mL of DPBS. Then, 1 × 10^6^ cells were resuspended in 100 µL of a cell stain buffer (catalog# 554657) in a microcentrifuge tube. Next, 20 µL of PE-conjugated antibodies (CD271 (catalog# 557196), CD44 (catalog# 555479), or CD34 (555822); BD Biosciences, San Jose, CA, USA) or isotype controls were added to the cell suspensions and the suspensions incubated for 30 min in dark at room temperature. Following the incubation, the cells were centrifuged at 900 rpm for 5 min and the supernatant was discarded. Finally, the cells were resuspended in 500 µL of a cell stain buffer and transferred to a 5 mL test tube. The cells were then stained with 0.25 µM SYTOX green for 15 min at room temperature and analyzed with a flow cytometer to measure green (excitation: 488 nm; emission: 520 nm) and red (excitation: 561 nm; emission: 586 nm) fluorescence, respectively.

### 4.9. ALDEFLUOR Assay 

At desired time points, cells during VEM treatment were detached, collected, and centrifuged at 900 rpm for 5 min. The supernatant was removed, and the cell pellet was washed with 5 mL of DPBS. The cells were then resuspended in ALDEFLUOR buffer to obtain a density of 1 × 10^6^ cells per mL. Then, 1 mL of the cell suspension was transferred to a 1.5 mL microcentrifuge tube and 5 µL of activated ALDEFLUOR reagent was added to the cell suspension. After the cell suspension was mixed, half of it was transferred to a new microcentrifuge tube and immediately treated with 5 µL of diethylamino-benzaldehyde (DEAB). This sample was used as a negative control as DEAB inhibits ALDH activity. After the samples were incubated for 45 min at 37 °C, the cells were centrifuged at 900 rpm for 5 min and the supernatant was removed. The cell pellet was then resuspended in ALDEFLUOR buffer at 4 °C, transferred to a 5 mL test tube, and stained with 1.5 µM PI for 10 min. Finally, the cells were analyzed with a flow cytometer to measure green (excitation: 488 nm; emission: 520 nm) and red (excitation: 561 nm; emission: 586 nm) fluorescence, respectively.

### 4.10. Cell Proliferation Assay

A cell proliferation assay was conducted with the CellTrace proliferation kit (catalog# C34570, Thermo Fisher Scientific) following the protocol provided by the vendor. A375 cells were suspended in DPBS to attain a cell density of 1 × 10^6^ cells per mL. Then, 1 µL of carboxyfluorescein succinimidyl ester (CFSE) dye was added to 1 mL of the cell suspension and the cells were incubated at room temperature for 20 min. After incubation, DMEM medium was added to the cell suspension to stop the staining and the cells were centrifuged at 900 rpm for 5 min. The supernatant was removed, and the pellet was resuspended in fresh DMEM medium. Approximately 3 × 10^5^ of CFSE-stained cells were plated in each well of a 6-well plate and incubated for 24 h. After 24 h, the cells were either treated with 10 µM of VEM or left untreated. At indicated time points, cells were collected, centrifuged, and resuspended in 1 mL of DPBS. The cells were then stained with 1.5 µM of PI to detect dead cells. The cells were transferred to a 5 mL test tube and analyzed with a flow cytometer to measure green (excitation: 488 nm; emission: 520 nm) and red (excitation: 561 nm; emission: 586 nm) fluorescence, respectively.

### 4.11. Metabolomics

Cells were treated with VEM in T-75 flasks for 3 days. After 3 days, the surviving cells were collected, washed with 5 mL of DPBS twice, and pooled in a 1.5 mL micro centrifuge tube. The final cell pellet volume for each group was ~100 µL. The cell pellet was first flash-frozen in a dry ice/ethanol bath and then stored in −80 °C before the sample was shipped to Metabolon, Inc. (Morrisville, NC, USA). Cells treated with the solvent (DMSO) only were used as a control group. A metabolomics study including sample extraction, instrumentation, and initial data analysis was conducted according to Metabolon’s protocols [60]. The mass spectrometry data were normalized to the protein concentration (assessed by Bradford assay). The normalized data were used for unsupervised hierarchical clustering with the Clustergram function of MATLAB. An ANOVA test was used for pairwise comparisons (*p* < 0.05), and a Q-value for each metabolite was assessed to estimate the false discovery rate [60]. Enrichment analysis was performed using the overrepresentation analysis (ORA) algorithm of MetaboAnalyst [27]. The metabolites that were significantly different in VEM persisters compared to those in the control group were clustered into two groups: upregulated and downregulated metabolites. The list of compound names (human metabolome database ID) for each group was used as an input for the enrichment analysis, which is based on several libraries containing ~9000 biologically meaningful metabolite sets. ORA uses hypergeometric test to evaluate if a specific metabolite set or pathway is truly (not by chance) represented within the input list. Finally, the results were provided as dot plots, where the size of the circle represents the enrichment ratio (observed hits/predicted) and the color represents the *p*-value for each metabolite set [61].

### 4.12. Mitoplate Assays

Mitoplate assays were performed following the protocol provided by Biolog, Inc. [62]. After VEM treatment, the cells were collected, resuspended in a fresh drug-free growth medium, and incubated for 24 h. After incubation, the cells were collected and washed with DPBS twice. The cells were then suspended in a 1× Biolog mitochondrial assay solution (BMAS, catalog# 72303, Biolog, Inc.) to obtain a cell density of 1 × 10^6^ cells per mL. For assessing the mitochondrial activity, an assay mixture containing 2× BMAS, a tetrazolium-based 6× dye mix (catalog# 74353, Biolog, Inc.), saponin (960 µg/mL), and sterile deionized (DI) water in a ratio of 6:4:1:1 was prepared and 30 µL of the assay mixture was pipetted into each well of the mitoplate containing various TCA cycle substrates. The plate was then incubated for 1 h at 37 °C to completely dissolve the substrate. Finally, 30 µL of the cell suspension in 1× BMAS was added to each well of the microarray. To assess the rate of consumption of these substrates, optical density at 590 nm (OD_590_) was measured with Varioskan Lux Microplate Reader (catalog# VLBL00GD0, Thermo Fisher Scientific) every 10 min for 100 min.

### 4.13. PM-M1 Assays

PM-M1 assays were performed using a protocol provided by Biolog, Inc. [62]. After VEM treatment, the cells were collected; resuspended in a fresh, drug-free growth medium; and incubated for 24 h. At 24 h, the cells were washed with 5 mL of DPBS twice. The cell pellet was resuspended in Biolog IF-M1 medium (catalog# 72301, Biolog, Inc.) supplemented with penicillin/streptomycin, 5% FBS, and 0.3 mM glutamine to obtain a cell density of 4 × 10^5^ cells per mL. Then, 50 µL of the cell suspension was transferred to each well of the PM-M1 plate and incubated at 37 °C for 48 h. At desired time points (t = 0 h, 24 h, and 48 h), 10 µL of the tetrazolium-based dye (catalog# 74352, Biolog, Inc.), was added to each well of the PM-M1 plate and incubated for 8 h. The rate of dye reduction was evaluated by measuring absorbance at 590 nm (OD_590_) every 30 min. The absorbance readings are subtracted by the baseline (i.e., initial absorbance at t = 0 min) to determine the absolute absorbance change for each substrate. These data are then normalized by subtracting control (glutamine only) measurements.

### 4.14. Glucose and Lactate Consumption Assay

After VEM treatment, cells were collected and resuspended in Biolog IF-M1 medium (catalog# 72301, Biolog, Inc.) supplemented with penicillin/streptomycin, 5% FBS, and 0.3 mM glutamine. When indicated, 4 mM sodium lactate and/or 4 mM glucose were added to the cell suspension. The cell density was adjusted to 4 × 10^5^ cells per mL. Then, 500 µL of the cell suspension was transferred to each well of a 24-well plate and incubated at 37 °C. At desired time points (t = 24 h and 48 h), the cell suspension was removed and centrifuged at 900 rpm for 5 min and the supernatant was transferred to a 1.5 mL microcentrifuge tube. The glucose and lactate concentrations in samples and standard solutions were measured using a glucose colorimetric detection kit (catalog# EIAGLUC, Thermo Fisher Scientific) and a lactate assay kit (catalog# MAK064-1KT, Sigma Aldrich) following the vendor’s protocol. For the lactate assay, the supernatant was deproteinized with a 10 kDa MWCO spin filter. This additional step was needed as the presence of lactate dehydrogenase can degrade lactate and interfere with the readings. Standard curves were used to calculate the amount of glucose or lactic acid consumed by the cells daily. The data were then normalized by the number of cells.

### 4.15. Viability Assay in a Minimal Medium

After VEM treatment, cells were collected and resuspended in Biolog IF-M1 medium (catalog# 72301) supplemented with penicillin/streptomycin, 5% FBS, and 0.3 mM glutamine. The cell density was adjusted to 4 × 10^5^ cells per mL. The cell suspension was further supplemented with 4 mM glucose and/or 4 mM sodium lactate. Then, 500 µL of the cell suspension was transferred to each well of a 24-well plate and incubated at 37 °C. At t = 48 h, the cells were collected and resuspended in 500 µL of DPBS. The cells were stained with 0.25 µM SYTOX green and SYTO60 red and incubated for 15 min at room temperature. Finally, the cells were transferred to a 5 mL test tube and analyzed with a flow cytometer to measure green (excitation: 488 nm; emission: 520 nm) and red (excitation: 561 nm; emission: 586 nm) fluorescence. The live and dead cells were separated based on their fluorescence intensity, and the number of live cells were enumerated by the flow cytometer.

### 4.16. Statistical Analysis

Unequal variance *t*-test or ANOVA was used for pairwise comparisons (*p* < 0.05). For PM-M1 assays, substrates whose absorbance was found to be higher than that of the no-substrate control were selected for linear regression analysis. The slopes of untreated and treated groups for the selected substrates were compared with F statistics using GraphPad Prism 8.3.0, and the threshold of significance was set to *p* < 0.05. A minimum of three independent biological replicates were performed, and data points in figures denote the mean value ± standard error.

## Data Availability

All data have been provided in the Supplemental File.

## Supplementary Materials

The following are available online at https://www.mdpi.com/article/10.3390/metabo11110777/s1: Figure S1: Effects of VEM treatment on cell viability, morphology and growth. Figure S2: Mitoplate assays to assess the mitochondrial activities of VEM persister cells. Figures S3–S5. Phenotype microarray (PM-M1) assays to assess the metabolism of VEM persister cells. Table S1: Untargeted metabolomics data. Table S2: Metabolites that are significantly altered in persister cells.
