# Supplementary material for: BRAF-Inhibitor-Induced Metabolic Alterations in A375 Melanoma Cells
Source: Metabolites. 2021 Nov 14;11(11):777. doi: 10.3390/metabo11110777 (PMC8619236; doi:10.3390/metabo11110777)
Supplement: Supplementary file 1 [file metabolites-11-00777-s001.zip › metabolites-1467807-supplementary.pdf]

SUPPLEMENTARY MATERIALS

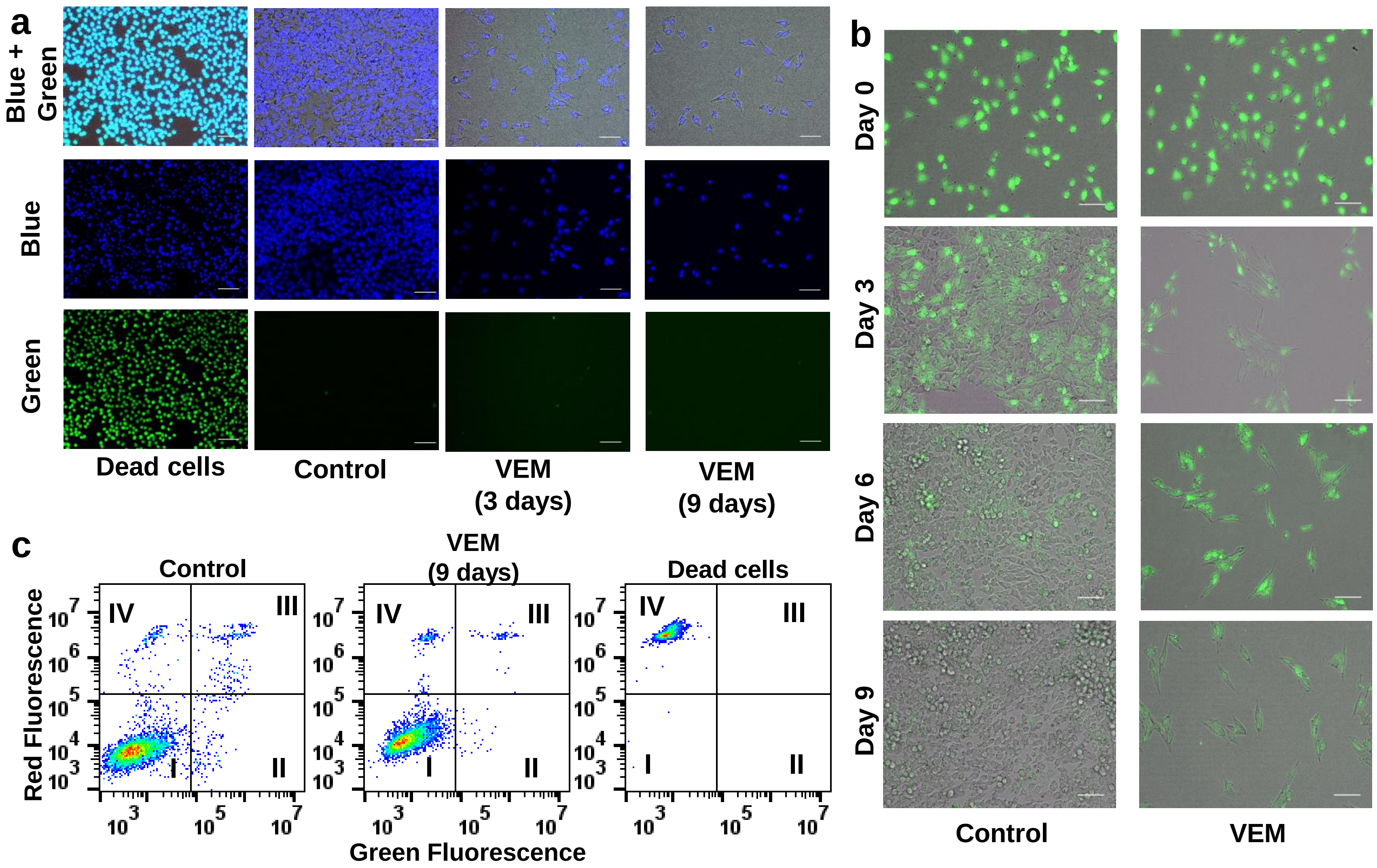

Figure S1. Effects of VEM treatment on cell viability, morphology and growth. (a) Cells surviving 3 or 9-day VEM treatment were stained with ReadyProbes Cell Viability Imaging dyes to assess live (blue) and dead (green) cells. Dead cells were generated by treating the cells with 70% ethanol for 30 min. “Control” represents the live cells that did not receive VEM treatment. **(b)** Cells pre-stained with CFSE dye were treated with VEM or left untreated (control), and their fluorescence intensity was monitored at the indicated time points with florescence microscopy. **(c)** Cells surviving 9-day VEM treatment were stained with annexin-V/FITC conjugate and PI to detect apoptotic cells. Scale bar: 100 µm.

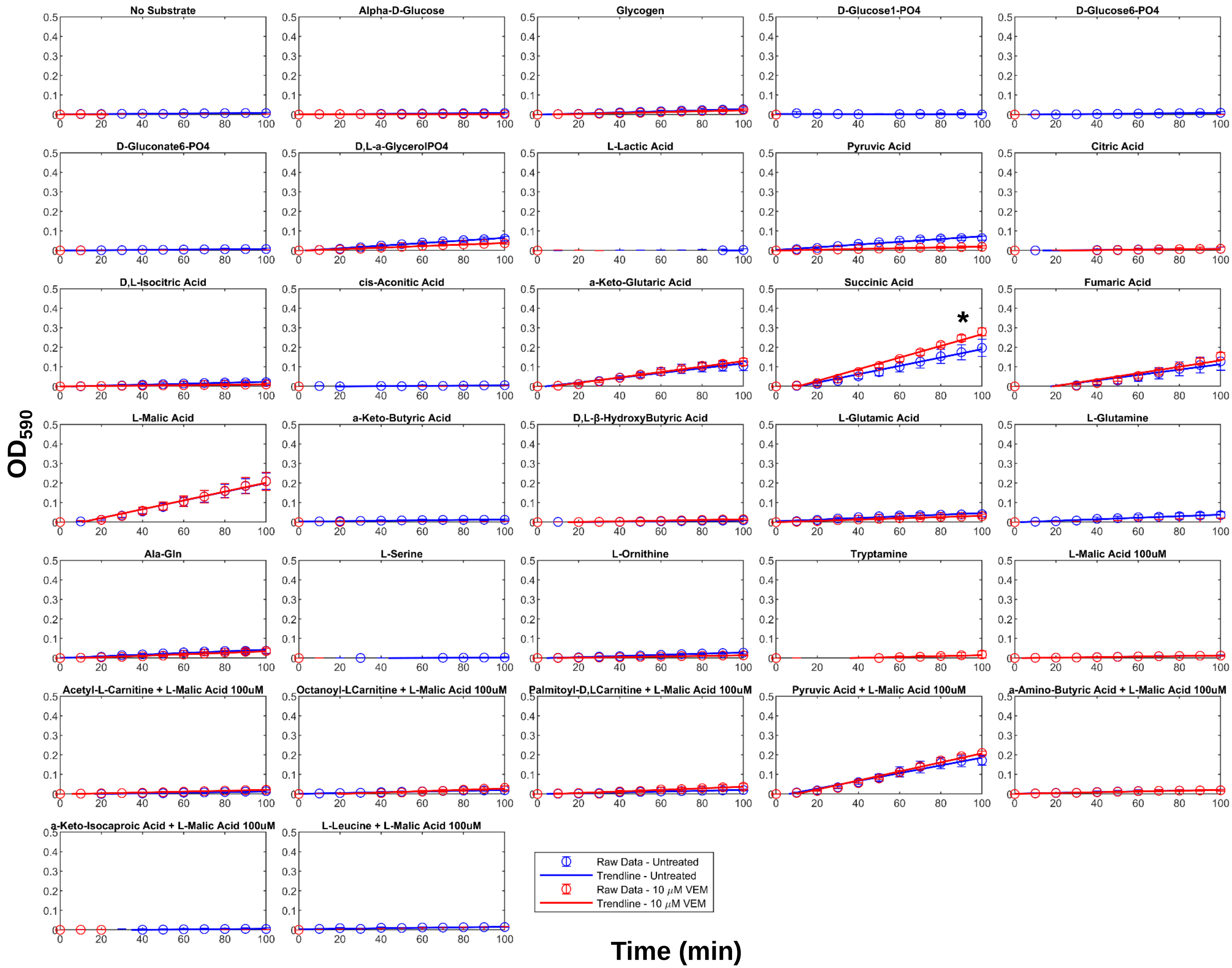

Figure S2. Mitoplate assays to assess the mitochondrial activities of VEM persister cells. The consumption rates of substrates were monitored by measuring the OD_{590} at the indicated time points. Statistical analysis was performed using a linear regression analysis (F-Statistics, *$P<0.001$). N = 4.

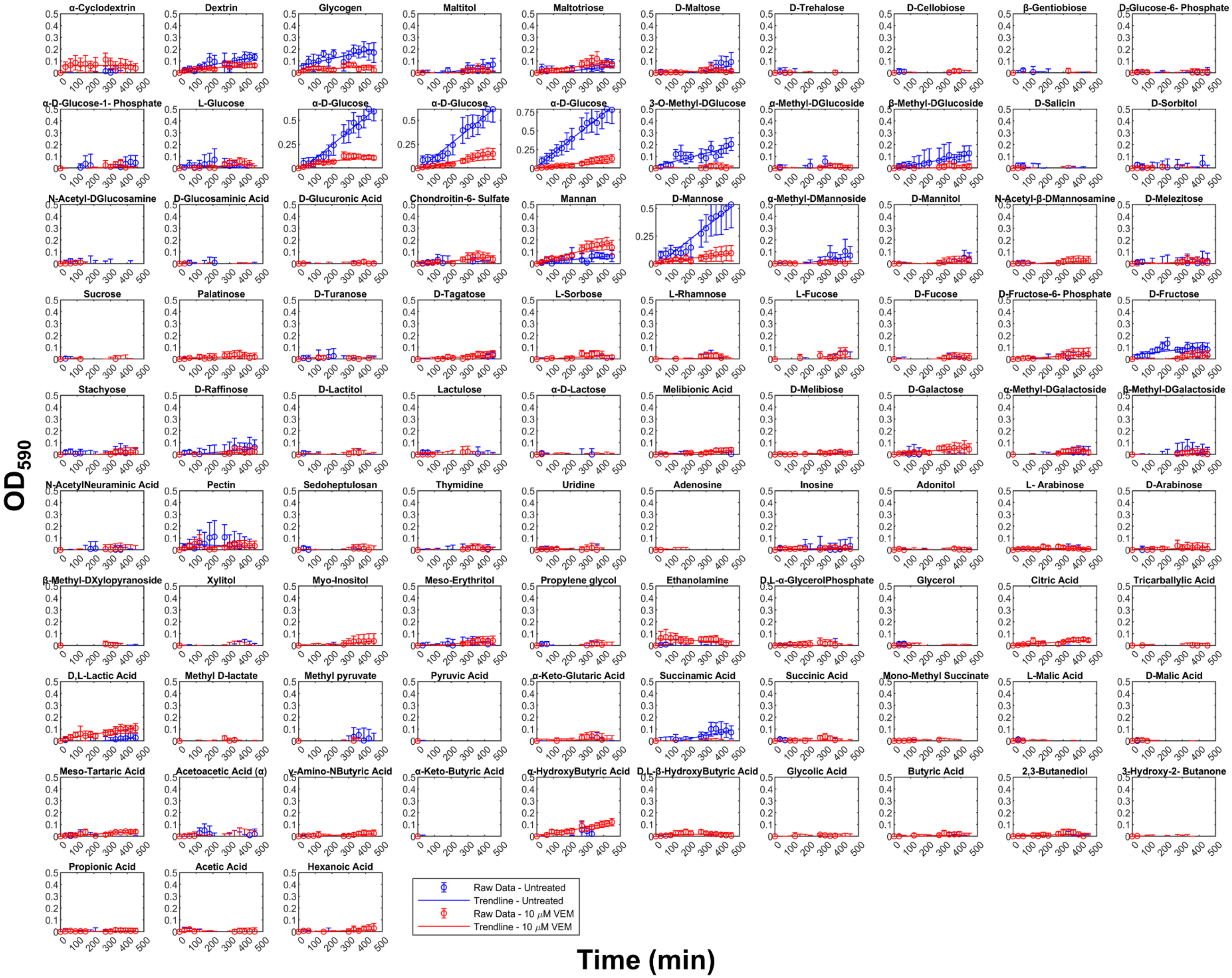

Figure S3. Phenotype microarray (PM-M1) assays to assess the metabolism of VEM persister cells. After VEM treatment, cells were transferred to PM-M1 plates with a tetrazolium dye. The consumption rates of substrates were monitored immediately by measuring absorbance (OD_{590}). The absorbance data was normalized by using t = 0 h and glutamine control data (see Materials and Methods). N=4.

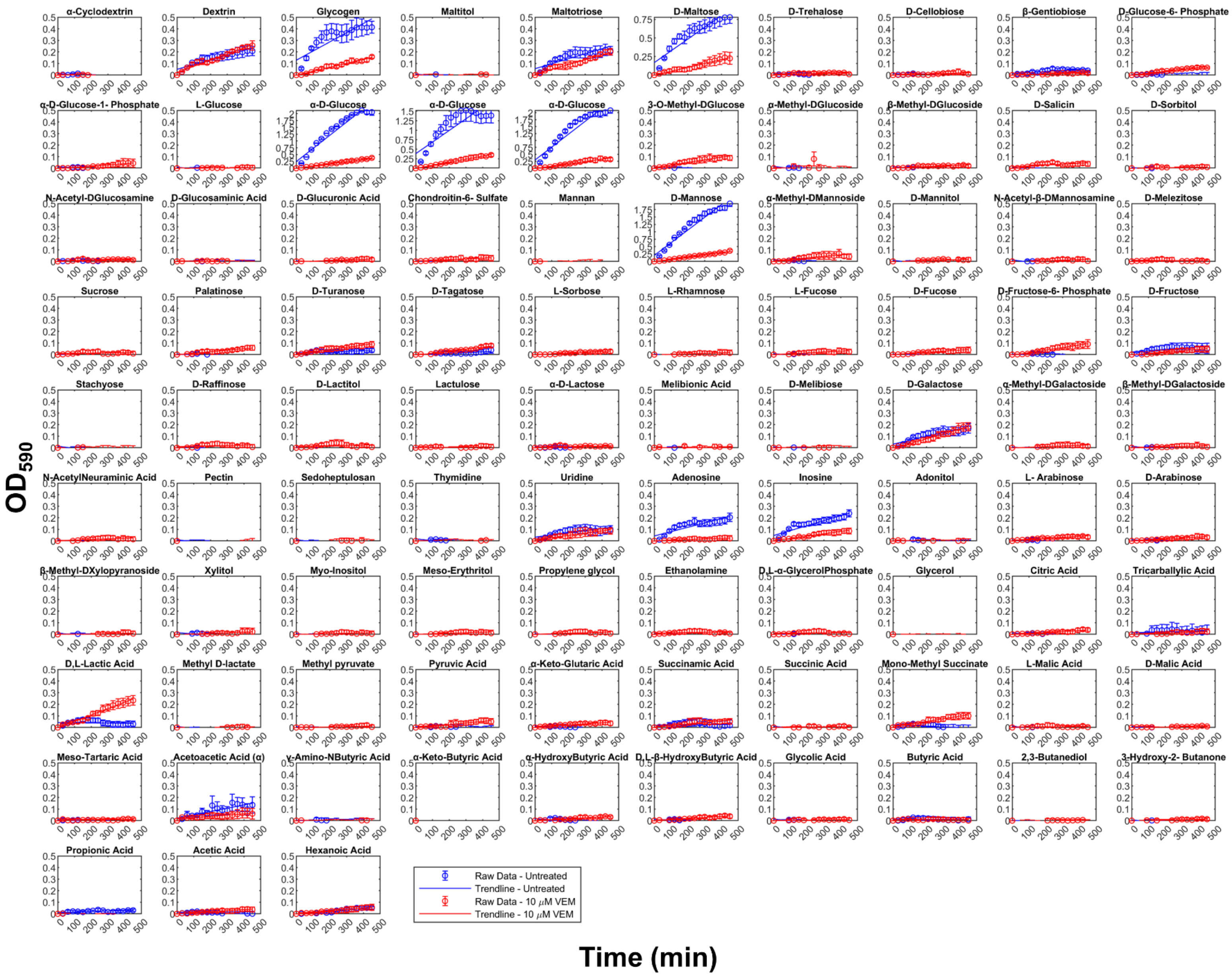

Figure S4. Phenotype microarray (PM-M1) assays to assess the metabolism of VEM persister cells. After VEM treatment, cells were transferred to PM-M1 plates. After culturing the cells for 24 h in PM-M1 plates, the tetrazolium dye was added into wells to measure the consumption rates of substrates. The absorbance data was normalized by using t = 0 h and glutamine control data (see Materials and Methods). N=4.

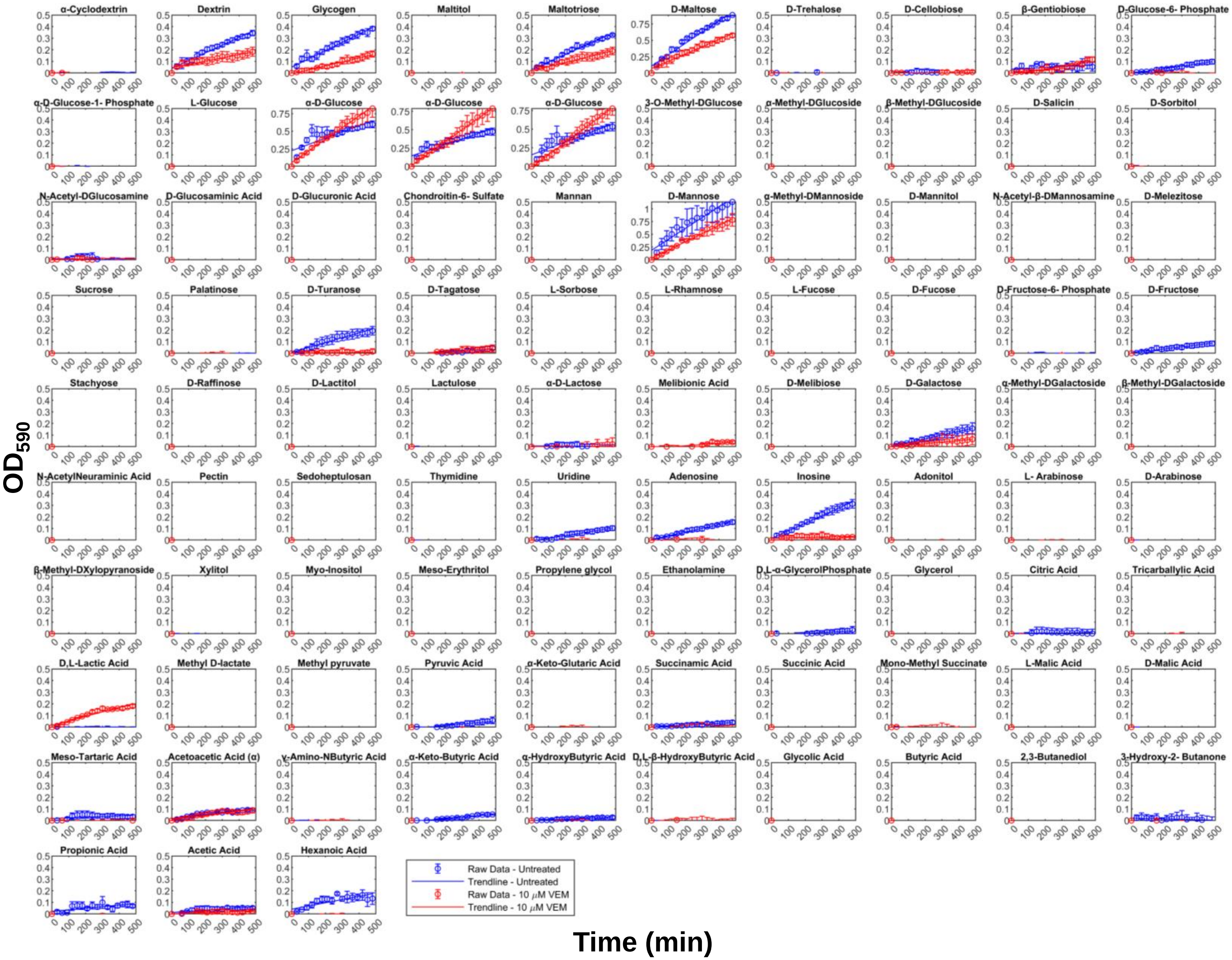

Figure S5. Phenotype microarray (PM-M1) assays to assess the metabolism of VEM persister cells. After VEM treatment, cells were transferred to PM-M1 plates. After culturing the cells for 48 h in PM-M1 plates, the tetrazolium dye was added into wells to measure the consumption rates of substrates. The absorbance data was normalized by using t = 0 h and glutamine control data (see Materials and Methods). N=4.

Table S1: Untargeted metabolomics data.

Cell Line: **A375**
Treatment: **Vemurafenib (10 µM)**
Tratement duration: **3 days**

Note: ***Values for each sample are normalized by Bradford protein concentration.***
Each biochemical in OrigScale is then rescaled to set the median equal to 1.
Lastly, missing values are imputed with the minimum.

			Replicate # 1	Replicate # 2	Replicate # 3	Replicate # 4	Replicate # 1	Replicate # 2	Replicate # 3	Replicate # 4
**BIOCHEMICAL**	**SUPER PATHWAY**	**SUB PATHWAY**	**Control**	**Control**	**Control**	**Control**	**VEM**	**VEM**	**VEM**	**VEM**
(12 or 13)-methylmyristate (a15:0 or i15:0)	Lipid	Fatty Acid, Branched	0.9872	0.6373	2.1403	0.7917	2.0035	3.4155	2.9013	1.8745
(14 or 15)-methylpalmitate (a17:0 or i17:0)	Lipid	Fatty Acid, Branched	0.8489	0.5408	2.1505	0.7658	5.9282	6.4154	4.6998	3.1366
(16 or 17)-methylstearate (a19:0 or i19:0)	Lipid	Fatty Acid, Branched	0.6929	0.4445	2.5214	0.6470	6.5427	4.7666	3.2680	3.1024
(2 or 3)-decenoate (10:1n7 or n8)	Lipid	Medium Chain Fatty Acid	1.5010	0.7514	1.3185	0.8458	1.0341	2.4317	1.8112	1.0189
(R)-3-hydroxybutyrylcarnitine	Lipid	Fatty Acid Metabolism (Acyl Carnitine, Hydroxy)	1.0283	1.1996	1.3467	1.2486	1.6371	2.0903	2.3931	1.9925
(S)-3-hydroxybutyrylcarnitine	Lipid	Fatty Acid Metabolism (Acyl Carnitine, Hydroxy)	1.0569	1.1960	1.3675	1.0657	0.8358	1.2554	1.1937	0.7911
1,2-dilinoleoyl-GPC (18:2/18:2)	Lipid	Phosphatidylcholine (PC)	0.7731	0.9270	0.7049	0.9986	2.4739	1.4221	0.8735	1.2985
1,2-dioleoyl-GPC (18:1/18:1)	Lipid	Phosphatidylcholine (PC)	1.0981	1.1041	0.9663	1.2382	2.7407	1.6758	1.2762	1.6951
1,2-dipalmitoyl-GPC (16:0/16:0)	Lipid	Phosphatidylcholine (PC)	1.1540	1.1160	0.8782	1.3578	2.6438	2.4181	1.4307	1.8010
1,2-dipalmitoyl-GPE (16:0/16:0)*	Lipid	Phosphatidylethanolamine (PE)	1.1077	1.0222	0.9922	1.0792	2.4091	1.4602	1.1247	1.3867
1-(1-enyl-oleoyl)-GPE (P-18:1)*	Lipid	Lysoplasmalogen	0.2686	0.3003	0.3224	0.3250	3.3454	1.6686	1.2137	2.0516
1-(1-enyl-palmitoyl)-2-arachidonoyl-GPC (P-16:0,	Lipid	Plasmalogen	0.3434	0.2841	0.2105	0.4890	4.6854	4.2010	3.2717	2.8155
1-(1-enyl-palmitoyl)-2-arachidonoyl-GPE (P-16:0,	Lipid	Plasmalogen	0.7925	0.7918	0.7543	0.8995	3.0192	2.3012	1.8094	1.9057
1-(1-enyl-palmitoyl)-2-linoleoyl-GPC (P-16:0/18:2	Lipid	Plasmalogen	0.9394	0.9743	0.7511	1.0617	1.8428	0.9365	0.6830	0.8669
1-(1-enyl-palmitoyl)-2-linoleoyl-GPE (P-16:0/18:2	Lipid	Plasmalogen	0.9501	0.7898	0.9314	0.9925	1.8761	1.4850	1.1739	1.1788
1-(1-enyl-palmitoyl)-2-oleoyl-GPC (P-16:0/18:1)*	Lipid	Plasmalogen	0.8620	0.8599	0.7003	1.0042	2.1446	1.2152	1.0350	1.0945
1-(1-enyl-palmitoyl)-2-oleoyl-GPE (P-16:0/18:1)*	Lipid	Plasmalogen	0.9945	0.9889	0.9960	1.0009	2.9013	1.8072	1.4996	1.6248
1-(1-enyl-palmitoyl)-2-palmitoleoyl-GPC (P-16:0/	Lipid	Plasmalogen	0.9892	0.9960	0.8380	1.0789	2.4969	1.4388	1.2407	1.3372
1-(1-enyl-palmitoyl)-2-palmitoyl-GPC (P-16:0/16:	Lipid	Plasmalogen	0.8436	0.8584	0.6985	0.9754	3.3035	2.0615	1.4972	1.7324
1-(1-enyl-palmitoyl)-GPC (P-16:0)*	Lipid	Lysoplasmalogen	0.4695	0.5102	0.4150	0.5486	1.4036	1.0257	0.6904	0.9743
1-(1-enyl-palmitoyl)-GPE (P-16:0)*	Lipid	Lysoplasmalogen	0.3075	0.3487	0.3090	0.3961	2.5075	1.4166	0.9791	1.6440
1-(1-enyl-stearoyl)-2-arachidonoyl-GPE (P-18:0/2	Lipid	Plasmalogen	0.7408	0.6999	0.6355	0.8282	4.4859	3.4686	2.6688	2.5749
1-(1-enyl-stearoyl)-2-oleoyl-GPE (P-18:0/18:1)	Lipid	Plasmalogen	0.8611	0.9735	1.1216	0.9410	3.6244	1.7637	1.2080	1.4569
1-(1-enyl-stearoyl)-GPE (P-18:0)*	Lipid	Lysoplasmalogen	0.3239	0.3446	0.3008	0.4028	2.8870	1.6746	1.2176	1.6055
1-arachidonoyl-GPE (20:4n6)*	Lipid	Lysophospholipid	0.8067	0.6973	0.6958	0.7896	2.3934	2.5016	2.3263	2.8795
1-arachidonoyl-GPI* (20:4)*	Lipid	Lysophospholipid	0.1235	0.1469	1.1646	0.3775	17.5408	29.8161	24.9516	13.2782
1-arachidonylglycerol (20:4)	Lipid	Monoacylglycerol	0.3884	0.1521	1.0000	0.2430	29.4807	28.3706	17.1630	10.8921
1-carboxyethylisoleucine	Amino Acid	Leucine, Isoleucine and Valine Metabolism	1.1564	1.2104	1.0885	1.6534	0.1557	0.1525	0.1904	0.2098
1-carboxyethylleucine	Amino Acid	Leucine, Isoleucine and Valine Metabolism	0.7366	0.8517	0.9463	1.3135	0.3352	0.3755	0.4202	0.4497
1-carboxyethylphenylalanine	Amino Acid	Phenylalanine Metabolism	0.9673	1.0590	1.0361	1.5849	0.2268	0.2426	0.2751	0.3114
1-carboxyethyltyrosine	Amino Acid	Tyrosine Metabolism	1.5648	1.6182	1.4387	2.2346	0.1389	0.1208	0.1554	0.1771
1-carboxyethylvaline	Amino Acid	Leucine, Isoleucine and Valine Metabolism	1.2682	1.3854	1.1128	1.9068	0.1580	0.1744	0.2171	0.2524
1-dihomo-linolenylglycerol (20:3)	Lipid	Monoacylglycerol	0.8102	0.4879	2.2528	0.6453	21.9995	42.5190	22.9848	21.6596
1-docosahexaenoylglycerol (22:6)	Lipid	Monoacylglycerol	0.6993	0.3564	2.0420	0.5157	28.1985	25.1518	15.1269	10.4753
1-heptadecenoylglycerol (17:1)*	Lipid	Monoacylglycerol	0.9146	0.5832	3.6879	0.8155	9.9348	14.2252	9.0517	4.3745
1-lignoceroyl-GPC (24:0)	Lipid	Lysophospholipid	0.7718	0.9000	0.8287	1.1046	1.6389	0.8833	0.5988	0.9393
1-linoleoyl-GPE (18:2)*	Lipid	Lysophospholipid	0.7492	0.6766	0.8881	0.8367	1.3112	1.0109	1.2568	1.4848
1-linoleoylglycerol (18:2)	Lipid	Monoacylglycerol	0.6498	0.5197	2.6574	0.9443	16.2955	18.8887	12.7364	8.2140
1-margaroylglycerol (17:0)	Lipid	Monoacylglycerol	0.4692	0.5831	2.0795	0.4023	4.8206	4.1332	4.1162	2.9785
1-methyl-4-imidazoleacetate	Amino Acid	Histidine Metabolism	1.6635	1.5544	1.5630	1.7651	1.1863	1.6256	1.2036	0.9010
1-methyl-5-imidazoleacetate	Amino Acid	Histidine Metabolism	5.5341	4.4177	4.2957	5.0801	0.4599	0.5563	0.4016	0.3609
1-methylguanidine	Amino Acid	Guanidino and Acetamido Metabolism	0.8985	0.9799	1.0214	1.2185	0.9879	0.9727	0.9383	1.2309
1-methylhistidine	Amino Acid	Histidine Metabolism	0.6743	0.7213	0.7805	1.5792	1.2013	0.9130	1.0199	1.1993
1-methylnicotinamide	Cofactors and Vitami	Nicotinate and Nicotinamide Metabolism	0.7236	0.6915	0.5317	0.8850	1.6263	1.3549	1.0247	1.6110
1-myristoyl-2-arachidonoyl-GPC (14:0/20:4)*	Lipid	Phosphatidylcholine (PC)	0.3564	0.4303	0.3418	0.5106	2.6500	2.1316	1.1673	1.6319
1-myristoyl-2-palmitoyl-GPC (14:0/16:0)	Lipid	Phosphatidylcholine (PC)	1.1105	1.0969	0.9403	1.2043	2.1904	1.7664	1.1980	1.4121
1-myristoylglycerol (14:0)	Lipid	Monoacylglycerol	0.8811	0.5730	2.7784	0.6983	6.7842	7.8367	4.9627	3.5875
1-oleoyl-2-arachidonoyl-GPE (18:1/20:4)*	Lipid	Phosphatidylethanolamine (PE)	0.6001	0.7539	0.5042	0.8803	3.5000	2.8599	1.7693	2.2317
1-oleoyl-2-arachidonoyl-GPI (18:1/20:4)*	Lipid	Phosphatidylinositol (PI)	0.6098	0.7437	0.6006	0.9511	3.7176	2.6361	1.1153	1.7665
1-oleoyl-2-docosahexaenoyl-GPC (18:1/22:6)*	Lipid	Phosphatidylcholine (PC)	0.5149	0.6095	0.4375	0.6757	2.5936	1.4137	1.0574	1.4396
1-oleoyl-2-docosahexaenoyl-GPE (18:1/22:6)*	Lipid	Phosphatidylethanolamine (PE)	0.6869	0.4700	0.5379	0.8352	4.8383	3.8884	3.0957	3.3158
1-oleoyl-2-linoleoyl-GPE (18:1/18:2)*	Lipid	Phosphatidylethanolamine (PE)	0.9677	0.9744	0.9043	1.0226	1.7094	1.2622	0.9419	1.1500
1-oleoyl-GPC (18:1)	Lipid	Lysophospholipid	0.8046	0.7820	0.9529	0.7526	1.5548	1.0198	0.9802	1.6438
1-oleoyl-GPE (18:1)	Lipid	Lysophospholipid	0.4885	0.5174	0.6302	0.6999	1.6256	1.2535	1.3794	1.6850
1-oleoyl-GPG (18:1)*	Lipid	Lysophospholipid	0.8773	0.7092	1.0396	0.8156	1.2608	0.7579	1.1984	1.0173
1-oleoyl-GPI (18:1)	Lipid	Lysophospholipid	0.4041	0.1251	0.1708	0.6639	4.9774	2.9408	3.2542	1.9471
1-oleoyl-GPS (18:1)	Lipid	Lysophospholipid	0.3775	0.2736	1.8503	0.4640	2.0329	3.9376	3.0635	2.5320
1-oleoylglycerol (18:1)	Lipid	Monoacylglycerol	0.8349	0.6859	4.5064	0.6786	14.5853	13.0036	10.9019	8.2433
1-palmitoleoyl-2-linolenoyl-GPC (16:1/18:3)*	Lipid	Phosphatidylcholine (PC)	1.1190	1.4501	0.9091	1.4257	0.2179	0.2179	0.2179	0.2179
1-palmitoleoyl-GPC* (16:1)*	Lipid	Lysophospholipid	0.9794	0.9421	1.1222	0.6810	1.0748	0.9842	0.9130	1.0158
1-palmitoleoylglycerol (16:1)*	Lipid	Monoacylglycerol	0.9502	0.4701	2.6769	0.5898	7.6073	6.8906	4.4148	3.2895
1-palmitoyl-2-arachidonoyl-GPC (16:0/20:4n6)	Lipid	Phosphatidylcholine (PC)	0.3541	0.4207	0.3010	0.5600	3.4534	2.6834	1.5939	1.9498
1-palmitoyl-2-arachidonoyl-GPE (16:0/20:4)*	Lipid	Phosphatidylethanolamine (PE)	0.8372	0.8842	0.6429	1.0496	2.2884	1.9071	1.1561	1.4794
1-palmitoyl-2-arachidonoyl-GPI (16:0/20:4)*	Lipid	Phosphatidylinositol (PI)	0.7026	0.6060	0.5503	0.9312	2.2178	1.3390	0.6403	1.0117
1-palmitoyl-2-docosahexaenoyl-GPC (16:0/22:6)	Lipid	Phosphatidylcholine (PC)	0.3986	0.4776	0.3243	0.6023	2.0035	1.4031	0.8148	1.1538
1-palmitoyl-2-docosahexaenoyl-GPE (16:0/22:6)	Lipid	Phosphatidylethanolamine (PE)	0.6028	0.5562	0.3517	0.7526	2.5919	2.3663	1.5812	1.8925
1-palmitoyl-2-oleoyl-GPC (16:0/18:1)	Lipid	Phosphatidylcholine (PC)	0.9802	0.9440	0.8017	1.1250	2.2457	1.8470	1.4097	1.4980
1-palmitoyl-2-oleoyl-GPE (16:0/18:1)	Lipid	Phosphatidylethanolamine (PE)	1.1573	1.1737	1.0216	1.2125	1.7209	1.1188	0.8271	1.0479
1-palmitoyl-2-oleoyl-GPG (16:0/18:1)	Lipid	Phosphatidylglycerol (PG)	0.8060	0.8530	0.7874	0.9962	3.9619	1.6982	1.0414	1.2501
1-palmitoyl-2-oleoyl-GPI (16:0/18:1)*	Lipid	Phosphatidylinositol (PI)	1.0340	0.9681	0.6571	1.2276	1.7240	1.4340	1.3080	1.5110
1-palmitoyl-2-oleoyl-GPS (16:0/18:1)	Lipid	Phosphatidylserine (PS)	1.4634	1.6037	1.2171	1.5434	1.9063	1.2772	0.7795	1.0692
1-palmitoyl-2-palmitoleoyl-GPC (16:0/16:1)*	Lipid	Phosphatidylcholine (PC)	1.1069	1.0976	0.9013	1.2345	2.1468	1.5768	1.1655	1.3879
1-palmitoyl-2-stearoyl-GPC (16:0/18:0)	Lipid	Phosphatidylcholine (PC)	1.0007	1.0906	0.8646	1.0956	1.9458	1.4502	0.8854	1.0196
1-palmitoyl-GPC (16:0)	Lipid	Lysophospholipid	0.9335	0.8238	0.8944	0.8545	1.3941	1.0536	0.9774	1.4083
1-palmitoyl-GPE (16:0)	Lipid	Lysophospholipid	0.7007	0.7285	0.5739	0.8735	2.2029	0.9736	0.6968	1.3391
1-palmitoyl-GPG (16:0)*	Lipid	Lysophospholipid	1.0526	0.6296	3.4308	0.8493	1.7894	2.3114	2.0670	0.6903
1-palmitoyl-GPI* (16:0)	Lipid	Lysophospholipid	0.2179	0.1758	1.0464	0.3717	4.7409	9.7287	5.0775	3.2304
1-palmitoyl-GPS (16:0)*	Lipid	Lysophospholipid	0.6839	0.3808	3.4393	1.1401	0.2167	0.2167	0.9807	0.8505
1-palmitoylglycerol (16:0)	Lipid	Monoacylglycerol	1.1083	0.8917	3.2457	0.8603	3.8770	7.0260	4.7120	3.6888
1-pentadecanoylglycerol (15:0)	Lipid	Monoacylglycerol	0.8087	0.5401	1.6650	0.6828	5.2804	5.8627	3.8750	2.7799
1-ribosyl-imidazoleacetate*	Amino Acid	Histidine Metabolism	1.7135	1.6602	1.9586	1.8680	0.9047	1.0000	0.6573	0.4197
1-stearoyl-2-arachidonoyl-GPC (18:0/20:4)	Lipid	Phosphatidylcholine (PC)	0.3469	0.3986	0.2919	0.5296	5.7498	4.9142	3.0871	3.4820
1-stearoyl-2-arachidonoyl-GPE (18:0/20:4)	Lipid	Phosphatidylethanolamine (PE)	0.8128	0.8637	0.7190	1.0093	3.6940	3.0643	2.2173	2.3761
1-stearoyl-2-arachidonoyl-GPI (18:0/20:4)	Lipid	Phosphatidylinositol (PI)	0.9796	0.7785	0.7107	0.9088	5.5927	3.1424	2.0567	2.0064
1-stearoyl-2-arachidonoyl-GPS (18:0/20:4)	Lipid	Phosphatidylserine (PS)	0.7819	0.9437	0.5854	0.9709	3.4459	2.9971	1.6757	1.7231
1-stearoyl-2-docosahexaenoyl-GPC (18:0/22:6)	Lipid	Phosphatidylcholine (PC)	0.3976	0.4312	0.3090	0.6203	3.2575	2.5354	1.6735	2.0783
1-stearoyl-2-linoleoyl-GPC (18:0/18:2)*	Lipid	Phosphatidylcholine (PC)	0.9222	0.9739	0.9819	1.0821	2.2526	2.3737	2.3084	1.6391
1-stearoyl-2-oleoyl-GPC (18:0/18:1)	Lipid	Phosphatidylcholine (PC)	0.8880	0.9658	0.8406	0.9856	2.8819	1.9726	1.5329	1.6641
1-stearoyl-2-oleoyl-GPE (18:0/18:1)	Lipid	Phosphatidylethanolamine (PE)	0.9634	0.9173	0.8019	1.0573	2.7230	1.9468	1.3396	1.5699
1-stearoyl-2-oleoyl-GPI (18:0/18:1)*	Lipid	Phosphatidylinositol (PI)	1.1703	1.0354	0.5894	1.2724	4.0481	1.1989	0.4370	1.1648
1-stearoyl-2-oleoyl-GPS (18:0/18:1)	Lipid	Phosphatidylserine (PS)	1.1052	1.1167	0.9139	1.1873	3.5385	2.5859	1.5509	1.8307
1-stearoyl-GPC (18:0)	Lipid	Lysophospholipid	0.6101	0.6750	0.5392	0.6686	3.4293	2.1935	1.5172	2.3930
1-stearoyl-GPE (18:0)	Lipid	Lysophospholipid	0.5174	0.5723	0.4267	0.7593	3.0415	1.7886	1.3630	1.7684
1-stearoyl-GPG (18:0)	Lipid	Lysophospholipid	0.9157	0.4397	3.6001	0.6855	0.2210	2.0431	1.2547	0.2210
1-stearoyl-GPI (18:0)	Lipid	Lysophospholipid	0.3466	0.3008	1.7165	0.6021	2.9260	6.5776	5.7305	2.7609
1-stearoyl-GPS (18:0)*	Lipid	Lysophospholipid	0.5572	0.6982	0.4159	0.9878	2.1037	1.1966	0.6025	1.0940
10-heptadecenoate (17:1n7)	Lipid	Long Chain Monounsaturated Fatty Acid	0.4410	0.2628	1.2195	0.4505	6.2048	6.5142	4.8411	2.4758
10-nonadecenoate (19:1n9)	Lipid	Long Chain Monounsaturated Fatty Acid	0.4550	0.2803	1.5895	0.4665	7.8206	7.0987	5.3473	3.8361
2'-deoxyadenosine	Nucleotide	Purine Metabolism, Adenine containing	1.1047	1.2011	1.0000	1.9532	1.2567	0.3156	0.5864	0.3156
2'-deoxyadenosine 5'-diphosphate	Nucleotide	Purine Metabolism, Adenine containing	1.5356	1.2201	0.7799	0.5953	0.5953	0.5953	0.5953	0.5953
2'-deoxyadenosine 5'-monophosphate	Nucleotide	Purine Metabolism, Adenine containing	19.9261	19.7274	11.5798	17.2571	0.5537	0.5537	0.5537	0.5537
2'-deoxycytidine	Nucleotide	Pyrimidine Metabolism, Cytidine containing	1.2078	2.0902	3.6156	4.7450	0.7922	0.5739	0.6575	0.7474

dCMP	Nucleotide	Pyrimidine Metabolism, Cytidine containing	2.1061	2.1812	1.7576	1.5396	0.3606	0.3073	0.3304	0.4158
2'-deoxyguanosine	Nucleotide	Purine Metabolism, Guanine containing	2.5268	2.9952	2.9637	3.8479	2.5717	1.2340	0.8986	1.1602
2'-deoxyinosine	Nucleotide	Purine Metabolism, (Hypo)Xanthine/Inosine containing	5.7718	7.3468	5.0753	12.3254	2.6425	0.9614	0.8393	1.2136
2'-deoxyuridine	Nucleotide	Pyrimidine Metabolism, Uracil containing	0.2312	0.2068	0.1311	0.4746	1.5254	1.5457	2.4033	2.6309
2'-O-methylcytidine	Nucleotide	Pyrimidine Metabolism, Cytidine containing	0.5675	0.4122	0.5219	0.7028	3.3179	3.6107	3.8081	3.3485
2'-O-methyluridine	Nucleotide	Pyrimidine Metabolism, Uracil containing	1.1596	0.7290	0.7278	0.7904	1.9525	1.3511	1.7084	1.5856
2,3-dihydroxy-2-methylbutyrate	Amino Acid	Leucine, Isoleucine and Valine Metabolism	2.0518	1.8503	1.4280	2.1823	0.6437	0.5185	0.7604	0.6771
2,3-dihydroxy-5-methylthio-4-pentenoate (DMT	Amino Acid	Methionine, Cysteine, SAM and Taurine Metabolism	1.0000	0.9443	1.0370	1.0596	0.5769	0.7504	0.5769	0.5769
2,4-di-tert-butylphenol	Xenobiotics	Chemical	0.4679	0.4541	3.9864	0.5762	1.8904	2.5566	1.6842	0.9211
2,4-dihydroxybutyrate	Lipid	Fatty Acid, Dihydroxy	1.1263	0.9791	0.9501	1.5699	0.3641	0.5081	0.4586	0.4621
2-aminoadipate	Amino Acid	Lysine Metabolism	0.5295	0.4812	0.3698	0.7172	1.1943	0.8205	1.9074	0.8906
2-aminophenol sulfate	Xenobiotics	Food Component/Plant	1.6772	1.2213	1.6050	0.9073	0.3812	0.3812	0.3812	0.3812
2-arachidonoylglycerol (20:4)	Lipid	Monoacylglycerol	0.4169	0.0864	1.2132	0.2720	26.9431	21.7098	11.1317	10.0085
2-docosahexaenoylglycerol (22:6)*	Lipid	Monoacylglycerol	0.3370	0.2588	1.6058	0.2700	19.2641	11.4239	6.5059	7.2151
2-heptadecenoylglycerol (17:1)*	Lipid	Monoacylglycerol	0.4782	0.3448	2.0208	0.5269	8.6654	8.6237	5.3742	4.1417
2-hydroxy-3-methylvalerate	Amino Acid	Leucine, Isoleucine and Valine Metabolism	1.5176	1.1565	0.8963	1.1661	0.4538	0.4538	0.4538	0.4538
2-hydroxyadipate	Lipid	Fatty Acid, Dicarboxylate	1.0000	0.3681	0.2657	0.9913	0.4074	0.2657	0.5682	0.4774
2-hydroxybutyrate/2-hydroxyisobutyrate	Amino Acid	Glutathione Metabolism	1.2161	0.9911	1.1309	1.0015	0.7957	0.7957	0.7957	0.7957
2-hydroxyglutarate	Lipid	Fatty Acid, Dicarboxylate	1.7031	1.0844	1.3360	1.7343	0.8532	0.8605	0.9156	0.5723
2-hydroxypalmitate	Lipid	Fatty Acid, Monohydroxy	0.8915	0.5664	2.2120	0.6759	3.5607	3.8700	2.5171	2.4735
2-hydroxystearate	Lipid	Fatty Acid, Monohydroxy	0.7579	0.5723	2.1565	0.7677	2.8325	4.6824	3.3692	3.0452
2-linoleoylglycerol (18:2)	Lipid	Monoacylglycerol	0.5040	0.2970	1.9601	0.4415	14.4387	15.9136	8.4740	7.4274
2-methylbutyrylcarnitine (C5)	Amino Acid	Leucine, Isoleucine and Valine Metabolism	1.0123	0.9239	1.1245	0.9434	1.6500	1.5512	1.1629	1.7910
2-methylbutyrylglycine (C5)	Amino Acid	Leucine, Isoleucine and Valine Metabolism	1.5697	0.9724	1.4368	0.9628	0.7010	1.5938	0.7010	0.7010
2-methylcitrate/homocitrate	Energy	TCA Cycle	0.7437	0.6362	0.8264	1.0430	2.2162	2.2817	1.9204	2.2256
2-myristoylglycerol (14:0)	Lipid	Monoacylglycerol	0.9660	0.4448	2.4451	0.6064	5.4644	6.3622	3.7620	3.2396
2-O-methylascorbic acid	Cofactors and Vitami	Ascorbate and Aldarate Metabolism	0.9285	0.8302	0.8445	0.9710	1.8284	1.6664	1.5526	1.6379
2-oleoylglycerol (18:1)	Lipid	Monoacylglycerol	0.7761	0.5266	4.5149	0.6114	9.8128	13.7737	8.2068	9.7065
2-oxoarginine*	Amino Acid	Urea cycle; Arginine and Proline Metabolism	0.9094	0.9901	0.6939	1.0011	0.9989	1.3314	1.1601	1.0124
2-palmitoleoyl-GPC* (16:1)*	Lipid	Lysophospholipid	1.0048	1.0368	1.1819	1.0019	0.9505	0.9837	0.7397	1.1346
2-palmitoleoylglycerol (16:1)*	Lipid	Monoacylglycerol	0.7090	0.3732	2.8061	0.4324	7.2502	6.7039	3.9828	4.2001
2-palmitoyl-GPC* (16:0)*	Lipid	Lysophospholipid	0.6081	0.4435	2.4994	0.5472	1.6233	3.4881	2.1880	1.6269
2-palmitoylglycerol (16:0)	Lipid	Monoacylglycerol	0.9291	0.6408	2.9088	0.7818	0.1209	9.2692	5.2674	4.5845
2-stearoyl-GPE (18:0)*	Lipid	Lysophospholipid	0.4014	0.5156	2.3316	0.8965	1.8857	6.3449	3.9840	1.8860
2R,3R-dihydroxybutyrate	Lipid	Fatty Acid, Dihydroxy	1.5266	1.3386	1.0149	1.3231	0.8315	0.9851	0.4512	0.9274
2S,3R-dihydroxybutyrate	Lipid	Fatty Acid, Dihydroxy	1.3224	0.5285	0.5285	1.9720	0.5285	0.5285	0.5285	0.8857
3'-dephosphocoenzyme A	Cofactors and Vitami	Pantothenate and CoA Metabolism	0.4325	0.2690	0.5376	0.9654	0.8890	0.7385	1.0346	1.6224
3-(3-amino-3-carboxypropyl)uridine*	Nucleotide	Pyrimidine Metabolism, Uracil containing	1.0831	1.0439	1.2818	1.1657	1.0489	0.8006	0.7832	0.9296
3-(4-hydroxyphenyl)lactate (HPLA)	Amino Acid	Tyrosine Metabolism	1.3108	1.1546	1.1151	1.4830	0.3608	0.3190	0.3863	0.3772
3-amino-2-piperidone	Amino Acid	Urea cycle; Arginine and Proline Metabolism	0.3002	0.2911	0.2303	0.7418	2.1184	1.6728	0.9961	1.5840
3-aminoisobutyrate	Nucleotide	Pyrimidine Metabolism, Thymine containing	0.9345	0.6166	1.2740	0.6812	1.9701	2.2721	1.8488	1.9929
3-formylindole	Xenobiotics	Food Component/Plant	0.5614	0.5223	0.9174	1.0749	1.2705	0.9927	1.4405	1.2361
3-hydroxy-2-ethylpropionate	Amino Acid	Leucine, Isoleucine and Valine Metabolism	1.6832	0.7540	0.7540	0.8761	0.8596	0.7540	0.7540	1.6687
3-hydroxy-3-methylglutarate	Lipid	Mevalonate Metabolism	3.3635	3.3011	3.4380	2.8242	0.7138	0.7306	0.6726	0.7042
3-hydroxyadipate*	Lipid	Fatty Acid, Dicarboxylate	1.0428	0.3764	0.6139	1.0779	1.0210	1.1020	0.9847	0.8774
3-hydroxybutyrate (BHBA)	Lipid	Ketone Bodies	1.1843	0.9548	0.9181	1.0336	1.5645	1.6042	2.1352	1.1493
3-hydroxydecanoate	Lipid	Fatty Acid, Monohydroxy	0.5520	0.6477	1.2639	0.8909	1.0263	0.8457	1.5944	0.5826
3-hydroxydecanoylcarnitine	Lipid	Fatty Acid Metabolism (Acyl Carnitine, Hydroxy)	0.3172	0.5344	1.1678	0.6107	1.1364	1.1163	1.4833	0.8593
3-hydroxyhexanoate	Lipid	Fatty Acid, Monohydroxy	0.6373	0.7884	1.7539	0.9975	1.0025	0.8666	1.8741	0.4610
3-hydroxyhexanoylcarnitine (1)	Lipid	Fatty Acid Metabolism (Acyl Carnitine, Hydroxy)	0.5858	0.6943	0.9914	0.9667	2.3830	2.9522	2.7891	2.9184
3-hydroxyhippurate	Xenobiotics	Benzoate Metabolism	0.5935	0.5935	0.5935	0.5935	2.6507	3.8589	0.5935	1.7323
3-hydroxyisobutyrate	Amino Acid	Leucine, Isoleucine and Valine Metabolism	0.9392	0.8078	0.6600	1.1961	0.8384	0.9451	0.6131	1.0549
3-hydroxylaurate	Lipid	Fatty Acid, Monohydroxy	0.6992	0.6644	1.3339	0.9391	1.0114	0.8036	1.4914	0.7121
3-hydroxymyristate	Lipid	Fatty Acid, Monohydroxy	1.0669	0.6018	1.8181	0.8131	1.8846	1.7954	1.9655	0.8690
3-hydroxyoctanoate	Lipid	Fatty Acid, Monohydroxy	0.5141	0.6129	1.6960	0.9371	0.9120	0.8028	1.6274	0.4622
3-hydroxyoleate*	Lipid	Fatty Acid, Monohydroxy	0.7839	0.3596	1.9096	0.5405	5.3042	4.3858	3.1736	1.9023
3-hydroxyoleoylcarnitine	Lipid	Fatty Acid Metabolism (Acyl Carnitine, Hydroxy)	0.6447	0.6885	0.9387	0.9810	1.5732	1.0190	0.5919	1.0588
3-hydroxypalmitate	Lipid	Fatty Acid, Monohydroxy	1.0220	0.5292	2.4336	0.7386	3.7982	2.4854	3.1278	0.9780
3-hydroxypalmitoylcarnitine	Lipid	Fatty Acid Metabolism (Acyl Carnitine, Hydroxy)	0.6729	1.0579	1.6510	1.1609	0.9421	0.6949	0.5210	0.6079
3-hydroxystearate	Lipid	Fatty Acid, Monohydroxy	0.7620	0.5893	2.5248	0.8143	1.9910	2.9248	3.3408	1.0884
3-hydroxytridecanoate	Lipid	Fatty Acid, Monohydroxy	0.9920	0.7157	1.8275	0.8672	3.3885	3.6771	2.6737	1.7588
3-ketosphinganine	Lipid	Sphingolipid Synthesis	0.4620	0.5059	0.4166	0.4166	0.4166	0.4166	0.4166	0.4166
3-methoxytyrosine	Amino Acid	Tyrosine Metabolism	1.0126	0.4488	0.6368	0.9874	0.4488	0.4488	0.6834	0.5346
3-methyl-2-oxobutyrate	Amino Acid	Leucine, Isoleucine and Valine Metabolism	2.4941	2.2825	3.1301	0.9438	0.8037	1.0292	1.3066	0.8037
3-methyl-2-oxovalerate	Amino Acid	Leucine, Isoleucine and Valine Metabolism	2.1442	2.6178	3.1915	0.7993	0.7470	1.2053	0.9025	0.7470
3-methylcytidine	Nucleotide	Pyrimidine Metabolism, Cytidine containing	0.3583	0.3638	0.5305	0.5751	2.0983	2.2288	1.9744	2.1460
3-methylglutaconate	Amino Acid	Leucine, Isoleucine and Valine Metabolism	1.0087	0.8351	1.0548	0.8895	0.9913	1.0542	0.8760	1.1653
3-methylhistidine	Amino Acid	Histidine Metabolism	0.7012	0.7986	0.7769	0.8550	0.1660	0.4881	0.2748	0.3230
3-phosphoglycerate	Carbohydrate	Glycolysis, Gluconeogenesis, and Pyruvate Metabolism	1.4278	0.7555	0.8553	1.5989	0.6773	0.5634	1.4911	1.4691
3-ureidopropionate	Nucleotide	Pyrimidine Metabolism, Uracil containing	2.5390	1.8100	2.0242	1.9842	0.9376	1.2218	1.0126	0.9112
4-acetamidobutanoate	Amino Acid	Polyamine Metabolism	1.1438	1.0167	0.9833	1.0904	1.6885	1.6125	1.5244	1.3215
4-chlorobenzoic acid	Xenobiotics	Chemical	0.7798	1.0221	1.0131	0.7229	3.1486	1.5793	2.2814	0.7229
4-cholesten-3-one	Lipid	Sterol	0.7594	0.6365	0.6365	0.9270	2.7598	0.6365	0.8407	0.6365
4-guanidinobutanoate	Amino Acid	Guanidino and Acetamido Metabolism	2.6800	2.4216	2.6050	2.4652	0.6044	0.7115	0.7069	0.7164
4-hydroxy-nonenal-glutathione	Amino Acid	Glutathione Metabolism	1.6230	0.9610	0.9192	1.3324	3.2859	2.4351	2.1180	2.3419
4-hydroxybutyrate (GHB)	Lipid	Fatty Acid, Monohydroxy	0.8246	0.7035	0.7976	1.0043	0.8321	0.6521	0.6521	0.6521
4-hydroxyglutamate	Amino Acid	Glutamate Metabolism	3.3340	3.2436	3.0458	3.2680	0.7827	0.7827	0.7827	0.7827
4-hydroxyphenylpyruvate	Amino Acid	Tyrosine Metabolism	0.8218	0.8179	0.6291	0.9948	1.9259	1.3622	1.9811	2.0488
4-imidazoleacetate	Amino Acid	Histidine Metabolism	2.4020	1.9383	1.6982	2.0930	0.7281	0.4980	0.6290	0.3443
4-methyl-2-oxopentanoate	Amino Acid	Leucine, Isoleucine and Valine Metabolism	2.0700	1.9009	2.6749	1.7823	0.5982	1.3052	1.1251	0.9202
4-methylcatechol sulfate	Xenobiotics	Benzoate Metabolism	0.5501	0.5458	0.5584	0.8402	1.7192	2.3615	2.3209	1.6896
5,6-dihydrothymine	Nucleotide	Pyrimidine Metabolism, Thymine containing	1.0663	0.9064	0.9556	1.0444	0.9064	0.9064	0.9064	0.9064
5,6-dihydrouridine	Nucleotide	Pyrimidine Metabolism, Uracil containing	0.5755	0.5808	0.6808	0.7020	1.4603	1.5604	1.5183	1.8522
5-(2-Hydroxyethyl)-4-methylthiazole	Cofactors and Vitami	Thiamine Metabolism	0.2902	0.2935	0.3336	0.4953	1.0007	1.2443	1.3205	1.4474
5-(galactosylhydroxy)-L-lysine	Amino Acid	Lysine Metabolism	0.3670	0.3431	0.4088	0.4634	1.7212	1.7427	1.2454	1.9299
5-aminovalerate	Amino Acid	Lysine Metabolism	1.5189	1.4575	1.8022	1.4142	0.4591	0.7022	0.6140	0.6186
5-dodecenoate (12:1n7)	Lipid	Medium Chain Fatty Acid	0.9560	0.8782	1.8899	1.0440	0.7292	0.7769	1.0647	0.4882
5-dodecenoylcarnitine (C12:1)	Lipid	Fatty Acid Metabolism (Acyl Carnitine, Monounsaturated)	1.2181	1.3585	2.1509	0.8334	0.6478	1.3671	1.0165	0.8391
5-hydroxylysine	Amino Acid	Lysine Metabolism	0.4087	0.6067	0.8819	0.4915	1.5217	1.8320	0.9508	1.2857
5-methylcytidine	Nucleotide	Pyrimidine Metabolism, Cytidine containing	1.3225	0.9999	1.0001	1.0158	1.1027	1.2667	1.3876	1.1029
5-methyltetrahydrofolate (5MeTHF)	Cofactors and Vitami	Folate Metabolism	1.7370	2.1028	2.7831	1.8764	0.5395	0.7349	0.8369	0.8228
5-methylthioadenosine (MTA)	Amino Acid	Polyamine Metabolism	1.0739	1.0085	0.8668	1.1989	0.7642	0.9634	0.8654	0.8422
5-methyluridine (ribothymidine)	Nucleotide	Pyrimidine Metabolism, Uracil containing	2.2493	1.7425	1.3703	1.3505	1.0325	0.8990	0.8738	0.3461
5-oxoproline	Amino Acid	Glutathione Metabolism	0.9287	0.9528	0.8901	1.0659	0.7930	0.9684	0.7205	0.7411
6-oxopiperidine-2-carboxylate	Amino Acid	Lysine Metabolism	1.1937	1.1078	0.8912	1.1616	0.7917	0.7872	0.7263	0.7586
6-phosphogluconate	Carbohydrate	Pentose Phosphate Pathway	0.3685	0.2884	0.2473	0.4232	9.4483	13.0584	2.9119	6.8153
7-dehydrocholesterol	Lipid	Sterol	0.4126	0.4948	0.3413	0.4708	1.7989	1.9061	1.3784	1.5503
7-hydroxycholesterol (alpha or beta)	Lipid	Sterol	0.6760	0.7747	0.8040	0.8214	2.0602	1.9063	0.9576	1.2052
7-methylguanine	Nucleotide	Purine Metabolism, Guanine containing	1.3655	0.9827	1.1615	1.3234	1.0985	1.5323	1.3788	1.2560
9-hydroxystearate	Lipid	Fatty Acid, Monohydroxy	1.8967	1.6119	5.7125	1.0000	1.8671	1.6592	0.9854	0.5552
acetyl-CoA	Lipid	Fatty Acid Metabolism	0.3333	0.3273	0.4982	1.3802	3.6340	1.6588	2.1250	1.7720
acetylcarnitine (C2)	Lipid	Fatty Acid Metabolism (Acyl Carnitine, Short Chain)	1.2279	1.0881	1.0448	1.1303	1.3380	1.4522	0.6866	1.2306
acetylphosphate	Energy	Oxidative Phosphorylation	0.6883	0.1061	0.1061	0.7600	0.1061	9.3269	3.1938	4.3026
aconitate [cis or trans]	Energy	TCA Cycle	0.8665	0.7024	0.5978	0.6275	2.4444	3.6419	2.6107	1.9169
adenine	Nucleotide	Purine Metabolism, Adenine containing	1.2157	1.3667	1.4516	1.8286	0.7510	0.6577	0.5215	1.0213
adenosine	Nucleotide	Purine Metabolism, Adenine containing	0.7335	0.6821	1.4229	1.5227	0.9857	0.8629	0.8802	1.0143
adenosine 3',5'-cyclic monophosphate (cAMP)	Nucleotide	Purine Metabolism, Adenine containing	1.2976	1.2996	1.2573	1.5242	0.4032	0.5722	0.5717	0.4964
ADP	Nucleotide	Purine Metabolism, Adenine containing	1.1348	0.7459	0.4239	0.7217	1.8640	2.6176	1.0918	1.1249
adenosine 5'-diphosphoribose (ADP-ribose)	Cofactors and Vitami	Nicotinate and Nicotinamide Metabolism	0.6385	0.5178	0.6326	0.6254	8.5347	9.2453	1.3488	4.6311
AMP	Nucleotide	Purine Metabolism, Adenine containing	1.2781	0.9992	0.7545	1.1967	0.5503	0.6501	0.6574	0.7406
ATP	Nucleotide	Purine Metabolism, Adenine containing	1.5204	0.8080	0.3648	0.7932	14.1908	17.1349	4.9973	5.1550
adenosine-5'-diphosphoglucose	Carbohydrate	Nucleotide Sugar	0.3064	0.3064	0.3064	0.3064	2.7063	2.2981	1.0000	2.9913
adenylosuccinate	Nucleotide	Purine Metabolism, Adenine containing	2.2653	1.8918	0.9522	2.0585	0.3701	0.2730	0.4306	0.3059

adrenoylcarnitine (C22:4)*	Lipid	Fatty Acid Metabolism (Acyl Carnitine, Polyunsaturated)	0.9507	0.8754	1.1943	0.4119	1.1757	2.0121	0.3821	1.0000
AICA ribonucleotide	Nucleotide	Purine Metabolism, (Hypo)Xanthine/Inosine containing	2.3366	1.0000	4.6103	1.1399	0.3921	0.3921	0.4881	0.4263
alanine	Amino Acid	Alanine and Aspartate Metabolism	0.8469	0.9284	0.8347	1.2169	0.8364	0.8112	0.7690	0.9176
alanylleucine	Peptide	Dipeptide	0.3920	0.3920	0.3920	0.3920	0.8441	0.3920	0.3920	0.3920
allantoin	Nucleotide	Purine Metabolism, (Hypo)Xanthine/Inosine containing	1.0598	1.0410	0.9995	1.0640	1.0005	1.0575	0.8617	0.9885
alpha-hydroxyisocaproate	Amino Acid	Leucine, Isoleucine and Valine Metabolism	1.6681	1.0907	0.9625	1.4409	0.5817	0.5817	0.5817	0.5817
alpha-hydroxyisovalerate	Amino Acid	Leucine, Isoleucine and Valine Metabolism	1.5017	1.2927	1.4923	1.3416	0.3244	0.3134	0.4332	0.2257
alpha-ketoglutaramate*	Amino Acid	Glutamate Metabolism	1.0504	1.0515	1.0000	1.2324	0.1815	0.5137	0.3923	0.3962
alpha-ketoglutarate	Energy	TCA Cycle	2.5719	2.0000	2.5072	1.4196	0.7655	0.8153	0.7799	0.8418
alpha-tocopherol	Cofactors and Vitami	Tocopherol Metabolism	1.0636	0.9177	0.8154	1.1123	1.3997	0.8480	0.7024	0.7787
arabinose	Carbohydrate	Pentose Metabolism	0.5750	0.5564	0.8524	0.5075	2.1703	1.3473	1.5789	2.6238
arabitol/xylitol	Carbohydrate	Pentose Metabolism	1.1013	0.9878	1.1396	0.9174	1.0439	0.7859	0.8348	1.2162
arabonate/xylonate	Carbohydrate	Pentose Metabolism	0.7851	0.6364	0.8180	0.7106	0.9876	1.0124	1.1176	1.6604
arachidate (20:0)	Lipid	Long Chain Saturated Fatty Acid	0.6558	0.4341	1.8704	0.6142	2.1882	3.3638	2.2066	1.4411
arachidonate (20:4n6)	Lipid	Long Chain Polyunsaturated Fatty Acid (n3 and n6)	0.1938	0.1054	0.4534	0.2306	14.5155	17.4606	12.0011	5.9213
arachidonoyl CoA	Lipid	Fatty Acid Metabolism	0.8060	0.7925	0.7925	0.7925	1.9886	2.7919	0.7925	2.2911
arachidonoyl ethanolamide	Lipid	Endocannabinoid	0.3911	0.3911	0.3911	0.3911	1.9163	1.5966	0.3911	1.0000
arachidonoylcarnitine (C20:4)	Lipid	Fatty Acid Metabolism (Acyl Carnitine, Polyunsaturated)	0.7764	0.8752	1.2826	0.4698	1.1248	1.5355	0.4064	1.1789
arachidonoylcholine	Lipid	Fatty Acid Metabolism (Acyl Choline)	0.5923	0.5923	0.5923	0.5923	0.5923	0.5923	0.5923	0.5923
arachidoylcarnitine (C20)*	Lipid	Fatty Acid Metabolism (Acyl Carnitine, Long Chain Saturated)	0.6765	1.1564	1.9595	0.9504	1.1292	0.4624	0.9003	0.8412
arginine	Amino Acid	Urea cycle; Arginine and Proline Metabolism	0.6567	0.6786	0.6920	0.8933	1.4043	1.5794	1.2815	1.5012
argininosuccinate	Amino Acid	Urea cycle; Arginine and Proline Metabolism	0.8159	1.2145	1.5482	1.4691	0.8453	0.6416	1.1966	0.6669
asparagine	Amino Acid	Alanine and Aspartate Metabolism	0.7833	0.6817	0.7193	1.0791	0.9474	0.9144	0.8584	1.1124
aspartate	Amino Acid	Alanine and Aspartate Metabolism	0.6450	0.6928	0.9050	0.8298	1.3448	1.0498	1.2918	1.7491
behenoyl dihydrosphingomyelin (d18:0/22:0)*	Lipid	Dihydrosphingomyelins	0.6995	0.7594	0.2292	1.1976	2.1804	2.2620	0.7778	0.8159
behenoyl sphingomyelin (d18:1/22:0)*	Lipid	Sphingomyelins	0.5328	0.8041	0.6093	0.8303	2.9857	1.5983	0.7435	1.0197
benzoate	Xenobiotics	Benzoate Metabolism	0.9435	1.5843	0.9446	1.6135	2.0455	2.2632	0.7535	1.9370
beta-alanine	Nucleotide	Pyrimidine Metabolism, Uracil containing	2.6293	2.1107	2.1265	2.3184	0.5339	0.3716	0.2874	0.5127
beta-citrylglutamate	Amino Acid	Glutamate Metabolism	1.0794	1.0385	1.1480	1.0743	1.1656	1.2067	1.2230	1.2095
beta-guanidinopropanoate	Xenobiotics	Food Component/Plant	1.5110	1.6605	1.0595	1.6908	0.7576	0.8002	0.6160	1.2286
beta-hydroxyisovalerate	Amino Acid	Leucine, Isoleucine and Valine Metabolism	1.5736	1.1543	1.1756	1.4189	0.5269	0.4135	0.4137	0.3609
beta-hydroxyisovaleroylcarnitine	Amino Acid	Leucine, Isoleucine and Valine Metabolism	0.9386	0.8673	0.9892	1.0108	2.3106	2.8837	2.4630	3.1410
beta-sitosterol	Lipid	Sterol	0.6928	0.6928	0.6928	0.6928	0.6928	0.6928	0.6928	0.6928
betaine	Amino Acid	Glycine, Serine and Threonine Metabolism	1.4220	1.3015	1.4456	1.4426	0.8187	1.1712	0.7642	0.9724
bilirubin	Cofactors and Vitami	Hemoglobin and Porphyrin Metabolism	0.8832	1.5212	0.7917	1.1208	0.5651	0.5651	0.5651	0.5651
biotin	Cofactors and Vitami	Biotin Metabolism	0.6127	0.6127	0.6127	1.5160	0.6127	0.6127	0.6127	0.6127
branched-chain, straight-chain, or cyclopropyl 12	Partially Characterize	Partially Characterized Molecules	0.9067	0.7785	1.8675	1.0774	1.6359	1.5959	2.0492	0.9750
butyrate/isobutyrate (4:0)	Lipid	Short Chain Fatty Acid	0.5948	0.2870	0.6296	0.2870	0.2870	1.4536	1.9934	1.3525
butyrylcarnitine (C4)	Lipid	Fatty Acid Metabolism (also BCAA Metabolism)	1.6449	1.4717	1.6172	1.4454	0.9203	1.2256	0.7021	0.9323
C-glycosyltryptophan	Amino Acid	Tryptophan Metabolism	0.9483	0.8031	1.0703	0.7941	3.5756	3.9538	4.2244	4.2231
cadaverine	Amino Acid	Lysine Metabolism	1.1531	0.9698	0.8762	1.0302	0.8762	0.8762	0.8762	0.8762
campesterol	Lipid	Sterol	0.9739	0.9640	0.7515	0.7515	0.7515	0.9286	0.7515	0.8096
carboxyethyl-GABA	Amino Acid	Glutamate Metabolism	0.2614	0.2562	0.3290	0.2531	2.9746	2.3214	2.6750	2.7066
carnitine	Lipid	Carnitine Metabolism	1.2013	1.1860	1.1199	1.5469	1.9931	1.6641	1.9403	3.1077
carnosine	Amino Acid	Histidine Metabolism	0.5492	0.5280	0.6253	0.6417	0.7857	0.9621	0.8102	0.9383
catechol sulfate	Xenobiotics	Benzoate Metabolism	0.3965	0.3965	0.3965	0.3965	1.0341	1.2833	1.6111	0.9136
ceramide (d16:1/24:1, d18:1/22:1)*	Lipid	Ceramides	0.5708	0.6026	0.5402	1.2260	1.4075	0.1817	0.1817	0.1817
ceramide (d18:1/14:0, d16:1/16:0)*	Lipid	Ceramides	0.8648	0.9057	0.6540	1.1379	1.3302	0.6627	0.4184	0.6191
ceramide (d18:1/17:0, d17:1/18:0)*	Lipid	Ceramides	0.5460	0.7052	0.4146	1.3942	3.5779	1.1482	0.6524	1.0409
ceramide (d18:2/24:1, d18:1/24:2)*	Lipid	Ceramides	0.8745	1.0032	0.9286	1.2146	2.8138	0.9968	0.8580	1.0976
cholesterol	Lipid	Sterol	0.9993	1.1070	0.9897	1.1694	1.4126	1.3194	0.9564	1.2667
choline	Lipid	Phospholipid Metabolism	0.8490	0.7669	0.8533	0.9846	1.6648	1.8298	1.8227	1.6898
phosphocholine	Lipid	Phospholipid Metabolism	0.7380	0.8200	0.6596	1.2796	0.9078	0.8226	0.8037	1.0922
cis-4-decenoylcarnitine (C10:1)	Lipid	Fatty Acid Metabolism (Acyl Carnitine, Monounsaturated)	1.0056	1.0442	1.1227	0.7518	0.7518	2.3450	0.7518	0.7518
citrate	Energy	TCA Cycle	0.9618	0.8021	0.6907	0.8348	1.7209	2.4041	1.9783	1.4436
citrulline	Amino Acid	Urea cycle; Arginine and Proline Metabolism	1.0650	0.9739	0.9104	1.3465	0.8124	0.9041	0.7746	0.8497
CoA-glutathione*	Amino Acid	Glutathione Metabolism	0.7841	0.6736	1.1483	0.9732	1.7526	2.2259	2.6914	2.3773
CoA	Cofactors and Vitami	Pantothenate and CoA Metabolism	0.6567	0.6676	1.0475	1.0380	0.7712	1.0460	1.2582	1.0715
creatine	Amino Acid	Creatine Metabolism	1.1786	1.0060	0.9940	1.2292	1.3760	1.4202	1.1745	1.4125
creatine phosphate	Amino Acid	Creatine Metabolism	1.8274	1.3952	1.2322	1.6225	1.2126	1.3465	0.8264	0.9902
creatinine	Amino Acid	Creatine Metabolism	1.2343	1.0413	0.9553	1.3568	1.4321	1.7747	1.2996	1.4004
cyclic dGSH	Amino Acid	Glutathione Metabolism	0.9551	1.1131	0.8965	1.0248	0.8171	1.0254	0.7533	0.7493
cystathionine	Amino Acid	Methionine, Cysteine, SAM and Taurine Metabolism	4.7811	5.3016	4.5865	4.8581	0.7346	0.7346	0.7346	0.7346
cysteine	Amino Acid	Methionine, Cysteine, SAM and Taurine Metabolism	0.6196	0.6379	0.6997	1.2446	0.6399	0.9330	0.8194	0.9886
cysteine-glutathione disulfide	Amino Acid	Glutathione Metabolism	0.5821	0.5425	0.6288	1.0959	0.5256	1.0930	1.0641	1.5210
cysteinylglycine	Amino Acid	Glutathione Metabolism	0.7559	0.7429	0.6614	0.9619	0.5029	0.5640	0.4425	0.4998
cytidine	Nucleotide	Pyrimidine Metabolism, Cytidine containing	0.5638	0.6470	1.0396	0.9932	1.6596	1.7069	2.0738	1.8775
CDP-choline	Lipid	Phospholipid Metabolism	0.6240	0.5627	0.6694	0.6713	1.2431	1.3886	1.2331	1.1881
CMP	Nucleotide	Pyrimidine Metabolism, Cytidine containing	1.0346	0.9437	0.9305	1.2215	0.5411	0.5772	0.7320	0.6510
cytidine 5'-monophospho-N-acetylneuraminic ac	Carbohydrate	Nucleotide Sugar	1.1766	0.9672	0.9365	1.2483	0.6753	0.7076	0.8659	0.8055
cytidine diphosphate	Nucleotide	Pyrimidine Metabolism, Cytidine containing	1.0954	0.8282	0.4969	0.8720	3.8044	4.9027	2.9103	2.1542
cytidine triphosphate	Nucleotide	Pyrimidine Metabolism, Cytidine containing	0.6370	0.3475	0.3475	0.3475	10.9132	16.1421	3.8368	3.0386
CDP-ethanolamine	Lipid	Phospholipid Metabolism	0.5606	0.4938	0.5884	0.5655	1.6888	1.5303	1.4633	1.9049
cytosine	Nucleotide	Pyrimidine Metabolism, Cytidine containing	0.5300	0.5300	0.5300	0.5300	3.1420	2.9452	3.4078	2.3266
decanoylcarnitine (C10)	Lipid	Fatty Acid Metabolism (Acyl Carnitine, Medium Chain)	0.5507	0.8878	1.4676	0.5469	0.5469	0.5469	0.5469	0.5469
deoxycarnitine	Lipid	Carnitine Metabolism	1.0315	0.8955	1.1557	1.0555	0.9685	0.7768	0.7465	1.2985
diacetylspermidine*	Amino Acid	Polyamine Metabolism	0.6008	0.6008	0.6008	0.6008	0.6008	0.6008	0.6008	0.6008
diadenosine triphosphate	Nucleotide	Purine Metabolism, Adenine containing	0.8503	0.9933	1.1703	1.0136	0.8727	1.1562	1.4187	0.9020
dihomolinoleate (20:2n6)	Lipid	Long Chain Polyunsaturated Fatty Acid (n3 and n6)	0.2666	0.2102	0.9879	0.3887	9.1103	9.2427	6.9411	4.3935
dihomolinolenate (20:3n3 or 3n6)	Lipid	Long Chain Polyunsaturated Fatty Acid (n3 and n6)	0.3865	0.2508	0.8919	0.4758	11.4895	15.1506	12.8175	6.6893
dihomo-linolenoylcarnitine (C20:3n3 or 6)*	Lipid	Fatty Acid Metabolism (Acyl Carnitine, Polyunsaturated)	0.9525	0.9572	1.3091	0.4282	1.6344	1.8286	0.5850	1.0428
dihomo-linoleoylcarnitine (C20:2)*	Lipid	Fatty Acid Metabolism (Acyl Carnitine, Polyunsaturated)	0.9595	0.8587	1.3794	0.4055	1.0405	1.2957	0.3497	0.6021
dihydroorotate	Nucleotide	Pyrimidine Metabolism, Orotate containing	1.8725	1.3712	4.3672	0.3261	0.3261	0.3261	0.3261	0.3261
dihydroxyacetone phosphate (DHAP)	Carbohydrate	Glycolysis, Gluconeogenesis, and Pyruvate Metabolism	0.8986	0.9039	1.3583	1.0961	8.2379	1.1449	0.2131	0.4042
dimethylarginine (ADMA + SDMA)	Amino Acid	Urea cycle; Arginine and Proline Metabolism	0.5920	0.6031	0.7342	0.8839	0.7930	1.1043	1.2573	1.2940
dimethylglycine	Amino Acid	Glycine, Serine and Threonine Metabolism	1.6866	1.3216	1.3819	1.5179	1.0523	1.3192	1.0781	0.8451
dimethylmalonic acid	Lipid	Fatty Acid, Dicarboxylate	0.7559	0.5904	1.0855	0.4950	1.2288	0.4950	0.4950	1.4634
docosadienoate (22:2n6)	Lipid	Long Chain Polyunsaturated Fatty Acid (n3 and n6)	0.6274	0.3598	2.5019	0.4522	3.7740	3.4451	2.4130	1.6469
docosahexaenoate (DHA; 22:6n3)	Lipid	Long Chain Polyunsaturated Fatty Acid (n3 and n6)	0.2961	0.1546	0.9751	0.2336	6.9813	6.9474	5.1776	2.4936
docosahexaenoylcarnitine (C22:6)*	Lipid	Fatty Acid Metabolism (Acyl Carnitine, Polyunsaturated)	1.2599	1.0000	1.9871	0.2331	0.3956	0.7219	0.2331	0.2331
docosahexaenoylcholine	Lipid	Fatty Acid Metabolism (Acyl Choline)	0.2145	0.2145	0.2145	0.2145	1.7747	1.7489	1.1572	1.5833
docosapentaenoate (DPA; 22:5n3)	Lipid	Long Chain Polyunsaturated Fatty Acid (n3 and n6)	0.2135	0.1124	0.6077	0.2107	12.6463	14.3855	9.0659	4.5061
docosapentaenoate (n6 DPA; 22:5n6)	Lipid	Long Chain Polyunsaturated Fatty Acid (n3 and n6)	0.3590	0.2245	1.0157	0.3641	8.4214	13.2613	8.2361	4.6183
docosapentaenoylcarnitine (C22:5n3)*	Lipid	Fatty Acid Metabolism (Acyl Carnitine, Polyunsaturated)	1.0546	0.9454	1.2607	0.2782	1.2602	1.8434	0.2935	0.8658
docosatrienoate (22:3n3)	Lipid	Long Chain Polyunsaturated Fatty Acid (n3 and n6)	0.5593	0.3390	1.3515	0.4781	5.2117	8.7367	4.1625	2.8601
docosatrienoate (22:3n6)*	Lipid	Long Chain Polyunsaturated Fatty Acid (n3 and n6)	0.3854	0.1773	1.0696	0.3405	4.7963	8.7344	5.1643	3.1752
dodecadienoate (12:2)*	Lipid	Fatty Acid, Dicarboxylate	1.7489	0.8203	0.5247	1.0579	2.1819	2.9080	1.9273	2.2020
eicosanoylsphingosine (d20:1)*	Lipid	Sphingosines	0.4993	0.5720	0.2686	0.8401	1.0310	0.9903	0.2686	0.6982
eicosapentaenoate (EPA; 20:5n3)	Lipid	Long Chain Polyunsaturated Fatty Acid (n3 and n6)	0.2666	0.1656	0.6715	0.2838	18.8649	14.4699	10.5944	4.5260
eicosenoate (20:1n9 or 1n11)	Lipid	Long Chain Monounsaturated Fatty Acid	0.5986	0.3661	2.4965	0.5152	5.5006	4.8592	3.4896	2.6904
eicosenoylcarnitine (C20:1)*	Lipid	Fatty Acid Metabolism (Acyl Carnitine, Monounsaturated)	1.2109	1.1283	2.6913	0.8250	0.8717	0.8594	0.5061	0.7031
ergothioneine	Xenobiotics	Food Component/Plant	1.3761	1.1786	1.5141	1.1817	0.9289	0.8228	0.8201	1.6276
erucate (22:1n9)	Lipid	Long Chain Monounsaturated Fatty Acid	0.7317	0.4367	3.3398	0.5728	3.4072	4.3776	2.9251	2.6374
erucoylcarnitine (C22:1)*	Lipid	Fatty Acid Metabolism (Acyl Carnitine, Monounsaturated)	0.6814	0.9116	1.5467	0.7799	1.0727	0.6814	0.6814	0.6814
erythritol	Xenobiotics	Food Component/Plant	1.1607	1.1101	1.2101	1.1528	0.6318	0.6811	0.5804	0.6531
erythronate*	Carbohydrate	Aminosugar Metabolism	1.3824	1.1113	1.1083	1.3262	0.2491	0.2059	0.1913	0.2668
ethyl beta-glucopyranoside	Xenobiotics	Food Component/Plant	0.4717	1.0144	1.0647	7.6914	7.1322	1.6326	1.1049	0.3691
ethylmalonate	Amino Acid	Leucine, Isoleucine and Valine Metabolism	2.2125	1.9231	1.6767	2.3780	0.2458	0.3316	0.2347	0.2908
FAD	Cofactors and Vitami	Riboflavin Metabolism	0.7362	0.8559	0.8449	1.1842	1.2090	1.4744	1.1779	1.1950
FMN	Cofactors and Vitami	Riboflavin Metabolism	0.9171	0.9610	1.0041	1.2419	0.8326	1.0294	1.0474	0.7783
folate	Cofactors and Vitami	Folate Metabolism	0.6519	0.6519	0.6519	0.6519	0.6519	0.6519	0.6519	0.6519
formiminoglutamate	Amino Acid	Histidine Metabolism	0.8895	0.8353	1.0061	0.9033	1.1960	1.9161	1.6135	0.9939
fructose	Carbohydrate	Fructose, Mannose and Galactose Metabolism	0.4105	0.3120	0.1885	0.4138	8.0198	10.7040	1.3944	4.5168

Isobar: hexose diphosphates	Carbohydrate	Glycolysis, Gluconeogenesis, and Pyruvate Metabolism	0.6342	0.5019	0.2829	0.6701	13.3241	5.9693	2.3101	2.1884
fructosyllysine	Amino Acid	Lysine Metabolism	0.4483	1.1740	0.5604	1.0056	0.6568	2.1755	0.9882	0.6832
fumarate	Energy	TCA Cycle	1.1819	0.9933	1.2942	1.2599	1.0831	1.0067	1.2698	0.9359
galactitol (dulcitol)	Carbohydrate	Fructose, Mannose and Galactose Metabolism	0.8821	0.6002	0.7272	0.7560	2.6132	1.8290	1.8946	2.7282
galactonate	Carbohydrate	Fructose, Mannose and Galactose Metabolism	2.8203	2.3886	2.7118	2.1945	0.5980	0.5980	0.5980	0.5980
galactosylglycerol	Lipid	Galactosyl Glycerolipids	1.4011	1.5277	1.7155	1.4181	0.1827	0.1787	0.1413	0.2399
gamma-glutamylcysteine	Peptide	Gamma-glutamyl Amino Acid	0.8436	0.8900	0.9630	1.0871	0.5697	0.6825	0.7729	0.6840
gamma-glutamylglutamate	Peptide	Gamma-glutamyl Amino Acid	1.0018	0.9445	0.9961	1.0230	0.9443	1.5578	1.7140	1.3086
gamma-glutamylglutamine	Peptide	Gamma-glutamyl Amino Acid	0.1918	0.2304	0.2663	0.2690	1.2728	1.3599	1.2022	1.8927
gamma-glutamylhistidine	Peptide	Gamma-glutamyl Amino Acid	0.2562	0.2562	0.4659	0.4366	0.8346	0.2562	0.6124	0.6895
gamma-glutamylisoleucine*	Peptide	Gamma-glutamyl Amino Acid	0.4683	0.4566	0.4338	0.5972	1.4390	1.4429	1.3679	1.6664
gamma-glutamylleucine	Peptide	Gamma-glutamyl Amino Acid	0.2688	0.2735	0.2650	0.3596	1.4014	1.2951	1.1236	1.6848
gamma-glutamylmethionine	Peptide	Gamma-glutamyl Amino Acid	0.3598	0.4406	0.6340	0.5269	1.0090	1.5317	1.2882	1.1630
gamma-glutamylphenylalanine	Peptide	Gamma-glutamyl Amino Acid	0.6575	0.7511	0.7839	0.9921	0.5986	0.6185	0.6031	0.8123
gamma-glutamylthreonine	Peptide	Gamma-glutamyl Amino Acid	0.1463	0.1602	0.1104	0.2706	1.4091	1.9397	1.7073	2.1611
gamma-glutamyltryptophan	Peptide	Gamma-glutamyl Amino Acid	0.7478	0.7478	0.7478	0.7478	0.7478	0.7478	0.7478	0.7478
gamma-glutamyltyrosine	Peptide	Gamma-glutamyl Amino Acid	0.5350	0.6871	0.5356	1.0122	0.5350	0.5350	0.5350	0.5350
gamma-glutamylvaline	Peptide	Gamma-glutamyl Amino Acid	0.2961	0.2812	0.2458	0.4186	1.4683	1.5277	1.4223	1.7532
gluconate	Xenobiotics	Food Component/Plant	1.0002	0.3030	0.1874	0.4578	3.3481	3.5906	0.9998	3.7395
glucosamine-6-phosphate	Carbohydrate	Aminosugar Metabolism	0.0262	0.0262	0.0262	0.0262	2.8402	6.4625	1.0230	2.0632
glucose	Carbohydrate	Glycolysis, Gluconeogenesis, and Pyruvate Metabolism	0.6583	0.5417	0.4553	0.6127	3.0045	110.1271	1.1427	2.0171
glucose 6-phosphate	Carbohydrate	Glycolysis, Gluconeogenesis, and Pyruvate Metabolism	0.3339	0.2191	0.2346	0.3887	9.3269	17.7428	3.3685	5.8033
glucuronate	Carbohydrate	Aminosugar Metabolism	1.1583	0.8732	0.6244	1.2401	1.3809	1.0847	0.8962	1.2153
glutamate	Amino Acid	Glutamate Metabolism	1.0076	0.8545	0.9451	1.0780	1.0375	0.9186	0.9723	1.0921
glutamate, gamma-methyl ester	Amino Acid	Glutamate Metabolism	0.8565	0.8584	1.7128	1.0313	0.4140	0.3963	0.2858	0.4099
glutamine	Amino Acid	Glutamate Metabolism	0.9568	0.9830	0.8932	1.2631	0.6816	0.5972	0.3944	0.8447
glutarate (C5-DC)	Lipid	Fatty Acid, Dicarboxylate	0.9395	0.8809	0.9727	1.0908	0.8879	0.7410	1.5994	0.5142
glutarylcarnitine (C5-DC)	Amino Acid	Lysine Metabolism	0.3885	0.3885	0.3885	0.3885	3.8434	3.1149	4.0244	3.9807
glutathione, oxidized (GSSG)	Amino Acid	Glutathione Metabolism	1.0222	0.8329	0.7994	1.1004	0.9083	1.0272	0.9437	1.1228
glutathione, reduced (GSH)	Amino Acid	Glutathione Metabolism	0.8859	0.8139	0.6897	1.1994	1.2259	1.0532	0.9888	1.1018
glycerate	Carbohydrate	Glycolysis, Gluconeogenesis, and Pyruvate Metabolism	0.5087	0.4316	0.4194	0.6213	3.2300	3.3042	1.1191	2.3021
glycerol	Lipid	Glycerolipid Metabolism	0.9923	1.0297	1.2128	1.2779	0.7255	1.0787	0.9987	0.8685
glycerol 3-phosphate	Lipid	Glycerolipid Metabolism	0.4152	0.4426	0.6621	0.9968	3.1299	3.0961	3.7672	2.8415
glycerophosphoethanolamine	Lipid	Phospholipid Metabolism	0.3305	0.2728	0.2294	0.4359	1.3103	1.0337	0.9669	1.3123
glycerophosphoglycerol	Lipid	Glycerolipid Metabolism	1.0082	0.8906	1.2229	0.8924	0.9918	0.8577	0.9242	1.0762
glycerophosphoinositol*	Lipid	Phospholipid Metabolism	1.2727	1.0654	0.9106	1.1903	1.7937	1.9776	1.8072	2.2629
glycerophosphorylcholine (GPC)	Lipid	Phospholipid Metabolism	0.2419	0.1803	0.1135	0.3865	1.7123	1.5781	1.2008	1.6115
glycerophosphoserine*	Lipid	Phospholipid Metabolism	1.2694	1.1566	1.4837	1.4150	0.5808	0.5542	0.4799	0.6149
glycine	Amino Acid	Glycine, Serine and Threonine Metabolism	1.2503	1.1774	1.2697	1.3649	0.7471	0.6660	0.6636	0.8233
glycochenodeoxycholate	Lipid	Primary Bile Acid Metabolism	0.5766	0.5766	0.5766	0.5766	0.5766	0.5766	0.5766	0.5766
glycodeoxycholate	Lipid	Secondary Bile Acid Metabolism	0.4640	0.4640	0.4640	0.4640	0.4640	0.4640	0.4640	0.4640
glycosyl ceramide (d16:1/24:1, d18:1/22:1)*	Lipid	Hexosylceramides (HCER)	0.6447	0.2886	0.6002	0.9519	2.0908	0.7027	0.5653	1.0513
glycosyl ceramide (d18:1/20:0, d16:1/22:0)*	Lipid	Hexosylceramides (HCER)	0.5373	0.5468	0.4364	0.7698	8.3191	3.1408	2.5211	3.4873
glycosyl ceramide (d18:1/23:1, d17:1/24:1)*	Lipid	Hexosylceramides (HCER)	0.5645	0.8123	0.6709	0.8779	2.7998	1.0549	0.6053	1.0060
glycosyl ceramide (d18:2/24:1, d18:1/24:2)*	Lipid	Hexosylceramides (HCER)	0.8864	1.0030	0.8678	0.9766	3.0797	1.4004	1.0226	1.2796
glycosyl-N-behenoyl-sphingadienine (d18:2/22:0	Lipid	Hexosylceramides (HCER)	0.7305	0.8291	0.6797	0.9795	2.7621	1.2357	0.8226	0.9457
glycosyl-N-palmitoyl-sphingosine (d18:1/16:0)	Lipid	Hexosylceramides (HCER)	0.8843	0.9947	0.7512	1.0053	1.6606	0.8839	0.5134	0.7542
glycosyl-N-stearoyl-sphinganine (d18:0/18:0)*	Lipid	Hexosylceramides (HCER)	0.3664	0.3664	0.3664	0.3664	4.8888	0.9091	0.3664	1.0909
glycosyl-N-stearoyl-sphingosine (d18:1/18:0)	Lipid	Hexosylceramides (HCER)	0.7808	0.9013	0.7465	0.9384	5.5552	2.4946	1.3175	1.8051
glycylisoleucine	Peptide	Dipeptide	0.1525	0.2404	0.1525	0.4099	0.1525	0.1525	0.1525	0.1525
glycylleucine	Peptide	Dipeptide	0.4526	0.4639	0.3318	0.9040	1.0960	0.7532	0.5507	0.6921
glycylvaline	Peptide	Dipeptide	0.5171	0.4794	0.3542	1.0217	0.9783	0.8329	0.5485	0.4821
guaiacol sulfate	Xenobiotics	Benzoate Metabolism	0.8198	0.5534	0.6712	0.8600	0.5534	1.2231	1.2959	1.1191
guanine	Nucleotide	Purine Metabolism, Guanine containing	0.3957	0.4405	1.4189	1.1779	1.0826	0.9193	1.0651	1.0212
guanosine	Nucleotide	Purine Metabolism, Guanine containing	0.7297	1.0627	1.5605	1.7194	2.2443	2.5735	3.2936	2.6432
guanosine 5'- diphosphate (GDP)	Nucleotide	Purine Metabolism, Guanine containing	1.4664	0.6761	0.5343	0.2839	8.2668	9.2780	5.2134	4.3454
5'- GMP	Nucleotide	Purine Metabolism, Guanine containing	1.3593	1.1108	0.9948	1.4766	0.7787	0.8221	1.0052	1.0111
guanosine 5'-diphospho-fucose	Carbohydrate	Nucleotide Sugar	0.9954	0.9049	0.9496	0.9884	1.5785	1.6221	1.3056	1.2133
gulonate*	Cofactors and Vitami	Ascorbate and Aldarate Metabolism	1.8720	1.5997	1.0978	1.6116	0.1027	0.0907	0.0987	0.1165
heneicosapentaenoate (21:5n3)	Lipid	Long Chain Polyunsaturated Fatty Acid (n3 and n6)	0.0795	0.0795	0.0795	0.0795	2.7006	2.1623	1.4091	0.7125
heptadecasphingosine (d17:1)	Lipid	Sphingosines	0.3548	0.3796	0.1382	1.0881	1.3744	1.1952	0.6730	1.4732
heptanoate (7:0)	Lipid	Medium Chain Fatty Acid	0.9989	0.9218	1.1181	1.1879	1.9183	2.4854	1.7296	1.4880
hexadecadienoate (16:2n6)	Lipid	Long Chain Polyunsaturated Fatty Acid (n3 and n6)	0.6345	0.4051	1.8598	0.4582	6.0685	6.2973	6.7206	2.9327
hexadecasphingosine (d16:1)*	Lipid	Sphingosines	0.2743	0.3262	0.1801	0.7290	1.6590	0.7825	0.4049	1.0375
hexanoylcarnitine (C6)	Lipid	Fatty Acid Metabolism (Acyl Carnitine, Medium Chain)	1.0420	1.0080	1.5536	0.9776	1.4993	1.7916	1.3521	0.9920
hippurate	Xenobiotics	Benzoate Metabolism	0.4971	0.5015	0.4443	0.6527	1.9604	2.6246	1.9222	1.7097
histidine	Amino Acid	Histidine Metabolism	0.6148	0.6666	0.7671	1.0076	1.0518	0.9810	0.9924	1.0762
histidine methyl ester	Amino Acid	Histidine Metabolism	0.8837	0.7884	0.9623	1.1017	0.9118	0.9046	0.8274	0.7639
homocysteine	Amino Acid	Methionine, Cysteine, SAM and Taurine Metabolism	1.1385	0.8885	1.3781	1.1813	0.4785	1.1547	0.8248	0.4785
homostachydrine*	Xenobiotics	Food Component/Plant	1.7116	1.5367	2.0415	1.7858	0.7152	0.8508	0.8094	0.8957
hydroxy-N6,N6,N6-trimethyllysine*	Amino Acid	Lysine Metabolism	0.6512	0.6677	0.8180	0.9651	0.7814	0.6417	0.5816	0.8655
hydroxyasparagine	Amino Acid	Alanine and Aspartate Metabolism	1.3742	1.3653	0.8316	1.8682	0.5260	0.5260	0.5260	0.5260
hypotaurine	Amino Acid	Methionine, Cysteine, SAM and Taurine Metabolism	1.4114	1.2288	1.2765	1.4184	1.4416	0.9520	0.8526	1.7202
hypoxanthine	Nucleotide	Purine Metabolism, (Hypo)Xanthine/Inosine containing	0.7449	1.0058	1.6252	1.4126	0.9598	0.9942	1.2999	1.3681
imidazole lactate	Amino Acid	Histidine Metabolism	4.6856	3.7750	4.5187	4.3610	0.3004	0.2155	0.2520	0.3020
imidazole propionate	Amino Acid	Histidine Metabolism	4.1145	3.4424	3.6169	3.9631	0.1837	0.1784	0.1486	0.1881
indoleacetate	Amino Acid	Tryptophan Metabolism	0.5611	0.5611	0.5611	0.5611	0.5611	1.5740	1.2465	0.9348
indolelactate	Amino Acid	Tryptophan Metabolism	1.0750	0.9250	0.6046	1.1676	0.3710	0.6628	0.8844	0.3671
inosine	Nucleotide	Purine Metabolism, (Hypo)Xanthine/Inosine containing	0.6505	1.0084	1.3687	1.4384	1.8231	1.4567	1.6522	2.0676
inosine 5'-monophosphate (IMP)	Nucleotide	Purine Metabolism, (Hypo)Xanthine/Inosine containing	0.4832	0.9559	3.2081	1.7354	2.6011	3.1578	3.8451	2.8593
inositol 1-phosphate (I1P)	Lipid	Inositol Metabolism	0.6519	0.6602	0.4343	0.9546	4.1621	6.0902	3.9243	3.6577
isobutyrylcarnitine (C4)	Amino Acid	Leucine, Isoleucine and Valine Metabolism	0.9916	0.9294	1.6249	1.0209	2.3110	2.3167	2.2386	2.2375
isocitrate	Energy	TCA Cycle	0.8072	0.5032	0.3312	0.3312	5.6547	10.1969	7.0692	3.5363
isoleucine	Amino Acid	Leucine, Isoleucine and Valine Metabolism	0.8989	0.8610	0.8148	1.1569	1.2006	1.2396	1.0577	1.2073
isoleucylglycine	Peptide	Dipeptide	0.2448	0.2018	0.1356	0.5157	1.1678	0.7632	0.5266	0.8182
isovalerylcarnitine (C5)	Amino Acid	Leucine, Isoleucine and Valine Metabolism	0.9327	0.7966	1.7591	1.0673	2.2909	1.6196	1.4728	1.7180
isovalerylglycine	Amino Acid	Leucine, Isoleucine and Valine Metabolism	1.3292	0.9982	1.2599	0.8451	0.4205	2.4264	2.0212	0.4205
kynurenate	Amino Acid	Tryptophan Metabolism	0.3630	0.3630	0.3630	0.3630	1.6637	2.3969	1.7407	1.2846
kynurenine	Amino Acid	Tryptophan Metabolism	0.7925	0.8139	0.8081	1.2001	0.7442	0.8329	0.6120	0.7336
lactate	Carbohydrate	Glycolysis, Gluconeogenesis, and Pyruvate Metabolism	1.0106	0.9115	0.8169	1.1662	1.1493	1.2034	0.8126	0.9894
lactose	Carbohydrate	Disaccharides and Oligosaccharides	0.3380	0.2938	0.5845	0.5740	4.9498	6.1893	4.3076	2.3385
lactosyl-N-behenoyl-sphingosine (d18:1/22:0)*	Lipid	Lactosylceramides (LCER)	0.6078	0.6853	0.6226	0.7798	4.0951	3.0090	1.5503	1.8697
lactosyl-N-nervonoyl-sphingosine (d18:1/24:1)*	Lipid	Lactosylceramides (LCER)	0.5816	0.6931	0.5911	0.7467	3.2487	2.2853	1.4679	1.6986
lactosyl-N-palmitoyl-sphingosine (d18:1/16:0)	Lipid	Lactosylceramides (LCER)	0.5905	0.7169	0.4670	0.8347	1.5719	1.4057	0.9514	1.1131
lactosyl-N-stearoyl-sphingosine (d18:1/18:0)*	Lipid	Lactosylceramides (LCER)	0.5272	0.6173	0.3859	0.8120	3.3228	3.1476	1.9353	2.1143
laurylcarnitine (C12)	Lipid	Fatty Acid Metabolism (Acyl Carnitine, Medium Chain)	1.3407	1.7458	2.5258	0.9266	0.5247	0.7282	0.8173	0.5788
leucine	Amino Acid	Leucine, Isoleucine and Valine Metabolism	0.8297	0.7940	0.7985	1.0801	1.1687	1.1581	1.0089	1.1368
leucylglutamine*	Peptide	Dipeptide	0.2310	0.2223	0.4092	0.3291	1.0497	0.5824	0.4454	0.4880
leucylglycine	Peptide	Dipeptide	0.3651	0.2540	0.2582	0.4387	1.5804	0.9376	0.4012	0.5917
lignoceroyl sphingomyelin (d18:1/24:0)	Lipid	Sphingomyelins	0.7375	0.8191	0.7887	0.9099	3.9583	1.9356	0.9794	1.3087
linoleate (18:2n6)	Lipid	Long Chain Polyunsaturated Fatty Acid (n3 and n6)	0.4182	0.2502	1.1311	0.4220	6.4565	7.8351	6.3597	3.2984
linolenate (18:3n3 or 3n6)	Lipid	Long Chain Polyunsaturated Fatty Acid (n3 and n6)	0.6358	0.3815	1.8608	0.6322	3.4816	2.8448	2.0812	1.2403
linolenoylcarnitine (C18:3)*	Lipid	Fatty Acid Metabolism (Acyl Carnitine, Polyunsaturated)	1.2275	1.0991	1.4568	0.6042	0.4070	0.9150	0.3749	0.3749
linoleoyl ethanolamide	Lipid	Endocannabinoid	0.6211	0.2815	1.9633	0.3263	5.6847	4.1945	3.7468	1.9891
linoleoylcarnitine (C18:2)*	Lipid	Fatty Acid Metabolism (Acyl Carnitine, Polyunsaturated)	0.8930	0.9060	1.1368	0.4968	1.3838	2.0004	0.5963	1.3607
linoleoylcholine*	Lipid	Fatty Acid Metabolism (Acyl Choline)	0.4257	0.4257	0.4257	0.4257	0.9911	1.2034	1.5454	0.7623
lysine	Amino Acid	Lysine Metabolism	0.7621	0.8124	0.8326	1.0397	1.5383	1.7422	1.4307	1.6055
malate	Energy	TCA Cycle	1.0522	1.1531	0.9147	1.1838	1.2507	1.2068	1.3718	1.0069
maleate	Lipid	Fatty Acid, Dicarboxylate	1.2737	0.8599	1.0984	1.2794	0.9194	0.8121	1.0061	1.0432
malonylcarnitine	Lipid	Fatty Acid Synthesis	0.7781	0.9523	0.8264	0.7402	4.9929	5.2804	5.5191	5.1468
mannitol/sorbitol	Carbohydrate	Fructose, Mannose and Galactose Metabolism	1.0764	1.0496	0.6859	0.9798	0.5368	0.7787	0.3977	0.4501
mannonate*	Xenobiotics	Food Component/Plant	1.0777	1.0523	0.9477	1.1254	0.6003	0.7293	0.7087	1.0696
mannose	Carbohydrate	Fructose, Mannose and Galactose Metabolism	0.1044	0.0704	0.0666	0.0666	0.0666	2.7008	0.0666	0.0666

margarate (17:0)	Lipid	Long Chain Saturated Fatty Acid	0.6280	0.4084	1.8029	0.5917	6.3680	6.7985	4.5729	3.3118
margaroylcarnitine (C17)*	Lipid	Fatty Acid Metabolism (Acyl Carnitine, Long Chain Saturated)	0.9048	0.8671	1.6134	0.6243	1.0819	1.1565	0.6002	0.9181
mead acid (20:3n9)	Lipid	Long Chain Polyunsaturated Fatty Acid (n3 and n6)	0.2514	0.1791	0.8928	0.3317	9.6137	11.7398	7.4596	4.4479
methionine	Amino Acid	Methionine, Cysteine, SAM and Taurine Metabolism	0.7735	0.7835	0.7799	1.0757	1.0869	1.1374	0.9716	1.1130
methionine sulfone	Amino Acid	Methionine, Cysteine, SAM and Taurine Metabolism	0.9145	0.9261	0.8430	1.4490	0.4451	0.5012	0.2638	0.4866
methionine sulfoxide	Amino Acid	Methionine, Cysteine, SAM and Taurine Metabolism	0.6793	0.5521	0.5115	0.8967	1.0796	1.0917	0.9077	1.4329
methyl glucopyranoside (alpha + beta)	Xenobiotics	Food Component/Plant	0.2635	0.2379	0.1755	0.3554	1.9308	1.5629	1.2485	1.8236
methylmalonate (MMA)	Lipid	Fatty Acid Metabolism (also BCAA Metabolism)	0.7380	0.6812	0.2604	0.7930	1.8408	1.0762	1.2313	1.2831
methylphosphate	Nucleotide	Purine and Pyrimidine Metabolism	0.4231	0.3887	0.9661	0.7580	1.0497	0.9875	0.6760	1.0816
methylsuccinate	Amino Acid	Leucine, Isoleucine and Valine Metabolism	1.5892	1.3572	1.6132	1.1782	0.9568	1.4538	0.8039	1.1875
myo-inositol	Lipid	Inositol Metabolism	1.1564	1.1591	1.6276	1.1857	1.4012	1.4999	1.2332	1.4383
myristate (14:0)	Lipid	Long Chain Saturated Fatty Acid	0.9309	0.6650	2.6525	0.8272	2.6305	2.9053	2.0490	1.1591
myristoleate (14:1n5)	Lipid	Long Chain Monounsaturated Fatty Acid	0.9654	0.7719	2.3686	0.8425	1.4220	1.5311	1.7838	0.8800
myristoleoylcarnitine (C14:1)*	Lipid	Fatty Acid Metabolism (Acyl Carnitine, Monounsaturated)	0.7913	1.0464	1.6309	0.5084	0.9566	1.2123	0.5083	1.0434
myristoyl dihydrosphingomyelin (d18:0/14:0)*	Lipid	Dihydrosphingomyelins	0.6929	0.8894	0.6229	1.0883	1.8681	1.3818	0.7759	0.8822
myristoylcarnitine (C14)	Lipid	Fatty Acid Metabolism (Acyl Carnitine, Long Chain Saturated)	1.9384	2.1734	2.7209	1.2516	0.5117	0.6120	0.3856	0.5801
N('1)-acetylspermidine	Amino Acid	Polyamine Metabolism	0.2864	0.2263	0.3001	0.3136	0.0372	0.0446	0.0217	0.0326
N(1)-acetylspermine	Amino Acid	Polyamine Metabolism	0.3810	0.2640	0.3768	0.2720	0.2640	0.2640	0.2640	0.2640
N,N,N-trimethyl-5-aminovalerate	Amino Acid	Lysine Metabolism	1.1062	1.0546	1.3825	1.0227	1.0746	1.0793	0.9658	1.4028
N,N,N-trimethyl-alanylproline betaine (TMAP)	Amino Acid	Urea cycle; Arginine and Proline Metabolism	0.4504	0.4564	0.7052	0.4605	2.5560	2.4628	2.4291	2.9161
N-acetyl-1-methylhistidine*	Amino Acid	Histidine Metabolism	0.3804	0.4103	0.8404	0.3804	0.3804	0.3804	0.3804	0.3804
N-acetyl-3-methylhistidine*	Amino Acid	Histidine Metabolism	0.3770	0.4762	0.6590	0.5691	4.6908	4.2239	3.7842	4.9038
N-acetyl-aspartyl-glutamate (NAAG)	Amino Acid	Glutamate Metabolism	1.2222	1.3422	1.5139	1.1961	0.9093	1.1540	1.1033	0.9039
N-acetyl-cadaverine	Amino Acid	Lysine Metabolism	2.2973	2.3110	2.6346	3.2442	0.1680	1.6421	0.2960	0.3890
N-acetyl-glucosamine 1-phosphate	Carbohydrate	Aminosugar Metabolism	2.6411	2.3529	1.4775	2.0615	0.8720	1.0181	1.1365	0.9963
N-acetyl-isoputreanine	Amino Acid	Polyamine Metabolism	0.4137	0.4151	0.3676	0.4985	1.6150	1.7368	1.6517	1.9974
N-acetylalanine	Amino Acid	Alanine and Aspartate Metabolism	1.2760	1.1003	1.1280	1.3731	0.3990	0.3937	0.3804	0.5326
N-acetylarginine	Amino Acid	Urea cycle; Arginine and Proline Metabolism	0.7124	0.7671	0.7782	0.9415	1.0075	0.9925	1.0270	1.1654
N-acetylasparagine	Amino Acid	Alanine and Aspartate Metabolism	1.2967	1.0680	0.7658	1.4161	0.9455	0.8639	0.9902	0.9647
N-acetylaspartate (NAA)	Amino Acid	Alanine and Aspartate Metabolism	1.2542	1.1636	1.1307	1.4168	0.7807	0.7019	0.7025	0.9111
N-acetylcysteine	Amino Acid	Methionine, Cysteine, SAM and Taurine Metabolism	0.9939	0.9189	0.9010	1.6019	1.7554	1.5391	2.7137	1.4476
N-acetylglucosamine 6-phosphate	Carbohydrate	Aminosugar Metabolism	0.2012	0.1945	0.2325	0.4612	2.0026	2.0951	1.9665	2.0380
N-acetylglucosamine/N-acetylgalactosamine	Carbohydrate	Aminosugar Metabolism	0.6032	0.4566	0.6007	1.2307	3.5131	3.9708	3.8736	3.8481
N-acetylglucosaminylasparagine	Carbohydrate	Aminosugar Metabolism	0.9181	0.8648	0.9259	1.0403	0.7523	0.8170	0.7941	0.9615
N-acetylglutamate	Amino Acid	Glutamate Metabolism	1.9358	1.8987	1.8269	2.1672	0.4705	0.4574	0.5118	0.4138
N-acetylglutamine	Amino Acid	Glutamate Metabolism	1.3706	1.1717	0.9880	1.5171	0.4756	0.4347	0.3919	0.5020
N-acetylhistidine	Amino Acid	Histidine Metabolism	0.4062	0.4594	0.5897	0.6260	4.5183	3.9126	3.7958	4.7014
N-acetylisoleucine	Amino Acid	Leucine, Isoleucine and Valine Metabolism	1.0816	0.6632	0.3784	1.1493	0.3784	0.3784	0.3784	0.3784
N-acetylleucine	Amino Acid	Leucine, Isoleucine and Valine Metabolism	0.7539	0.7030	0.6568	1.1336	0.3300	0.3300	0.3300	0.5822
N-acetylmethionine	Amino Acid	Methionine, Cysteine, SAM and Taurine Metabolism	0.6058	0.5557	0.5144	0.8513	0.8315	1.0962	1.1701	1.2518
N-acetylmethionine sulfoxide	Amino Acid	Methionine, Cysteine, SAM and Taurine Metabolism	0.3243	0.2101	0.1413	0.3948	1.0286	1.4805	1.3785	1.8399
N-acetylneuraminate	Carbohydrate	Aminosugar Metabolism	4.7371	4.7037	4.9231	4.4231	0.5168	0.4127	0.3760	0.4810
N-acetylphenylalanine	Amino Acid	Phenylalanine Metabolism	0.8158	0.7958	0.8085	1.0854	0.3688	0.3624	0.4641	0.5487
N-acetylputrescine	Amino Acid	Polyamine Metabolism	1.5788	1.7244	1.4919	2.7270	0.8077	1.2096	0.1439	0.1950
N-acetylserine	Amino Acid	Glycine, Serine and Threonine Metabolism	2.5600	2.1195	1.6572	2.3747	0.4799	0.5060	0.5041	0.5625
N-acetyltaurine	Amino Acid	Methionine, Cysteine, SAM and Taurine Metabolism	2.4349	1.9254	1.8943	2.2888	0.7482	0.7503	0.6099	0.7886
N-acetylthreonine	Amino Acid	Glycine, Serine and Threonine Metabolism	1.2770	1.1756	1.3288	1.3310	0.4479	0.3365	0.4049	0.6823
N-acetyltryptophan	Amino Acid	Tryptophan Metabolism	0.6238	0.6238	0.6238	0.6238	0.6238	0.6238	0.6238	0.6238
N-acetyltyrosine	Amino Acid	Tyrosine Metabolism	0.5434	0.5408	0.6561	0.7288	1.0482	0.8577	0.8750	0.9990
N-acetylvaline	Amino Acid	Leucine, Isoleucine and Valine Metabolism	0.9737	0.8967	0.9785	1.0424	0.5146	0.5393	0.6452	0.6474
N-arachidonoyltaurine	Lipid	Endocannabinoid	0.3134	0.1553	0.7919	0.2556	3.3560	3.1543	1.5655	0.9647
N-delta-acetylornithine	Amino Acid	Urea cycle; Arginine and Proline Metabolism	0.8120	0.9102	0.9383	1.2770	0.5100	0.5337	0.4831	0.6054
N-formylmethionine	Amino Acid	Methionine, Cysteine, SAM and Taurine Metabolism	0.8720	0.8748	0.9233	1.1316	1.0103	0.8151	0.9702	1.1313
N-glycolylneuraminate	Carbohydrate	Aminosugar Metabolism	1.5617	1.3841	1.6824	1.6422	0.1515	0.1067	0.1067	0.1690
N-linoleoyltaurine*	Lipid	Endocannabinoid	0.2220	0.1033	0.8109	0.2177	3.8670	3.6959	2.0793	1.1606
N-methylproline	Amino Acid	Urea cycle; Arginine and Proline Metabolism	1.2635	0.9835	1.4601	1.3965	1.0165	1.0410	0.5737	1.0903
N-monomethylarginine	Amino Acid	Urea cycle; Arginine and Proline Metabolism	0.3633	0.7914	0.5726	0.9829	1.6663	2.1914	2.1513	2.3760
N-myristoyltaurine*	Lipid	Endocannabinoid	0.2728	0.2728	0.2728	0.2728	1.0185	0.9715	0.5956	0.5945
N-oleoylserine	Lipid	Endocannabinoid	0.5346	0.3634	2.1974	0.4993	1.3569	0.3522	0.6076	0.3522
N-oleoyltaurine	Lipid	Endocannabinoid	0.2020	0.1296	0.5471	0.2452	4.1754	4.5605	2.6093	1.6750
N-palmitoyl-sphingadienine (d18:2/16:0)*	Lipid	Ceramides	0.7036	0.7492	0.5199	1.0160	1.3590	0.7254	0.4948	0.6130
N-palmitoyl-sphinganine (d18:0/16:0)	Lipid	Dihydroceramides	0.5815	0.7141	0.5456	2.2220	1.6884	0.5379	0.1616	0.5298
N-palmitoyl-sphingosine (d18:1/16:0)	Lipid	Ceramides	0.7035	0.7732	0.4807	1.4249	1.9169	0.9047	0.5408	0.7366
N-palmitoylglycine	Lipid	Fatty Acid Metabolism (Acyl Glycine)	1.6994	0.8597	5.6772	1.1403	0.2839	0.2839	0.2839	0.8146
N-palmitoyltaurine	Lipid	Endocannabinoid	0.2261	0.1591	0.6533	0.2786	4.1978	4.6729	2.5028	1.5745
N-stearoyl-sphinganine (d18:0/18:0)*	Lipid	Dihydroceramides	0.1983	0.1983	0.1983	1.2919	1.5138	0.1983	0.1983	0.1983
N-stearoyl-sphingosine (d18:1/18:0)*	Lipid	Ceramides	0.5088	0.6080	0.4434	1.0375	3.1634	1.4393	0.9787	1.4018
N-stearoyltaurine	Lipid	Endocannabinoid	0.3055	0.1929	0.8354	0.3290	4.2433	5.3517	3.0801	2.0894
N1,N12-diacetylspermine	Amino Acid	Polyamine Metabolism	0.3761	0.3761	0.3761	0.3761	0.3761	0.3761	0.3761	0.3761
1-methyladenosine	Nucleotide	Purine Metabolism, Adenine containing	0.3491	0.3386	0.4407	0.4743	1.6306	1.7381	1.5584	1.6744
N1-methylinosine	Nucleotide	Purine Metabolism, (Hypo)Xanthine/Inosine containing	0.3115	0.1184	0.4688	0.3887	4.1439	4.1882	4.6323	4.3911
N2,N2-dimethylguanosine	Nucleotide	Purine Metabolism, Guanine containing	1.0975	0.7369	0.7055	0.8189	1.5409	1.6762	1.7231	1.5388
N2-acetyllysine	Amino Acid	Lysine Metabolism	0.1764	0.1861	0.1764	0.2680	1.5525	1.0000	1.0613	1.7423
N2-methylguanosine	Nucleotide	Purine Metabolism, Guanine containing	0.9214	0.6216	0.5952	0.7426	1.6515	1.8014	1.5107	1.9885
N6,N6,N6-trimethyllysine	Amino Acid	Lysine Metabolism	0.6594	0.6719	0.7677	0.8871	0.6846	0.7538	0.6823	0.7467
N6,N6-dimethyllysine	Amino Acid	Lysine Metabolism	0.9026	0.9530	1.0470	1.1733	0.7368	0.7368	1.6581	0.7368
N6-acetyllysine	Amino Acid	Lysine Metabolism	1.0006	1.1232	1.1342	1.4655	0.9994	1.0883	1.2334	1.1311
N6-carbamoylthreonyladenosine	Nucleotide	Purine Metabolism, Adenine containing	0.7296	0.7768	0.8113	0.8678	1.2738	1.1707	0.9928	1.4656
N6-carboxymethyllysine	Carbohydrate	Advanced Glycation End-product	0.8112	0.7906	0.8237	0.8651	0.9964	0.9048	0.3047	1.0436
N6-methyladenosine	Nucleotide	Purine Metabolism, Adenine containing	0.9005	1.0508	1.1107	1.9090	1.7799	0.5476	2.4208	1.6471
N6-methyllysine	Amino Acid	Lysine Metabolism	0.6933	0.7365	0.7688	1.0748	1.1175	1.4228	1.4796	1.3269
N6-succinyladenosine	Nucleotide	Purine Metabolism, Adenine containing	0.7264	0.9418	0.8074	1.1970	0.8266	0.6029	0.9151	1.0555
nicotinamide	Cofactors and Vitami	Nicotinate and Nicotinamide Metabolism	1.0333	0.9296	0.6244	1.2994	1.9631	2.0308	1.8645	1.9046
NAD+	Cofactors and Vitami	Nicotinate and Nicotinamide Metabolism	1.0033	0.9865	1.3472	1.1934	0.9378	0.9967	0.8367	1.0138
nicotinamide adenine dinucleotide phosphate re	Cofactors and Vitami	Nicotinate and Nicotinamide Metabolism	0.1654	0.7095	0.6740	0.1654	1.7364	3.0974	3.0885	1.2905
NADH	Cofactors and Vitami	Nicotinate and Nicotinamide Metabolism	1.1329	1.3362	2.0339	1.3322	0.9369	0.4931	0.6580	0.3520
nicotinamide ribonucleotide (NMN)	Cofactors and Vitami	Nicotinate and Nicotinamide Metabolism	1.0147	0.7986	1.8459	1.4796	1.6910	1.7336	2.8217	2.0142
nicotinamide riboside	Cofactors and Vitami	Nicotinate and Nicotinamide Metabolism	0.5885	0.4671	0.6741	0.5642	1.2127	1.4174	1.1859	1.5041
nisinate (24:6n3)	Lipid	Long Chain Polyunsaturated Fatty Acid (n3 and n6)	0.6139	0.3099	1.4378	0.3839	1.9957	3.1610	1.6886	1.1253
nonadecanoate (19:0)	Lipid	Long Chain Saturated Fatty Acid	0.5628	0.3906	1.7376	0.5774	5.4473	5.2032	3.2933	2.3416
O-methyltyrosine	Amino Acid	Tyrosine Metabolism	1.4057	1.2611	1.4555	1.3240	1.0298	1.2461	1.1082	0.8680
O-sulfo-L-tyrosine	Xenobiotics	Chemical	0.8935	0.9385	0.9595	1.2948	0.6371	0.7688	0.6455	0.5921
o-Tyrosine	Amino Acid	Tyrosine Metabolism	0.5107	0.3411	0.5671	0.3411	0.3411	1.3450	0.3411	0.7341
octanoylcarnitine (C8)	Lipid	Fatty Acid Metabolism (Acyl Carnitine, Medium Chain)	0.9761	1.0999	1.4636	0.8240	2.1709	1.9273	1.0239	0.8727
oleate/vaccenate (18:1)	Lipid	Long Chain Monounsaturated Fatty Acid	0.5327	0.3525	1.4287	0.6660	4.9291	6.1135	4.8917	3.1259
oleoyl CoA	Lipid	Fatty Acid Metabolism	1.4408	0.9707	0.5957	1.1734	2.5942	2.4911	1.3338	1.3232
oleoyl ethanolamide	Lipid	Endocannabinoid	0.3915	0.4648	0.4896	0.5062	2.4056	1.4245	1.1949	1.5444
oleoyl-arachidonoyl-glycerol (18:1/20:4) [2]*	Lipid	Diacylglycerol	0.5240	0.5240	0.5240	0.5240	8.2470	1.1252	0.5240	0.5240
oleoylcarnitine (C18:1)	Lipid	Fatty Acid Metabolism (Acyl Carnitine, Monounsaturated)	1.2338	1.0469	1.7231	0.6391	0.9531	1.1702	0.3258	0.7355
oleoylcholine	Lipid	Fatty Acid Metabolism (Acyl Choline)	0.1660	0.1671	0.5701	0.1401	1.0339	1.1271	1.7582	0.7379
ophthalmate	Amino Acid	Glutathione Metabolism	0.1096	0.0849	0.0920	0.2306	1.3106	1.2462	0.9870	1.5533
ornithine	Amino Acid	Urea cycle; Arginine and Proline Metabolism	0.4082	0.5691	0.5786	0.8490	0.9395	1.0326	0.9599	1.2801
orotate	Nucleotide	Pyrimidine Metabolism, Orotate containing	2.6011	4.0990	16.3266	8.9852	0.2686	0.2542	0.2047	0.2917
orotidine	Nucleotide	Pyrimidine Metabolism, Orotate containing	20.3813	17.5450	19.5806	16.3674	0.7042	0.7578	0.7848	0.8538
oxaloacetate	Energy	TCA Cycle	1.6792	0.8500	0.9260	1.8417	0.9404	1.0609	1.3126	1.1566
p-cresol sulfate	Xenobiotics	Benzoate Metabolism	0.5400	0.4746	0.5327	0.6733	1.9649	2.1488	2.3226	1.6687
palmitate (16:0)	Lipid	Long Chain Saturated Fatty Acid	0.8608	0.6677	1.5247	0.9643	3.0156	4.1102	3.0072	2.1586
palmitoleate (16:1n7)	Lipid	Long Chain Monounsaturated Fatty Acid	0.7028	0.4627	2.4374	0.7459	4.3830	3.9723	3.2602	1.7311
palmitoleoyl ethanolamide*	Lipid	Endocannabinoid	0.5331	0.8425	1.0772	1.1262	1.6431	0.5331	0.7956	2.1614
palmitoleoylcarnitine (C16:1)*	Lipid	Fatty Acid Metabolism (Acyl Carnitine, Monounsaturated)	1.5200	1.6906	2.1294	0.8985	1.0007	0.9993	0.3240	0.7682
palmitoloelycholine	Lipid	Fatty Acid Metabolism (Acyl Choline)	0.1859	0.1859	0.6908	0.1859	0.9633	0.9058	1.4980	0.6787
palmitoyl dihydrosphingomyelin (d18:0/16:0)*	Lipid	Dihydrosphingomyelins	0.5848	0.7337	0.5053	1.1020	2.6030	1.6004	0.8970	1.2121
palmitoyl ethanolamide	Lipid	Endocannabinoid	0.7405	0.8076	0.7725	0.8946	2.0061	1.2723	0.8453	1.3228

palmitoyl sphingomyelin (d18:1/16:0)	Lipid	Sphingomyelins	0.7864	0.8008	0.6644	0.9397	2.5321	1.8589	1.3936	1.4321
palmitoyl-oleoyl-glycerol (16:0/18:1) [2]*	Lipid	Diacylglycerol	1.6924	2.1840	2.5504	2.2459	0.4453	0.4453	0.4453	0.4453
palmitoylcarnitine (C16)	Lipid	Fatty Acid Metabolism (Acyl Carnitine, Long Chain Saturated)	1.4041	1.4030	2.2909	1.0515	0.7604	0.7447	0.3496	0.5948
palmitoylcholine	Lipid	Fatty Acid Metabolism (Acyl Choline)	0.2182	0.1988	0.6352	0.3212	0.8681	1.0169	1.7528	0.4799
pantetheine	Cofactors and Vitami	Pantothenate and CoA Metabolism	0.5172	0.5223	0.5428	0.8814	2.0849	2.0882	1.6272	2.3063
pantoate	Cofactors and Vitami	Pantothenate and CoA Metabolism	1.1477	0.8666	1.1168	1.0322	1.6670	1.9342	1.5709	1.0861
pantothenate (Vitamin B5)	Cofactors and Vitami	Pantothenate and CoA Metabolism	0.9483	0.8787	0.7688	1.0883	1.1222	1.3755	1.2299	1.3545
penicillin G	Xenobiotics	Drug - Antibiotic	0.6619	0.6206	0.7150	0.6817	2.2728	3.2214	2.2462	1.4369
pentadecanoate (15:0)	Lipid	Long Chain Saturated Fatty Acid	0.8878	0.5445	2.0464	0.7031	4.0143	5.0573	3.5442	2.1635
pentadecanoylcarnitine (C15)*	Lipid	Fatty Acid Metabolism (Acyl Carnitine, Long Chain Saturated)	0.9885	1.1030	1.7802	0.8715	1.1575	0.8006	0.3856	0.9212
phenethylamine	Amino Acid	Phenylalanine Metabolism	0.4305	0.4672	0.5690	0.7396	3.2015	2.5264	2.1374	4.2352
phenol red	Xenobiotics	Chemical	0.7099	0.6805	0.6866	0.8379	2.0362	2.6480	1.7866	1.3788
phenol sulfate	Amino Acid	Tyrosine Metabolism	0.6753	0.6415	0.9968	0.6714	0.8948	1.0032	1.5740	0.9333
phenylacetylglycine	Peptide	Acetylated Peptides	1.1217	1.1187	1.2547	1.1643	0.9040	1.1675	0.9847	0.7909
phenylalanine	Amino Acid	Phenylalanine Metabolism	0.8417	0.8329	0.8184	1.1400	1.1328	1.1444	0.9793	1.1259
phenylalanylalanine	Peptide	Dipeptide	0.2627	0.2260	0.2260	0.5592	1.5278	1.0000	0.4384	0.8096
phenylalanylglycine	Peptide	Dipeptide	0.3396	0.2559	0.1732	0.5503	1.1781	1.0448	0.6950	1.0510
phenyllactate (PLA)	Amino Acid	Phenylalanine Metabolism	1.4684	1.0187	1.0584	1.3845	0.5373	0.3601	0.6558	0.4063
phenylpyruvate	Amino Acid	Phenylalanine Metabolism	0.6183	0.6183	0.6183	0.6953	0.6183	0.6183	1.6317	0.6183
phosphate	Energy	Oxidative Phosphorylation	1.0661	0.9497	0.9450	1.1851	1.3828	1.5104	1.3601	1.5034
phosphoenolpyruvate (PEP)	Carbohydrate	Glycolysis, Gluconeogenesis, and Pyruvate Metabolism	0.3444	0.1506	0.4757	0.6433	11.2684	9.9280	2.6291	2.6845
phosphoethanolamine (PE)	Lipid	Phospholipid Metabolism	0.1014	0.0782	0.1485	0.1081	4.3631	3.6921	4.1164	4.1334
phosphopantetheine	Cofactors and Vitami	Pantothenate and CoA Metabolism	0.3549	0.3685	1.0325	0.8241	0.7198	0.7242	0.8605	0.8216
phytosphingosine	Lipid	Sphingolipid Synthesis	0.7952	0.9950	0.5853	1.3513	0.8567	0.6881	0.5316	0.6749
pipecolate	Amino Acid	Lysine Metabolism	0.9844	1.0100	1.0190	1.1965	0.6508	0.6236	0.8615	0.4574
prolylhydroxyproline	Amino Acid	Urea cycle; Arginine and Proline Metabolism	1.0797	1.0598	1.5206	1.2928	0.3211	0.8103	0.3722	0.3211
proline	Amino Acid	Urea cycle; Arginine and Proline Metabolism	1.5290	1.4047	1.6106	1.6308	0.8561	0.6711	0.5591	1.1180
prolylglycine	Peptide	Dipeptide	0.6173	0.7808	0.7879	1.1923	0.5478	0.6551	0.5849	0.6777
propionylcarnitine (C3)	Lipid	Fatty Acid Metabolism (also BCAA Metabolism)	1.3620	1.1787	1.2403	1.0976	0.9935	1.2600	1.0065	1.3242
pseudouridine	Nucleotide	Pyrimidine Metabolism, Uracil containing	0.9139	0.8849	0.9567	1.0388	1.8328	1.7015	1.8114	2.1116
pterin	Cofactors and Vitami	Pterin Metabolism	1.5619	1.0000	0.4043	1.0256	1.3744	3.0336	1.7835	1.8444
putrescine	Amino Acid	Polyamine Metabolism	4.4138	3.6720	2.6059	4.3463	0.3864	0.2311	0.2311	0.2311
pyridoxal	Cofactors and Vitami	Vitamin B6 Metabolism	1.0101	0.8340	0.9899	0.8510	1.2696	1.5179	1.6791	1.1363
pyridoxal phosphate	Cofactors and Vitami	Vitamin B6 Metabolism	1.1971	0.9953	1.0767	1.1072	1.0047	1.2215	1.2844	0.9079
pyridoxamine	Cofactors and Vitami	Vitamin B6 Metabolism	1.3379	0.9941	0.9725	1.1446	1.9997	1.8804	2.0767	2.0456
pyridoxamine phosphate	Cofactors and Vitami	Vitamin B6 Metabolism	1.0038	0.9552	1.0365	1.1766	2.3982	1.7135	1.7917	2.1537
pyridoxate	Cofactors and Vitami	Vitamin B6 Metabolism	0.3170	0.2992	0.2876	0.3888	1.7736	2.0144	1.7126	1.3896
pyridoxine (Vitamin B6)	Cofactors and Vitami	Vitamin B6 Metabolism	0.8952	0.9364	0.9612	1.0712	1.1303	1.4829	1.2100	1.2083
pyroglutamine*	Amino Acid	Glutamate Metabolism	2.8114	2.3913	2.4864	2.6695	0.8690	0.6995	0.6161	0.9712
pyruvate	Carbohydrate	Glycolysis, Gluconeogenesis, and Pyruvate Metabolism	1.9381	1.7409	1.6286	1.6929	0.2617	0.2676	0.3180	0.3561
quinolinate	Cofactors and Vitami	Nicotinate and Nicotinamide Metabolism	0.4914	0.4914	0.4914	1.0000	0.4914	0.4914	0.4914	0.4914
retinol (Vitamin A)	Cofactors and Vitami	Vitamin A Metabolism	0.5463	0.6087	0.4971	0.8632	5.5673	5.4225	3.8634	4.5288
ribitol	Carbohydrate	Pentose Metabolism	2.1849	1.7066	1.8811	1.6217	0.2873	0.2547	0.2363	0.3460
riboflavin (Vitamin B2)	Cofactors and Vitami	Riboflavin Metabolism	0.7739	0.7090	0.7558	0.9175	0.8382	0.9365	0.8680	0.9415
ribonate	Carbohydrate	Pentose Metabolism	2.0121	1.5414	1.5775	1.4557	0.3857	0.3227	0.3299	0.4549
ribose	Carbohydrate	Pentose Metabolism	0.5015	0.5335	0.5481	0.8238	1.8335	1.4459	0.9465	1.7592
ribulonate/xylulonate/lyxonate*	Carbohydrate	Pentose Metabolism	0.8223	0.7872	0.8064	0.7562	1.3521	3.0350	1.2869	1.1783
ribulose/xylulose	Carbohydrate	Pentose Metabolism	1.3809	1.1231	1.0170	1.1669	0.9830	0.5022	0.5633	0.7666
S-(1,2-dicarboxyethyl)glutathione	Amino Acid	Glutathione Metabolism	1.1226	1.0049	1.1527	1.3600	0.3887	0.3956	0.5162	0.4391
S-1-pyrroline-5-carboxylate	Amino Acid	Glutamate Metabolism	0.4358	1.6502	0.8892	1.5091	0.7282	0.4041	0.3334	0.9236
S-adenosylhomocysteine (SAH)	Amino Acid	Methionine, Cysteine, SAM and Taurine Metabolism	1.3803	1.3704	1.5394	1.6302	0.9293	0.9758	1.0910	0.9384
S-adenosylmethionine (SAM)	Amino Acid	Methionine, Cysteine, SAM and Taurine Metabolism	0.8604	0.8440	0.7454	1.0204	0.6280	0.7824	0.7065	0.7181
S-carboxyethylcysteine	Amino Acid	Methionine, Cysteine, SAM and Taurine Metabolism	0.6493	0.6567	0.6900	0.7847	1.0439	0.9096	1.3111	1.2657
S-methylcysteine sulfoxide	Amino Acid	Methionine, Cysteine, SAM and Taurine Metabolism	0.6303	1.3896	1.1325	0.9672	0.6303	0.6303	0.6303	0.6303
S-methylglutathione	Amino Acid	Glutathione Metabolism	0.8782	0.9495	0.7461	1.3144	0.3605	0.3677	0.3539	0.4135
S-methylmethionine	Amino Acid	Methionine, Cysteine, SAM and Taurine Metabolism	0.6006	0.8666	0.7852	1.0365	0.8621	1.2830	0.9635	0.9302
saccharopine	Amino Acid	Lysine Metabolism	0.2260	0.2225	0.2352	0.6781	1.1723	0.9330	1.6541	1.0078
sarcosine	Amino Acid	Glycine, Serine and Threonine Metabolism	7.5664	5.7365	6.5388	5.9527	0.3858	0.3858	0.3858	0.3858
sedoheptulose-7-phosphate	Carbohydrate	Pentose Phosphate Pathway	0.5104	0.4450	0.3284	0.5407	0.9258	2.5644	3.5440	3.4936
serine	Amino Acid	Glycine, Serine and Threonine Metabolism	0.9251	0.8673	0.7965	1.2268	0.9935	1.0677	1.0065	1.1222
serotonin	Amino Acid	Tryptophan Metabolism	0.2552	0.2891	0.3967	0.5058	1.1923	1.6304	1.1280	1.0326
spermidine	Amino Acid	Polyamine Metabolism	0.8557	0.6762	0.6096	0.7462	0.3430	0.4273	0.2743	0.3401
spermine	Amino Acid	Polyamine Metabolism	1.1962	1.2180	0.8675	1.0308	2.0277	3.0363	1.0188	2.3176
sphingadienine	Lipid	Sphingolipid Synthesis	0.2086	0.2784	0.0753	0.8326	2.0518	2.3605	1.2713	3.0471
sphinganine	Lipid	Sphingolipid Synthesis	0.5556	0.5200	0.3800	0.9931	0.8099	0.5716	0.3493	0.5360
sphingomyelin (d17:1/14:0, d16:1/15:0)*	Lipid	Sphingomyelins	0.8447	1.1780	1.0507	1.0201	0.7171	0.4692	0.2675	0.3985
sphingomyelin (d17:1/16:0, d18:1/15:0, d16:1/1	Lipid	Sphingomyelins	0.7974	0.9649	0.8361	0.9542	1.6953	1.0117	0.7184	0.8459
sphingomyelin (d17:2/16:0, d18:2/15:0)*	Lipid	Sphingomyelins	0.8285	1.1072	0.9421	0.8787	1.3824	1.0000	0.7889	0.7889
sphingomyelin (d18:0/18:0, d19:0/17:0)*	Lipid	Dihydrosphingomyelins	0.3830	0.5536	0.3230	0.9669	3.8199	2.2561	1.0331	1.3929
sphingomyelin (d18:0/20:0, d16:0/22:0)*	Lipid	Dihydrosphingomyelins	0.6629	0.3731	0.4212	1.0518	2.3261	1.6246	0.7300	1.0710
sphingomyelin (d18:1/14:0, d16:1/16:0)*	Lipid	Sphingomyelins	0.8456	0.9827	0.8398	0.9397	1.4776	1.0173	0.6966	0.7529
sphingomyelin (d18:1/17:0, d17:1/18:0, d19:1/1	Lipid	Sphingomyelins	0.5764	0.6841	0.5616	0.7526	3.7016	2.6218	1.7746	1.9568
sphingomyelin (d18:1/18:1, d18:2/18:0)	Lipid	Sphingomyelins	0.8870	1.0126	0.9103	1.0785	3.7901	3.1547	2.3919	2.2179
sphingomyelin (d18:1/20:0, d16:1/22:0)*	Lipid	Sphingomyelins	0.6056	0.6945	0.4523	0.7551	5.2533	3.1266	2.4279	2.6233
sphingomyelin (d18:1/21:0, d17:1/22:0, d16:1/2	Lipid	Sphingomyelins	0.5642	0.7268	0.5746	0.7256	2.7718	1.3121	0.7337	0.9683
sphingomyelin (d18:1/22:1, d18:2/22:0, d16:1/2	Lipid	Sphingomyelins	0.7577	0.8761	0.6593	0.9317	2.5292	1.4390	0.9960	1.1316
sphingomyelin (d18:1/22:2, d18:2/22:1, d16:1/2	Lipid	Sphingomyelins	0.6750	0.8453	0.8220	0.8907	2.2167	1.4872	0.9414	1.3041
sphingomyelin (d18:1/24:1, d18:2/24:0)*	Lipid	Sphingomyelins	0.6835	0.9993	0.8500	0.9166	3.8686	2.2624	1.1399	1.5808
sphingomyelin (d18:2/14:0, d18:1/14:1)*	Lipid	Sphingomyelins	1.0093	1.3103	1.2388	1.0296	0.8339	0.5402	0.3796	0.4708
sphingomyelin (d18:2/16:0, d18:1/16:1)*	Lipid	Sphingomyelins	0.8686	0.9847	0.8296	0.9848	1.5218	1.0152	0.7030	0.7958
sphingomyelin (d18:2/23:0, d18:1/23:1, d17:1/2	Lipid	Sphingomyelins	0.7437	0.9601	0.7586	0.8952	3.4842	1.7964	0.9573	1.3527
sphingomyelin (d18:2/23:1)*	Lipid	Sphingomyelins	0.6596	0.7626	0.8219	0.7823	2.6412	1.5151	0.8797	1.1113
sphingomyelin (d18:2/24:1, d18:1/24:2)*	Lipid	Sphingomyelins	0.8721	1.0650	0.8799	0.9982	3.2938	2.0939	1.3248	1.5080
sphingomyelin (d18:2/24:2)*	Lipid	Sphingomyelins	0.8837	1.0127	0.9312	1.2832	2.7659	1.0424	1.2194	1.3956
sphingosine	Lipid	Sphingosines	0.4280	0.5087	0.2156	0.9862	2.0302	1.4848	0.7642	1.5380
sphingosine 1-phosphate	Lipid	Sphingosines	0.4881	0.4881	0.4881	0.4881	0.4881	0.4881	0.4881	0.6218
stachydrine	Xenobiotics	Food Component/Plant	1.5003	1.5355	1.7555	1.4751	1.0582	0.9195	0.8408	0.7829
stearate (18:0)	Lipid	Long Chain Saturated Fatty Acid	0.7553	0.6134	1.5214	0.8446	4.0682	5.0039	3.7403	2.7531
stearoyl ethanolamide	Lipid	Endocannabinoid	0.6080	0.7589	0.6623	0.8400	1.7487	1.0741	0.7521	1.0053
stearoyl sphingomyelin (d18:1/18:0)	Lipid	Sphingomyelins	0.7367	0.8128	0.5980	1.0126	4.4286	4.2925	3.3284	3.1126
stearoylcarnitine (C18)	Lipid	Fatty Acid Metabolism (Acyl Carnitine, Long Chain Saturated)	0.8608	0.8638	1.9176	0.7532	0.8700	1.2017	0.5727	1.1300
succinate	Energy	TCA Cycle	1.2888	1.1122	1.0952	1.0359	0.6069	0.8642	0.8518	0.7364
succinylcarnitine (C4-DC)	Energy	TCA Cycle	0.8414	0.6962	0.9662	0.8790	1.8235	2.4264	2.2817	2.2133
sulfate*	Xenobiotics	Chemical	1.8180	0.9921	0.4200	0.7710	6.6371	8.5433	5.5402	4.4835
taurine	Amino Acid	Methionine, Cysteine, SAM and Taurine Metabolism	1.3521	1.1750	1.1123	1.3817	1.8637	1.0397	0.9603	2.1666
taurochenodeoxycholate	Lipid	Primary Bile Acid Metabolism	0.6233	0.6233	0.6233	0.6233	0.6233	0.6233	0.6233	0.6233
thiamin (Vitamin B1)	Cofactors and Vitami	Thiamine Metabolism	0.2406	0.2340	0.2989	0.4362	1.1177	1.1846	1.0699	1.0971
thiamin diphosphate	Cofactors and Vitami	Thiamine Metabolism	0.5226	0.7658	0.9018	0.9030	1.9322	1.5698	1.6546	0.5226
thiamin monophosphate	Cofactors and Vitami	Thiamine Metabolism	0.3552	0.3552	0.9875	0.3552	9.3097	11.3994	11.4923	10.8483
thioproline	Xenobiotics	Chemical	0.8700	0.8099	0.8017	1.0410	1.6078	1.3899	1.2605	1.4864
threonate	Cofactors and Vitami	Ascorbate and Aldarate Metabolism	0.7151	0.7151	0.7151	0.7151	1.0303	1.2383	0.9429	0.7151
threonine	Amino Acid	Glycine, Serine and Threonine Metabolism	0.6490	0.6676	0.7021	1.0025	0.6959	0.6344	0.5608	0.7011
threonylphenylalanine	Peptide	Dipeptide	0.4746	0.4746	0.4746	0.4746	0.4746	0.4746	0.4746	0.4746
thymidine	Nucleotide	Pyrimidine Metabolism, Thymine containing	0.5487	0.5952	0.8342	1.0713	1.2029	0.4957	0.6184	0.8139
TMP	Nucleotide	Pyrimidine Metabolism, Thymine containing	1.7760	1.8858	1.4679	2.6894	0.2062	0.1554	0.1467	0.1935
thymine	Nucleotide	Pyrimidine Metabolism, Thymine containing	0.9247	0.5692	1.2962	1.3025	0.5676	0.5676	0.5676	0.5676
tiglyl carnitine (C5)	Amino Acid	Leucine, Isoleucine and Valine Metabolism	0.7257	0.7015	0.9115	0.8148	1.7085	2.0201	1.9629	2.6721
hydroxyproline	Amino Acid	Urea cycle; Arginine and Proline Metabolism	0.7455	0.8297	0.8273	1.1939	0.8211	0.7971	0.7403	0.8149
trans-urocanate	Amino Acid	Histidine Metabolism	1.0888	0.9193	0.6820	1.1869	1.5146	0.8704	1.3892	1.3593
tricosanoyl sphingomyelin (d18:1/23:0)*	Lipid	Sphingomyelins	0.8191	0.9038	0.8252	0.9830	3.1201	1.7622	0.9829	1.0776
trigonelline (N'-methylnicotinate)	Cofactors and Vitami	Nicotinate and Nicotinamide Metabolism	1.0328	0.9771	1.2672	1.0229	0.9020	0.6826	0.7222	1.4428
trimethylamine N-oxide	Lipid	Phospholipid Metabolism	1.6213	1.6130	1.9607	1.5535	0.8954	1.0489	0.8935	0.8923
tryptamine	Amino Acid	Tryptophan Metabolism	0.6945	0.6945	0.6945	0.6945	1.1705	0.8269	0.6945	1.1602

tryptophan	Amino Acid	Tryptophan Metabolism	0.7634	0.7744	0.7696	1.1199	0.8504	0.8730	0.7682	0.9327
tryptophan betaine	Amino Acid	Tryptophan Metabolism	1.1572	1.0000	1.1357	1.0946	0.2660	0.2660	0.2660	0.2995
tryptophylglycine	Peptide	Dipeptide	0.4913	0.4269	0.4089	0.6532	0.9176	0.7249	0.4089	0.9422
tyrosine	Amino Acid	Tyrosine Metabolism	0.7428	0.7439	0.6785	1.2142	1.1971	1.1063	0.9966	1.1725
tyrosylglycine	Peptide	Dipeptide	0.3020	0.3577	0.1668	0.8011	1.0795	0.7305	0.5542	0.8897
UDP-galactose	Carbohydrate	Nucleotide Sugar	1.4833	1.2556	0.9773	1.4871	0.9594	1.0227	1.3475	0.8897
UDP-glucose	Carbohydrate	Nucleotide Sugar	2.2758	1.9276	1.4752	2.1006	0.7060	0.8257	0.9084	0.6013
UDP-glucuronate	Carbohydrate	Nucleotide Sugar	0.5481	0.5419	0.6518	0.6964	0.8791	1.0095	0.9438	0.8863
UDP-N-acetylglucosamine/galactosamine	Carbohydrate	Nucleotide Sugar	0.7456	0.5846	0.5611	0.9158	0.9204	1.3037	1.4391	1.3503
uracil	Nucleotide	Pyrimidine Metabolism, Uracil containing	0.9845	1.5594	2.7868	1.8673	0.4900	0.4654	0.9297	1.0155
urate	Nucleotide	Purine Metabolism, (Hypo)Xanthine/Inosine containing	1.2086	1.1335	1.1628	1.3200	1.4435	1.7119	1.4444	1.2252
uridine	Nucleotide	Pyrimidine Metabolism, Uracil containing	0.8877	1.0307	1.4773	1.3380	1.3190	1.3568	1.4126	1.5797
uridine 3'-monophosphate (3'-UMP)	Nucleotide	Pyrimidine Metabolism, Uracil containing	0.3166	0.3166	0.3166	0.3166	1.2493	0.6674	0.9163	2.0880
uridine 5'-diphosphate (UDP)	Nucleotide	Pyrimidine Metabolism, Uracil containing	1.7333	1.0561	0.5930	1.1123	3.8711	5.6883	2.8705	2.4162
UMP	Nucleotide	Pyrimidine Metabolism, Uracil containing	1.6464	1.2562	0.8078	1.6313	0.8684	0.8357	0.9928	1.1826
uridine 5'-triphosphate (UTP)	Nucleotide	Pyrimidine Metabolism, Uracil containing	1.1051	0.6693	0.2703	0.4009	10.7940	13.0267	3.0500	3.5430
valine	Amino Acid	Leucine, Isoleucine and Valine Metabolism	0.8188	0.8404	0.8592	1.0913	0.9232	0.9987	0.8415	0.9747
valylglutamine	Peptide	Dipeptide	0.5572	0.5572	0.5572	0.5572	0.8353	0.5572	0.5789	0.5572
valylglycine	Peptide	Dipeptide	0.2432	0.2244	0.1254	0.5227	1.4869	0.7190	0.4563	0.7273
valylleucine	Peptide	Dipeptide	0.2307	0.2307	0.2307	0.2307	0.8286	0.4182	0.2307	0.2307
xanthine	Nucleotide	Purine Metabolism, (Hypo)Xanthine/Inosine containing	0.7572	0.8766	1.3643	1.2304	0.9495	0.9157	1.2128	1.1575
xanthosine	Nucleotide	Purine Metabolism, (Hypo)Xanthine/Inosine containing	0.3498	0.5259	1.0934	0.8221	1.0340	1.2937	2.1802	1.2756

Table S2: Metabolites that are significantly altered in persister cells.

Cell Line: **A375**
Treatment: **Vemurafenib (10 µM)**
Tratement duration: **3 days**

Pathway Heat Map ***This is the heat map associated with the statistical analysis of the data. Indicates ratios, p- and q-values for each comparison.***

0.55	**Green:** indicates significant difference ($p \leq 0.05$) between the groups shown, metabolite ratio of < 1.00
0.76	**Light Green:** narrowly missed statistical cutoff for significance 0.05<p<0.10, metabolite ratio of < 1.00
1.71	**Red:** indicates significant difference ($p \leq 0.05$) between the groups shown; metabolite ratio of ≥ 1.00
1.32	**Light Red:** narrowly missed statistical cutoff for significance 0.05<p<0.10, metabolite ratio of ≥ 1.00
1.20	**Non-colored text and cell:** mean values are not significantly different for that comparison

			Fold of Change (VEM/Control)	Statistical Values	
Super Pathway	Sub Pathway	Biochemical Name	Vemurafenib (VEM)	p-value	q-value
	Glycine, Serine and Threonine Metabolism	glycine	0.57	0.000	0.000
		sarcosine	0.06	0.000	0.000
		dimethylglycine	0.73	0.005	0.003
		betaine	0.66	0.000	0.000
		serine	1.10	0.237	0.085
		N-acetylserine	0.24	0.000	0.000
		threonine	0.86	0.174	0.064
		N-acetylthreonine	0.37	0.000	0.000
	Alanine and Aspartate Metabolism	alanine	0.87	0.160	0.060
		N-acetylalanine	0.35	0.000	0.000
		aspartate	1.77	0.000	0.000
		N-acetylaspartate (NAA)	0.62	0.000	0.000
		asparagine	1.17	0.080	0.032
		N-acetylasparagine	0.83	0.169	0.063
		hydroxyasparagine**	0.39	0.001	0.000
	Glutamate Metabolism	glutamate	1.03	0.589	0.187
		glutamine	0.61	0.003	0.002
		alpha-ketoglutaramate*	0.34	0.004	0.003
		N-acetylglutamate	0.24	0.000	0.000
		N-acetylglutamine	0.36	0.000	0.000
		4-hydroxyglutamate	0.24	0.000	0.000
		glutamate, gamma-methyl ester	0.34	0.000	0.000
		pyroglutamine*	0.30	0.000	0.000
		N-acetyl-aspartyl-glutamate (NAAG)	0.77	0.022	0.010
		beta-citrylglutamate	1.11	0.183	0.067
		carboxyethyl-GABA	9.71	0.000	0.000
		S-1-pyrroline-5-carboxylate	0.53	0.090	0.036
	Histidine Metabolism	histidine	1.34	0.007	0.004
		1-methylhistidine	1.15	0.267	0.094
		3-methylhistidine	0.40	0.000	0.000
		N-acetylhistidine	8.13	0.000	0.000
		N-acetyl-3-methylhistidine*	8.46	0.000	0.000
		N-acetyl-1-methylhistidine*	0.76	0.270	0.095
		trans-urocanate	1.32	0.193	0.071
		imidazole propionate	0.05	0.000	0.000
		formiminoglutamate	1.57	0.048	0.021
		imidazole lactate	0.06	0.000	0.000
		carnosine	1.49	0.001	0.001
		1-methyl-4-imidazoleacetate	0.75	0.011	0.006
		1-methyl-5-imidazoleacetate	0.09	0.000	0.000
		1-ribosyl-imidazoleacetate*	0.41	0.000	0.000
		4-imidazoleacetate	0.27	0.000	0.000
		histidine methyl ester	0.91	0.338	0.116
	Lysine Metabolism	lysine	1.83	0.000	0.000
		N2-acetyllysine	6.64	0.000	0.000
		N6-acetyllysine	0.94	0.568	0.182
		N6-methyllysine	1.63	0.000	0.000
		N6,N6-dimethyllysine	0.95	0.450	0.148
		N6,N6,N6-trimethyllysine	0.96	0.667	0.208
		hydroxy-N6,N6,N6-trimethyllysine*	0.93	0.500	0.163
		5-hydroxylysine	2.34	0.000	0.000
		5-(galactosylhydroxy)-L-lysine	4.20	0.000	0.000
		fructosyllysine	1.41	0.288	0.100
		saccharopine	3.50	0.000	0.000
		2-aminoadipate	2.29	0.002	0.002
		glutarylcarnitine (C5-DC)	9.63	0.000	0.000
		pipecolate	0.62	0.000	0.000
		6-oxopiperidine-2-carboxylate	0.70	0.004	0.002
		cadaverine	0.87	0.006	0.004
		N-acetyl-cadaverine	0.24	0.007	0.004
		5-aminovalerate	0.39	0.000	0.000
		N,N,N-trimethyl-5-aminovalerate	0.99	0.896	0.270
	Phenylalanine Metabolism	phenylalanine	1.21	0.021	0.010
		N-acetylphenylalanine	0.50	0.000	0.000
		1-carboxyethylphenylalanine	0.23	0.000	0.000
		phenylpyruvate	1.37	0.395	0.132
		phenyllactate (PLA)	0.40	0.000	0.000
		phenethylamine	5.48	0.000	0.000
		tyrosine	1.32	0.016	0.008
		N-acetyltyrosine	1.53	0.000	0.000
		1-carboxyethyltyrosine	0.09	0.000	0.000

Amino Acid	Tyrosine Metabolism	4-hydroxyphenylpyruvate	2.24	0.000	0.000
		3-(4-hydroxyphenyl)lactate	0.29	0.000	0.000
		phenol sulfate	1.48	0.038	0.017
		3-methoxytyrosine	0.69	0.063	0.026
		o-Tyrosine	1.57	0.345	0.117
		O-methyltyrosine	0.78	0.009	0.005
	Tryptophan Metabolism	tryptophan	1.00	0.902	0.271
		N-acetyltryptophan	1.00	1.000	0.291
		C-glycosyltryptophan	4.42	0.000	0.000
		tryptophan betaine	0.25	0.000	0.000
		kynurenine	0.81	0.051	0.022
		kynurenate	4.88	0.000	0.000
		serotonin	3.44	0.000	0.000
		tryptamine	1.39	0.244	0.087
		indolelactate	0.61	0.033	0.015
		indoleacetate	1.92	0.006	0.003
	Leucine, Isoleucine and Valine Metabolism	leucine	1.28	0.004	0.002
		N-acetylleucine	0.48	0.000	0.000
		1-carboxyethylleucine	0.41	0.000	0.000
		4-methyl-2-oxopentanoate	0.47	0.000	0.000
		alpha-hydroxyisocaproate	0.45	0.000	0.000
		isovalerylglycine	1.19	0.762	0.234
		isovalerylcarnitine (C5)	1.56	0.018	0.008
		beta-hydroxyisovalerate	0.32	0.000	0.000
		beta-hydroxyisovaleroylcarnitine	2.84	0.000	0.000
		3-methylglutaconate	1.08	0.391	0.131
		isoleucine	1.26	0.004	0.003
		N-acetylisoleucine	0.46	0.005	0.003
		1-carboxyethylisoleucine	0.14	0.000	0.000
		3-methyl-2-oxovalerate	0.41	0.006	0.003
		2-hydroxy-3-methylvalerate	0.38	0.000	0.000
		2-methylbutyrylcarnitine (C5)	1.54	0.008	0.004
		2-methylbutyrylglycine	0.75	0.114	0.044
		tiglylcarnitine (C5:1-DC)	2.65	0.000	0.000
		3-hydroxy-2-ethylpropionate	0.99	0.976	0.290
		ethylmalonate	0.13	0.000	0.000
		methylsuccinate	0.77	0.048	0.021
		valine	1.04	0.573	0.183
		N-acetylvaline	0.60	0.000	0.000
		1-carboxyethylvaline	0.14	0.000	0.000
		3-methyl-2-oxobutyrate	0.45	0.005	0.003
		alpha-hydroxyisovalerate	0.23	0.000	0.000
		isobutyrylcarnitine (C4)	1.99	0.000	0.000
		3-hydroxyisobutyrate	0.96	0.822	0.251
		2,3-dihydroxy-2-methylbutyrate	0.35	0.000	0.000
	Methionine, Cysteine, SAM and Taurine Metabolism	methionine	1.26	0.007	0.004
		N-acetylmethionine	1.72	0.001	0.001
		N-formylmethionine	1.03	0.759	0.233
		S-methylmethionine	1.23	0.370	0.125
		methionine sulfone	0.41	0.000	0.000
		methionine sulfoxide	1.71	0.002	0.001
		N-acetylmethionine sulfoxide	5.35	0.000	0.000
		S-adenosylmethionine (SAM)	0.82	0.037	0.016
		S-adenosylhomocysteine (SAH)	0.66	0.000	0.000
		2,3-dihydroxy-5-methylthio-4-pentenoate (DMTPA)*	0.61	0.000	0.000
		homocysteine	0.64	0.023	0.011
		cystathionine	0.15	0.000	0.000
		cysteine	1.06	0.591	0.188
		N-acetylcysteine	1.69	0.010	0.005
		S-methylcysteine sulfoxide	0.61	0.041	0.018
		S-carboxyethylcysteine	1.63	0.001	0.001
		hypotaurine	0.93	0.437	0.145
		taurine	1.20	0.441	0.145
		N-acetyltaurine	0.34	0.000	0.000
	Urea cycle; Arginine and Proline Metabolism	arginine	1.97	0.000	0.000
		argininosuccinate	0.66	0.081	0.033
		ornithine	1.75	0.017	0.008
		3-amino-2-piperidone	4.08	0.000	0.000
		2-oxoarginine*	1.25	0.096	0.038
		citrulline	0.78	0.012	0.006
		proline	0.52	0.000	0.000
		dimethylarginine (SDMA + ADMA)	1.58	0.004	0.002
		N-acetylarginine	1.31	0.002	0.001
		N-delta-acetylornithine	0.54	0.000	0.000
		trans-4-hydroxyproline	0.88	0.243	0.087
		pro-hydroxy-pro	0.37	0.001	0.000
		N-methylproline	0.73	0.047	0.020
		N,N,N-trimethyl-alanylproline betaine (TMAP)	5.00	0.000	0.000
		N-monomethylarginine	3.09	0.000	0.000
	Creatine Metabolism	creatine	1.22	0.006	0.004
		creatinine	1.29	0.010	0.005
		creatine phosphate	0.72	0.010	0.005
		putrescine	0.07	0.000	0.000
		N-acetylputrescine	0.31	0.007	0.004
		N-acetyl-isoputreanine	4.13	0.000	0.000
		spermidine	0.48	0.000	0.000

	Polyamine Metabolism	N('1)-acetylspermidine	0.12	0.000	0.000
		diacetylspermidine*	1.00	1.000	0.291
		spermine	1.95	0.077	0.031
		N(1)-acetylspermine	0.82	0.155	0.058
		N1,N12-diacetylspermine	1.00	1.000	0.291
		5-methylthioadenosine (MTA)	0.83	0.021	0.010
		4-acetamidobutanoate	1.45	0.012	0.006
	Guanidino and Acetamido Metabolism	1-methylguanidine	1.00	0.985	0.291
		4-guanidinobutanoate	0.27	0.000	0.000
	Glutathione Metabolism	glutathione, reduced (GSH)	1.22	0.048	0.021
		glutathione, oxidized (GSSG)	1.07	0.440	0.145
		cyclic dGSH	0.84	0.060	0.025
		cysteine-glutathione disulfide	1.48	0.124	0.048
		S-methylglutathione	0.38	0.000	0.000
		cysteinylglycine	0.64	0.000	0.000
		5-oxoproline	0.84	0.025	0.012
		2-hydroxybutyrate/2-hydroxyisobutyrate	0.73	0.000	0.000
		ophthalmate	9.86	0.000	0.000
		S-(1,2-dicarboxyethyl)glutathione	0.37	0.000	0.000
		4-hydroxy-nonenal-glutathione	2.11	0.000	0.000
		CoA-glutathione*	2.53	0.000	0.000
Peptide	Gamma-glutamyl Amino Acid	gamma-glutamylcysteine	0.72	0.010	0.005
		gamma-glutamylglutamate	1.39	0.019	0.009
		gamma-glutamylglutamine	5.98	0.000	0.000
		gamma-glutamylhistidine	1.69	0.064	0.027
		gamma-glutamylisoleucine*	3.02	0.000	0.000
		gamma-glutamylleucine	4.72	0.000	0.000
		gamma-glutamylmethionine	2.55	0.000	0.000
		gamma-glutamylphenylalanine	0.83	0.100	0.039
		gamma-glutamylthreonine	10.50	0.000	0.000
		gamma-glutamyltryptophan	1.00	1.000	0.291
		gamma-glutamyltyrosine	0.77	0.209	0.076
		gamma-glutamylvaline	4.97	0.000	0.000
	Dipeptide	alanylleucine	1.29	0.291	0.101
		glycylisoleucine	0.64	0.131	0.050
		glycylleucine	1.44	0.085	0.034
		glycylvaline	1.20	0.343	0.117
		isoleucylglycine	2.98	0.001	0.000
		leucylglycine	2.67	0.006	0.003
		phenylalanylalanine	2.96	0.001	0.001
		phenylalanylglycine	3.01	0.000	0.000
		prolylglycine	0.73	0.034	0.015
		threonylphenylalanine	1.00	1.000	0.291
		tryptophylglycine	1.51	0.085	0.034
		tyrosylglycine	2.00	0.010	0.005
		valylglutamine	1.13	0.472	0.155
		valylglycine	3.04	0.002	0.001
		valylleucine	1.85	0.096	0.038
		leucylglutamine*	2.15	0.004	0.002
	Acetylated Peptides	phenylacetylglycine	0.83	0.401	0.133
Carbohydrate	Glycolysis, Gluconeogenesis, and Pyruvate Metabolism	glucose	51.27	0.011	0.006
		glucose 6-phosphate	30.81	0.000	0.000
		fructose 1,6-diphosphate/glucose 1,6-diphosphate/myo-ir	11.39	0.000	0.000
		dihydroxyacetone phosphate (DHAP)	2.35	0.866	0.263
		3-phosphoglycerate	0.91	0.592	0.188
		phosphoenolpyruvate (PEP)	16.43	0.000	0.000
		pyruvate	0.17	0.000	0.000
		lactate	1.06	0.520	0.169
		glycerate	5.03	0.000	0.000
	Pentose Phosphate Pathway	6-phosphogluconate	24.28	0.000	0.000
		sedoheptulose-7-phosphate	5.77	0.000	0.000
	Pentose Metabolism	ribose	2.49	0.001	0.001
		ribitol	0.15	0.000	0.000
		ribonate	0.23	0.000	0.000
		ribulose/xylulose	0.60	0.002	0.002
		arabinose	3.10	0.000	0.000
		arabitol/xylitol	0.94	0.504	0.164
		arabonate/xylonate	1.62	0.100	0.039
		ribulonate/xylulonate/lyxonate*	2.16	0.003	0.002
	Disaccharides and Oligosaccharides	lactose	9.93	0.000	0.000
	Fructose, Mannose and Galactose Metabolism	fructose	18.60	0.000	0.000
		mannitol/sorbitol	0.57	0.002	0.001
		mannose	9.42	0.262	0.093
		galactitol (dulcitol)	3.06	0.000	0.000
		galactonate	0.24	0.000	0.000
	Nucleotide Sugar	adenosine-5'-diphosphoglucose	7.34	0.000	0.000
		UDP-glucose	0.39	0.000	0.000
		UDP-galactose	0.81	0.092	0.036
		UDP-glucuronate	1.53	0.000	0.000
		guanosine 5'-diphospho-fucose	1.49	0.001	0.000
		UDP-N-acetylglucosamine/galactosamine	1.79	0.001	0.001
		cytidine 5'-monophospho-N-acetylneuraminic acid	0.71	0.003	0.002
		glucosamine-6-phosphate	118.21	0.000	0.000
		glucuronate	1.17	0.265	0.094
		N-acetylglucosamine 6-phosphate	7.44	0.000	0.000
		N-acetyl-glucosamine 1-phosphate	0.47	0.000	0.000

	Aminosugar Metabolism	N-acetylneuraminate	0.10	0.000	0.000
		N-acetylglucosaminylasparagine	0.89	0.078	0.032
		erythronate*	0.19	0.000	0.000
		N-acetylglucosamine/N-acetylgalactosamine	5.26	0.000	0.000
		N-glycolylneuraminate	0.09	0.000	0.000
	Advanced Glycation End-product	N6-carboxymethyllysine	0.99	0.611	0.193
Energy	TCA Cycle	citrate	2.29	0.000	0.000
		aconitate [cis or trans]	3.80	0.000	0.000
		isocitrate	13.41	0.000	0.000
		alpha-ketoglutarate	0.38	0.000	0.000
		succinylcarnitine (C4-DC)	2.59	0.000	0.000
		succinate	0.68	0.000	0.000
		fumarate	0.91	0.383	0.129
		malate	1.12	0.187	0.069
		oxaloacetate	0.84	0.534	0.173
		2-methylcitrate/homocitrate	2.66	0.000	0.000
	Oxidative Phosphorylation	acetylphosphate	10.20	0.066	0.027
		phosphate	1.39	0.001	0.000
	Fatty Acid Synthesis	malonylcarnitine	6.35	0.000	0.000
	Fatty Acid Metabolism	acetyl CoA	3.62	0.001	0.001
		oleoyl CoA	1.85	0.014	0.007
		arachidonoyl CoA	2.47	0.002	0.001
	Short Chain Fatty Acid	butyrate/isobutyrate (4:0)	2.83	0.082	0.033
	Medium Chain Fatty Acid	heptanoate (7:0)	1.80	0.000	0.000
		(2 or 3)-decenoate (10:1n7 or n8)	1.43	0.181	0.067
		5-dodecenoate (12:1n7)	0.64	0.118	0.046
	Long Chain Saturated Fatty Acid	myristate (14:0)	1.72	0.341	0.117
		pentadecanoate (15:0)	3.53	0.035	0.015
		palmitate (16:0)	3.06	0.017	0.008
		margarate (17:0)	6.14	0.014	0.007
		stearate (18:0)	4.17	0.006	0.004
		nonadecanoate (19:0)	4.98	0.031	0.014
		arachidate (20:0)	2.57	0.144	0.054
	Long Chain Monounsaturated Fatty Acid	myristoleate (14:1n5)	1.14	0.676	0.211
		palmitoleate (16:1n7)	3.07	0.120	0.046
		10-heptadecenoate (17:1n7)	8.44	0.015	0.007
		oleate/vaccenate (18:1)	6.40	0.013	0.006
		10-nonadecenoate (19:1n9)	8.64	0.011	0.006
		eicosenoate (20:1)	4.16	0.057	0.024
		erucate (22:1n9)	2.63	0.139	0.053
	Long Chain Polyunsaturated Fatty Acid (n3 and n6)	eicosapentaenoate (EPA; 20:5n3)	34.92	0.003	0.002
		heneicosapentaenoate (21:5n3)	21.96	0.001	0.001
		docosapentaenoate (n3 DPA; 22:5n3)	35.48	0.002	0.001
		docosahexaenoate (DHA; 22:6n3)	13.02	0.009	0.005
		docosatrienoate (22:3n3)	7.69	0.030	0.014
		nisinate (24:6n3)	2.90	0.161	0.060
		hexadecadienoate (16:2n6)	6.56	0.006	0.003
		linoleate (18:2n6)	10.78	0.007	0.004
		linolenate [alpha or gamma; (18:3n3 or 6)]	2.75	0.099	0.039
		dihomo-linoleate (20:2n6)	16.02	0.003	0.002
		dihomo-linolenate (20:3n3 or n6)	23.01	0.001	0.001
		arachidonate (20:4n6)	50.75	0.001	0.001
		docosatrienoate (22:3n6)*	11.09	0.006	0.004
		docosapentaenoate (n6 DPA; 22:5n6)	17.59	0.002	0.001
		docosadienoate (22:2n6)	2.86	0.121	0.047
		mead acid (20:3n9)	20.09	0.002	0.001
	Fatty Acid, Branched	(12 or 13)-methylmyristate (a15:0 or i15:0)	2.24	0.107	0.042
		(14 or 15)-methylpalmitate (a17:0 or i17:0)	4.69	0.025	0.012
		(16 or 17)-methylstearate (a19:0 or i19:0)	4.11	0.050	0.021
	Fatty Acid, Dicarboxylate	dimethylmalonic acid	1.26	0.615	0.194
		glutarate (C5-DC)	0.96	0.540	0.175
		2-hydroxyglutarate	0.55	0.001	0.001
		2-hydroxyadipate	0.65	0.316	0.109
		3-hydroxyadipate*	1.28	0.137	0.052
		maleate	0.84	0.198	0.072
		dodecadienoate (12:2)*	2.22	0.011	0.005
	Fatty Acid Metabolism (also BCAA Metabolism)	butyrylcarnitine (C4)	0.61	0.001	0.001
		propionylcarnitine (C3)	0.94	0.479	0.157
		methylmalonate (MMA)	2.20	0.001	0.001
	Fatty Acid Metabolism (Acyl Glycine)	N-palmitoylglycine	0.18	0.035	0.015
	Fatty Acid Metabolism (Acyl Carnitine, Short Chain)	acetylcarnitine (C2)	1.05	0.940	0.281
	Fatty Acid Metabolism (Acyl Carnitine, Medium Chain)	hexanoylcarnitine (C6)	1.23	0.195	0.072
		octanoylcarnitine (C8)	1.37	0.221	0.080
		decanoylcarnitine (C10)	0.63	0.149	0.056
		laurylcarnitine (C12)	0.41	0.002	0.001
	atty Acid Metabolism (Acyl Carnitine, Long Chain Saturated	myristoylcarnitine (C14)	0.26	0.000	0.000
		pentadecanoylcarnitine (C15)*	0.69	0.112	0.044
		palmitoylcarnitine (C16)	0.40	0.001	0.001
		margaroylcarnitine (C17)*	0.94	0.880	0.266
		stearoylcarnitine (C18)	0.86	0.611	0.193
		arachidoylcarnitine (C20)*	0.70	0.312	0.108
	Fatty Acid Metabolism (Acyl Carnitine, Monounsaturated)	cis-4-decenoylcarnitine (C10:1)	1.17	0.895	0.270
		5-dodecenoylcarnitine (C12:1)	0.70	0.157	0.059
		myristoleoylcarnitine (C14:1)*	0.94	0.916	0.275
		palmitoleoylcarnitine (C16:1)*	0.50	0.018	0.009
		oleoylcarnitine (C18:1)	0.69	0.165	0.062

		eicosenoylcarnitine (C20:1)*	0.50	0.031	0.014
		erucoylcarnitine (C22:1)*	0.80	0.316	0.109
	Fatty Acid Metabolism (Acyl Carnitine, Polyunsaturated)	linoleoylcarnitine (C18:2)*	1.56	0.147	0.056
		linolenoylcarnitine (C18:3)*	0.47	0.006	0.003
		dihomo-linoleoylcarnitine (C20:2)*	0.91	0.723	0.224
		arachidonoylcarnitine (C20:4)	1.25	0.552	0.178
		dihomo-linolenoylcarnitine (C20:3n3 or 6)*	1.40	0.327	0.113
		adrenoylcarnitine (C22:4)*	1.33	0.550	0.177
		docosapentaenoylcarnitine (C22:5n3)*	1.20	0.747	0.231
		docosahexaenoylcarnitine (C22:6)*	0.35	0.045	0.020
	Fatty Acid Metabolism (Acyl Carnitine, Hydroxy)	(R)-3-hydroxybutyrylcarnitine	1.68	0.000	0.000
		(S)-3-hydroxybutyrylcarnitine	0.87	0.229	0.082
		3-hydroxyhexanoylcarnitine (1)	3.41	0.000	0.000
		3-hydroxydecanoylcarnitine	1.75	0.029	0.013
		3-hydroxypalmitoylcarnitine	0.61	0.054	0.023
		3-hydroxyoleoylcarnitine	1.30	0.339	0.116
	Carnitine Metabolism	deoxycarnitine	0.92	0.332	0.114
		carnitine	1.72	0.002	0.001
	Ketone Bodies	3-hydroxybutyrate (BHBA)	1.58	0.004	0.002
	Fatty Acid Metabolism (Acyl Choline)	palmitoylcholine	3.00	0.008	0.004
		oleoylcholine	4.46	0.000	0.000
		palmitoloelycholine	3.24	0.002	0.001
		linoleoylcholine*	2.64	0.002	0.002
		docosahexaenoylcholine	7.30	0.000	0.000
		arachidonoylcholine	1.00	1.000	0.291
	Fatty Acid, Monohydroxy	4-hydroxybutyrate (GHB)	0.84	0.216	0.078
		2-hydroxypalmitate	2.86	0.093	0.037
		2-hydroxystearate	3.27	0.067	0.028
		3-hydroxyhexanoate	1.01	0.902	0.271
		3-hydroxyoctanoate	1.01	0.937	0.281
		3-hydroxydecanoate	1.21	0.445	0.146
		3-hydroxytridecanoate	2.61	0.009	0.005
		3-hydroxylaurate	1.11	0.648	0.203
		3-hydroxymyristate	1.51	0.357	0.121
		3-hydroxypalmitate	2.20	0.236	0.085
		3-hydroxystearate	1.99	0.222	0.080
		3-hydroxyoleate*	4.11	0.077	0.031
		9-hydroxystearate	0.50	0.433	0.144
	Fatty Acid, Dihydroxy	2S,3R-dihydroxybutyrate	0.57	0.202	0.074
		2R,3R-dihydroxybutyrate	0.61	0.010	0.005
		2,4-dihydroxybutyrate	0.39	0.000	0.000
	Endocannabinoid	oleoyl ethanolamide	3.55	0.000	0.000
		palmitoyl ethanolamide	1.69	0.007	0.004
		stearoyl ethanolamide	1.60	0.023	0.011
		arachidonoyl ethanolamide	3.13	0.022	0.010
		N-myristoyltaurine*	2.91	0.036	0.016
		N-arachidonoyltaurine	5.96	0.053	0.022
		N-oleoyltaurine	11.58	0.007	0.004
		N-stearoyltaurine	8.88	0.013	0.006
		N-palmitoyltaurine	9.83	0.013	0.006
		N-linoleoyltaurine*	7.98	0.026	0.012
		linoleoyl ethanolamide	4.89	0.068	0.028
		palmitoleoyl ethanolamide*	1.43	0.379	0.127
		N-oleoylserine	0.74	0.794	0.243
	Inositol Metabolism	myo-inositol	1.09	0.268	0.094
		inositol 1-phosphate (I1P)	6.60	0.000	0.000
	Phospholipid Metabolism	choline	2.03	0.000	0.000
		choline phosphate	1.04	0.637	0.200
		cytidine 5'-diphosphocholine	2.00	0.000	0.000
		glycerophosphorylcholine (GPC)	6.62	0.000	0.000
		phosphoethanolamine	37.36	0.000	0.000
		cytidine-5'-diphosphoethanolamine	2.98	0.000	0.000
		glycerophosphoethanolamine	3.64	0.000	0.000
		glycerophosphoserine*	0.42	0.000	0.000
		glycerophosphoinositol*	1.77	0.000	0.000
		trimethylamine N-oxide	0.55	0.000	0.000
	Phosphatidylcholine (PC)	1-myristoyl-2-palmitoyl-GPC (14:0/16:0)	1.51	0.004	0.003
		1-myristoyl-2-arachidonoyl-GPC (14:0/20:4)*	4.62	0.000	0.000
		1,2-dipalmitoyl-GPC (16:0/16:0)	1.84	0.001	0.000
		1-palmitoyl-2-palmitoleoyl-GPC (16:0/16:1)*	1.45	0.009	0.005
		1-palmitoyl-2-stearoyl-GPC (16:0/18:0)	1.31	0.153	0.058
		1-palmitoyl-2-oleoyl-GPC (16:0/18:1)	1.82	0.000	0.000
		1-palmitoyl-2-arachidonoyl-GPC (16:0/20:4n6)	5.92	0.000	0.000
		1-palmitoyl-2-docosahexaenoyl-GPC (16:0/22:6)	2.98	0.000	0.000
		1-palmitoleoyl-2-linolenoyl-GPC (16:1/18:3)*	0.18	0.000	0.000
		1-stearoyl-2-oleoyl-GPC (18:0/18:1)	2.19	0.000	0.000
		1-stearoyl-2-linoleoyl-GPC (18:0/18:2)*	2.17	0.000	0.000
		1-stearoyl-2-arachidonoyl-GPC (18:0/20:4)	11.00	0.000	0.000
		1-stearoyl-2-docosahexaenoyl-GPC (18:0/22:6)	5.43	0.000	0.000
		1,2-dioleoyl-GPC (18:1/18:1)	1.68	0.004	0.002
Lipid		1-oleoyl-2-docosahexaenoyl-GPC (18:1/22:6)*	2.91	0.000	0.000
		1,2-dilinoleoyl-GPC (18:2/18:2)	1.78	0.015	0.007
		1,2-dipalmitoyl-GPE (16:0/16:0)*	1.52	0.023	0.011
		1-palmitoyl-2-oleoyl-GPE (16:0/18:1)	1.03	0.987	0.291
		1-palmitoyl-2-arachidonoyl-GPE (16:0/20:4)*	2.00	0.001	0.001
		1-palmitoyl-2-docosahexaenoyl-GPE (16:0/22:6)*	3.73	0.000	0.000

Phosphatidylethanolamine (PE)	1-stearoyl-2-oleoyl-GPE (18:0/18:1)	2.03	0.000	0.000
	1-stearoyl-2-arachidonoyl-GPE (18:0/20:4)	3.33	0.000	0.000
	1-oleoyl-2-linoleoyl-GPE (18:1/18:2)*	1.31	0.051	0.022
	1-oleoyl-2-arachidonoyl-GPE (18:1/20:4)*	3.78	0.000	0.000
	1-oleoyl-2-docosahexaenoyl-GPE (18:1/22:6)*	5.98	0.000	0.000
Phosphatidylserine (PS)	1-palmitoyl-2-oleoyl-GPS (16:0/18:1)	0.86	0.221	0.080
	1-stearoyl-2-oleoyl-GPS (18:0/18:1)	2.20	0.000	0.000
	1-stearoyl-2-arachidonoyl-GPS (18:0/20:4)	3.00	0.000	0.000
Phosphatidylglycerol (PG)	1-palmitoyl-2-oleoyl-GPG (16:0/18:1)	2.31	0.011	0.005
Phosphatidylinositol (PI)	1-palmitoyl-2-oleoyl-GPI (16:0/18:1)*	1.54	0.004	0.002
	1-palmitoyl-2-arachidonoyl-GPI (16:0/20:4)*	1.87	0.037	0.016
	1-stearoyl-2-oleoyl-GPI (18:0/18:1)*	1.68	0.547	0.177
	1-stearoyl-2-arachidonoyl-GPI (18:0/20:4)	3.79	0.001	0.001
	1-oleoyl-2-arachidonoyl-GPI (18:1/20:4)*	3.18	0.000	0.000
Lysophospholipid	1-palmitoyl-GPC (16:0)	1.38	0.057	0.024
	2-palmitoyl-GPC (16:0)*	2.18	0.204	0.074
	1-palmitoleoyl-GPC (16:1)*	1.07	0.585	0.186
	2-palmitoleoyl-GPC (16:1)*	0.90	0.378	0.127
	1-stearoyl-GPC (18:0)	3.82	0.000	0.000
	1-oleoyl-GPC (18:1)	1.58	0.019	0.009
	1-lignoceroyl-GPC (24:0)	1.13	0.738	0.228
	1-palmitoyl-GPE (16:0)	1.81	0.033	0.015
	1-stearoyl-GPE (18:0)	3.50	0.000	0.000
	2-stearoyl-GPE (18:0)*	3.40	0.063	0.026
	1-oleoyl-GPE (18:1)	2.54	0.000	0.000
	1-linoleoyl-GPE (18:2)*	1.61	0.001	0.000
	1-arachidonoyl-GPE (20:4n6)*	3.38	0.000	0.000
	1-palmitoyl-GPS (16:0)*	0.40	0.376	0.127
	1-stearoyl-GPS (18:0)*	1.88	0.028	0.013
	1-oleoyl-GPS (18:1)	3.90	0.053	0.022
	1-palmitoyl-GPG (16:0)*	1.15	0.737	0.228
	1-stearoyl-GPG (18:0)	0.66	0.573	0.183
	1-oleoyl-GPG (18:1)*	1.23	0.144	0.054
	1-palmitoyl-GPI (16:0)	12.57	0.004	0.002
	1-stearoyl-GPI (18:0)	6.07	0.026	0.012
	1-oleoyl-GPI (18:1)	9.62	0.000	0.000
	1-arachidonoyl-GPI (20:4)*	47.22	0.000	0.000
Plasmalogen	1-(1-enyl-palmitoyl)-2-oleoyl-GPE (P-16:0/18:1)*	1.97	0.000	0.000
	1-(1-enyl-palmitoyl)-2-linoleoyl-GPE (P-16:0/18:2)*	1.56	0.004	0.002
	1-(1-enyl-palmitoyl)-2-palmitoyl-GPC (P-16:0/16:0)*	2.55	0.000	0.000
	1-(1-enyl-palmitoyl)-2-palmitoleoyl-GPC (P-16:0/16:1)*	1.67	0.005	0.003
	1-(1-enyl-palmitoyl)-2-arachidonoyl-GPE (P-16:0/20:4)*	2.79	0.000	0.000
	1-(1-enyl-palmitoyl)-2-oleoyl-GPC (P-16:0/18:1)*	1.60	0.011	0.006
	1-(1-enyl-stearoyl)-2-oleoyl-GPE (P-18:0/18:1)	2.07	0.006	0.004
	1-(1-enyl-palmitoyl)-2-arachidonoyl-GPC (P-16:0/20:4)*	11.29	0.000	0.000
	1-(1-enyl-palmitoyl)-2-linoleoyl-GPC (P-16:0/18:2)*	1.16	0.642	0.201
	1-(1-enyl-stearoyl)-2-arachidonoyl-GPE (P-18:0/20:4)*	4.54	0.000	0.000
Lysoplasmalogen	1-(1-enyl-palmitoyl)-GPC (P-16:0)*	2.11	0.001	0.001
	1-(1-enyl-palmitoyl)-GPE (P-16:0)*	4.81	0.000	0.000
	1-(1-enyl-oleoyl)-GPE (P-18:1)*	6.81	0.000	0.000
	1-(1-enyl-stearoyl)-GPE (P-18:0)*	5.38	0.000	0.000
Glycerolipid Metabolism	glycerol	0.81	0.033	0.015
	glycerol 3-phosphate	5.10	0.000	0.000
	glycerophosphoglycerol	0.96	0.714	0.222
Monoacylglycerol	1-myristoylglycerol (14:0)	4.70	0.013	0.006
	1-pentadecanoylglycerol (15:0)	4.81	0.004	0.002
	1-palmitoylglycerol (16:0)	3.16	0.052	0.022
	1-palmitoleoylglycerol (16:1)*	4.74	0.044	0.019
	1-margaroylglycerol (17:0)	4.54	0.011	0.006
	1-oleoylglycerol (18:1)	6.97	0.016	0.008
	1-linoleoylglycerol (18:2)	11.77	0.002	0.002
	1-dihomo-linolenylglycerol (20:3)	26.02	0.002	0.001
	1-arachidonylglycerol (20:4)	48.16	0.000	0.000
	1-docosahexaenoylglycerol (22:6)	21.85	0.002	0.002
	2-myristoylglycerol (14:0)	4.22	0.028	0.013
	2-palmitoylglycerol (16:0)	3.66	0.486	0.159
	2-palmitoleoylglycerol (16:1)*	5.12	0.035	0.015
	2-oleoylglycerol (18:1)	6.46	0.020	0.009
	2-linoleoylglycerol (18:2)	14.44	0.005	0.003
	2-arachidonoylglycerol (20:4)	35.09	0.001	0.001
	2-docosahexaenoylglycerol (22:6)*	17.97	0.003	0.002
	1-heptadecenoylglycerol (17:1)*	6.26	0.021	0.010
	2-heptadecenoylglycerol (17:1)*	7.95	0.010	0.005
Diacylglycerol	palmitoyl-oleoyl-glycerol (16:0/18:1) [2]*	0.21	0.000	0.000
	oleoyl-arachidonoyl-glycerol (18:1/20:4) [2]*	4.97	0.088	0.035
Galactosyl Glycerolipids	galactosylglycerol	0.12	0.000	0.000
Sphingolipid Synthesis	3-ketosphinganine	0.93	0.438	0.145
	sphinganine	0.93	0.773	0.237
	sphingadienine	6.26	0.000	0.000
	phytosphingosine	0.74	0.102	0.040
Dihydroceramides	N-palmitoyl-sphinganine (d18:0/16:0)	0.72	0.297	0.103
	N-stearoyl-sphinganine (d18:0/18:0)*	1.12	0.944	0.282
Ceramides	N-palmitoyl-sphingosine (d18:1/16:0)	1.21	0.569	0.183
	N-stearoyl-sphingosine (d18:1/18:0)*	2.69	0.002	0.001
	N-palmitoyl-sphingadienine (d18:2/16:0)*	1.07	0.930	0.279
	ceramide (d18:1/14:0, d16:1/16:0)*	0.85	0.280	0.098

		ceramide (d18:1/17:0, d17:1/18:0)*	2.10	0.083	0.034
		ceramide (d16:1/24:1, d18:1/22:1)*	0.66	0.168	0.063
		ceramide (d18:2/24:1, d18:1/24:2)*	1.43	0.281	0.098
	**Hexosylceramides (HCER)**	glycosyl-N-stearoyl-sphinganine (d18:0/18:0)*	4.95	0.016	0.008
		glycosyl-N-palmitoyl-sphingosine (d18:1/16:0)	1.05	0.847	0.258
		glycosyl-N-stearoyl-sphingosine (d18:1/18:0)	3.32	0.001	0.001
		glycosyl-N-behenoyl-sphingadienine (d18:2/22:0)*	1.79	0.077	0.031
		glycosyl ceramide (d18:1/20:0, d16:1/22:0)*	7.63	0.000	0.000
		glycosyl ceramide (d16:1/24:1, d18:1/22:1)*	1.77	0.112	0.044
		glycosyl ceramide (d18:1/23:1, d17:1/24:1)*	1.87	0.091	0.036
		glycosyl ceramide (d18:2/24:1, d18:1/24:2)*	1.82	0.030	0.014
	**Lactosylceramides (LCER)**	lactosyl-N-palmitoyl-sphingosine (d18:1/16:0)	1.93	0.000	0.000
		lactosyl-N-stearoyl-sphingosine (d18:1/18:0)*	4.49	0.000	0.000
		lactosyl-N-behenoyl-sphingosine (d18:1/22:0)*	3.90	0.000	0.000
		lactosyl-N-nervonoyl-sphingosine (d18:1/24:1)*	3.33	0.000	0.000
	**Dihydrosphingomyelins**	myristoyl dihydrosphingomyelin (d18:0/14:0)*	1.49	0.092	0.036
		palmitoyl dihydrosphingomyelin (d18:0/16:0)*	2.16	0.005	0.003
		behenoyl dihydrosphingomyelin (d18:0/22:0)*	2.09	0.063	0.026
		sphingomyelin (d18:0/18:0, d19:0/17:0)*	3.82	0.001	0.001
		sphingomyelin (d18:0/20:0, d16:0/22:0)*	2.29	0.009	0.005
	**Sphingomyelins**	palmitoyl sphingomyelin (d18:1/16:0)	2.26	0.000	0.000
		stearoyl sphingomyelin (d18:1/18:0)	4.80	0.000	0.000
		behenoyl sphingomyelin (d18:1/22:0)*	2.29	0.015	0.008
		tricosanoyl sphingomyelin (d18:1/23:0)*	1.97	0.021	0.010
		lignoceroyl sphingomyelin (d18:1/24:0)	2.51	0.007	0.004
		sphingomyelin (d18:2/23:1)*	2.03	0.007	0.004
		sphingomyelin (d18:2/24:2)*	1.56	0.069	0.028
		sphingomyelin (d17:1/14:0, d16:1/15:0)*	0.45	0.001	0.000
		sphingomyelin (d18:1/14:0, d16:1/16:0)*	1.09	0.763	0.234
		sphingomyelin (d18:2/14:0, d18:1/14:1)*	0.48	0.001	0.000
		sphingomyelin (d17:1/16:0, d18:1/15:0, d16:1/17:0)*	1.20	0.433	0.144
		sphingomyelin (d17:2/16:0, d18:2/15:0)*	1.05	0.859	0.261
		sphingomyelin (d18:2/16:0, d18:1/16:1)*	1.10	0.719	0.223
		sphingomyelin (d18:1/17:0, d17:1/18:0, d19:1/16:0)	3.91	0.000	0.000
		sphingomyelin (d18:1/18:1, d18:2/18:0)	2.97	0.000	0.000
		sphingomyelin (d18:1/20:0, d16:1/22:0)*	5.36	0.000	0.000
		sphingomyelin (d18:1/21:0, d17:1/22:0, d16:1/23:0)*	2.23	0.015	0.007
		sphingomyelin (d18:1/22:1, d18:2/22:0, d16:1/24:1)*	1.89	0.007	0.004
		sphingomyelin (d18:1/22:2, d18:2/22:1, d16:1/24:2)*	1.84	0.135	0.052
		sphingomyelin (d18:2/23:0, d18:1/23:1, d17:1/24:1)*	2.26	0.008	0.004
		sphingomyelin (d18:1/24:1, d18:2/24:0)*	2.57	0.002	0.001
		sphingomyelin (d18:2/24:1, d18:1/24:2)*	2.15	0.001	0.001
	**Sphingosines**	sphingosine	2.72	0.002	0.001
		sphingosine 1-phosphate	1.07	0.749	0.231
		hexadecasphingosine (d16:1)*	2.57	0.010	0.005
		heptadecasphingosine (d17:1)	2.40	0.009	0.005
		eicosanoylsphingosine (d20:1)*	1.37	0.362	0.123
	**Mevalonate Metabolism**	3-hydroxy-3-methylglutarate	0.22	0.000	0.000
	**Sterol**	cholesterol	1.16	0.138	0.053
		7-dehydrocholesterol	3.86	0.000	0.000
		4-cholesten-3-one	1.65	0.341	0.117
		beta-sitosterol	1.00	1.000	0.291
		campesterol	0.94	0.826	0.252
		7-hydroxycholesterol (alpha or beta)	1.99	0.003	0.002
	**Primary Bile Acid Metabolism**	glycochenodeoxycholate	1.00	1.000	0.291
		taurochenodeoxycholate	1.00	1.000	0.291
	**Secondary Bile Acid Metabolism**	glycodeoxycholate	1.00	1.000	0.291
	**Purine Metabolism, (Hypo)Xanthine/Inosine containing**	AICA ribonucleotide	0.19	0.000	0.000
		inosine 5'-monophosphate (IMP)	1.95	0.025	0.012
		inosine	1.57	0.016	0.008
		hypoxanthine	0.97	0.984	0.291
		xanthine	1.00	0.874	0.265
		xanthosine	2.07	0.008	0.004
		N1-methylinosine	13.48	0.000	0.000
		2'-deoxyinosine	0.19	0.000	0.000
		urate	1.21	0.365	0.124
		allantoin	0.94	0.394	0.132
	**Purine Metabolism, Adenine containing**	adenosine 5'-triphosphate (ATP)	11.90	0.000	0.000
		adenosine 5'-diphosphate (ADP)	2.21	0.004	0.002
		adenosine 5'-monophosphate (AMP)	0.61	0.003	0.002
		adenosine 3',5'-cyclic monophosphate (cAMP)	0.38	0.000	0.000
		adenylosuccinate	0.19	0.000	0.000
		adenosine	0.86	0.626	0.197
		adenine	0.50	0.000	0.000
		N1-methyladenosine	4.12	0.000	0.000
		N6-methyladenosine	1.29	0.560	0.180
		N6-carbamoylthreonyladenosine	1.54	0.000	0.000
		2'-deoxyadenosine 5'-diphosphate	0.58	0.008	0.004
		2'-deoxyadenosine 5'-monophosphate	0.03	0.000	0.000
		2'-deoxyadenosine	0.47	0.005	0.003
		diadenosine triphosphate	1.08	0.600	0.190
		N6-succinyladenosine	0.93	0.621	0.195
	**Purine Metabolism, Guanine containing**	guanosine 5'- diphosphate (GDP)	9.15	0.000	0.000
		guanosine 5'- monophosphate (5'-GMP)	0.73	0.017	0.008
		guanosine	2.12	0.001	0.000
		guanine	1.19	0.250	0.089

Nucleotide	Purine Metabolism, Guanine containing	7-methylguanine	1.09	0.317	0.110
		N2-methylguanosine	2.41	0.000	0.000
		N2,N2-dimethylguanosine	1.93	0.000	0.000
		2'-deoxyguanosine	0.48	0.002	0.002
	Pyrimidine Metabolism, Orotate containing	dihydroorotate	0.16	0.003	0.002
		orotate	0.03	0.000	0.000
		orotidine	0.04	0.000	0.000
	Pyrimidine Metabolism, Uracil containing	uridine 5'-triphosphate (UTP)	12.44	0.000	0.000
		uridine 5'-diphosphate (UDP)	3.30	0.001	0.001
		uridine 5'-monophosphate (UMP)	0.73	0.076	0.031
		uridine 3'-monophosphate (3'-UMP)	3.89	0.001	0.001
		uridine	1.20	0.113	0.044
		uracil	0.40	0.003	0.002
		pseudouridine	1.97	0.000	0.000
		5,6-dihydrouridine	2.52	0.000	0.000
		2'-O-methyluridine	1.94	0.000	0.000
		5-methyluridine (ribothymidine)	0.47	0.002	0.002
		2'-deoxyuridine	7.77	0.000	0.000
		3-ureidopropionate	0.49	0.000	0.000
		beta-alanine	0.19	0.000	0.000
		3-(3-amino-3-carboxypropyl)uridine*	0.78	0.004	0.003
	Pyrimidine Metabolism, Cytidine containing	cytidine triphosphate	20.20	0.000	0.000
		cytidine diphosphate	4.18	0.000	0.000
		cytidine 5'-monophosphate (5'-CMP)	0.61	0.000	0.000
		cytidine	2.26	0.000	0.000
		cytosine	5.58	0.000	0.000
		3-methylcytidine	4.62	0.000	0.000
		5-methylcytidine	1.12	0.154	0.058
		2'-deoxycytidine 5'-monophosphate	0.19	0.000	0.000
		2'-deoxycytidine	0.24	0.000	0.000
		2'-O-methylcytidine	6.39	0.000	0.000
	Pyrimidine Metabolism, Thymine containing	thymidine 5'-monophosphate	0.09	0.000	0.000
		thymidine	1.03	0.972	0.289
		thymine	0.55	0.008	0.004
		5,6-dihydrothymine	0.91	0.006	0.004
		3-aminoisobutyrate	2.31	0.000	0.000
	Purine and Pyrimidine Metabolism	methylphosphate	1.50	0.041	0.018
Cofactors and Vitamins	Nicotinate and Nicotinamide Metabolism	quinolinate	0.79	0.506	0.165
		nicotinamide	2.00	0.000	0.000
		nicotinamide ribonucleotide (NMN)	1.61	0.022	0.010
		nicotinamide riboside	2.32	0.000	0.000
		nicotinamide adenine dinucleotide (NAD+)	0.84	0.036	0.016
		nicotinamide adenine dinucleotide reduced (NADH)	0.42	0.001	0.001
		nicotinamide adenine dinucleotide phosphate reduced (N	5.37	0.001	0.001
		1-methylnicotinamide	1.98	0.000	0.000
		trigonelline (N'-methylnicotinate)	0.87	0.248	0.088
		adenosine 5'-diphosphoribose (ADP-ribose)	9.84	0.000	0.000
	Riboflavin Metabolism	riboflavin (Vitamin B2)	1.14	0.071	0.029
		flavin adenine dinucleotide (FAD)	1.40	0.002	0.001
		flavin mononucleotide (FMN)	0.89	0.275	0.096
	Pantothenate and CoA Metabolism	pantoate	1.50	0.008	0.005
		pantothenate	1.38	0.003	0.002
		pantetheine	3.29	0.000	0.000
		phosphopantetheine	1.21	0.226	0.082
		3'-dephosphocoenzyme A	1.94	0.017	0.008
		coenzyme A	1.22	0.198	0.072
	Ascorbate and Aldarate Metabolism	2-O-methylascorbic acid	1.87	0.000	0.000
		threonate	1.37	0.012	0.006
		gulonate*	0.07	0.000	0.000
	Tocopherol Metabolism	alpha-tocopherol	0.95	0.580	0.185
	Biotin Metabolism	biotin	0.73	0.268	0.094
	Folate Metabolism	folate	1.00	1.000	0.291
		5-methyltetrahydrofolate (5MeTHF)	0.35	0.000	0.000
	Pterin Metabolism	pterin	2.01	0.023	0.011
	Hemoglobin and Porphyrin Metabolism	bilirubin (Z,Z)	0.52	0.007	0.004
	Thiamine Metabolism	thiamin (Vitamin B1)	3.69	0.000	0.000
		thiamin monophosphate	20.97	0.000	0.000
		thiamin diphosphate	1.84	0.052	0.022
		5-(2-Hydroxyethyl)-4-methylthiazole	3.55	0.000	0.000
	Vitamin A Metabolism	retinol (Vitamin A)	7.71	0.000	0.000
	Vitamin B6 Metabolism	pyridoxine (Vitamin B6)	1.30	0.030	0.014
		pyridoxamine	1.80	0.002	0.001
		pyridoxamine phosphate	1.93	0.000	0.000
		pyridoxal phosphate	1.01	0.980	0.291
		pyridoxal	1.52	0.001	0.001
		pyridoxate	5.33	0.000	0.000
	Benzoate Metabolism	hippurate	3.92	0.000	0.000
		3-hydroxyhippurate	3.72	0.005	0.003
		benzoate	1.38	0.244	0.087
		catechol sulfate	3.05	0.001	0.001
		guaiacol sulfate	1.44	0.113	0.044
		4-methylcatechol sulfate	3.24	0.000	0.000
		p-cresol sulfate	3.65	0.000	0.000
		3-formylindole	1.61	0.013	0.006
		gluconate	5.99	0.000	0.000
		beta-guanidinopropanoate	0.57	0.003	0.002

**Xenobiotics**	**Food Component/Plant**	ergothioneine	0.80	0.140	0.053
		erythritol	**0.55**	0.000	0.000
		homostachydrine*	**0.46**	0.000	0.000
		mannonate*	**0.74**	0.008	0.004
		stachydrine	**0.57**	0.000	0.000
		methyl glucopyranoside (alpha + beta)	**6.36**	0.000	0.000
		ethyl beta-glucopyranoside	1.00	0.942	0.281
		2-aminophenol sulfate	**0.28**	0.000	0.000
	**Drug - Antibiotic**	penicillin G	**3.43**	0.000	0.000
	**Chemical**	sulfate*	**6.30**	0.000	0.000
		O-sulfo-L-tyrosine	**0.65**	0.000	0.000
		2,4-di-tert-butylphenol	1.29	0.394	0.132
		phenol red	**2.69**	0.000	0.000
		thioproline	**1.63**	0.000	0.000
		4-chlorobenzoic acid	**2.19**	0.020	0.009
		branched-chain, straight-chain, or cyclopropyl 12:1 fatty a	1.35	0.318	0.110
